# Cobalt-catalyzed directed C–H alkenylation of pivalophenone N–H imine with alkenyl phosphates

**DOI:** 10.3762/bjoc.14.60

**Published:** 2018-03-28

**Authors:** Wengang Xu, Naohiko Yoshikai

**Affiliations:** 1 Division of Chemistry and Biological Chemistry, School of Physical and Mathematical Sciences, Nanyang Technological University, Singapore 637371, Singapore

**Keywords:** alkenylation, C–C bond formation, C–H activation, cobalt, imine

## Abstract

A cobalt–N-heterocyclic carbene (NHC) catalyst efficiently promotes an *ortho* C–H alkenylation reaction of pivalophenone N–H imine with an alkenyl phosphate. The reaction tolerates various substituted pivalophenone N–H imines as well as cyclic and acyclic alkenyl phosphates.

## Introduction

Transition-metal-catalyzed, directing group-assisted arene C–H activation reactions have been extensively studied over the last few decades to offer a broad array of atom and step-economical methods for the synthesis of functionalized aromatic compounds [1–6]. Among various C–H transformations, the introduction of alkenyl groups into the *ortho* position of functionalized arenes has attracted significant attention because of the synthetic versatility of alkenyl groups. The C–H alkenylation has been achieved most extensively by way of the dehydrogenative Heck-type reaction of olefins [7–9]. Meanwhile, the hydroarylation of alkynes has also been explored as an alternative approach for C–H alkenylation [10]. Despite the significant progress made, each of these C–H alkenylation manifolds has some critical limitations. For example, the dehydrogenative Heck reaction is often limited to activated monosubstituted alkenes (e.g., acrylates), and is challenging with unactivated and multisubstituted alkenes [11]. The hydroarylation of alkynes does not allow for the introduction of cycloalkenyl groups because of the unavailability of the corresponding alkynes. In light of such limitations, a coupling between arene substrates and alkenyl electrophiles would offer a complementary approach for the C–H alkenylation [12]. In particular, C–H alkenylations by way of alkenyl C–O bond cleavage has attracted much attention because of the ready accessibility of the corresponding alkenyl electrophiles (e.g., acetate, phosphate) from ketones [13–17].

**Scheme 1:**
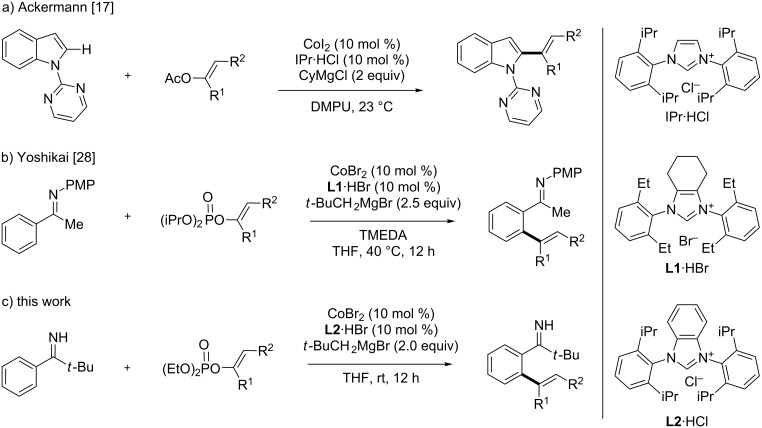
Cobalt–NHC-catalyzed C–H alkenylation reactions with alkenyl electrophiles.

Over the last several years, we and others have developed a series of directed arene C–H functionalization reactions with organic electrophiles under low-valent cobalt catalysis [18–21]. In particular, our group and the Ackermann group have independently demonstrated that the combination of a cobalt–N-heterocyclic carbene (NHC) catalyst and a Grignard reagent allows for the arene C–H functionalization with organic halides and pseudohalides under the assistance of nitrogen directing groups [17,22–27]. In this connection, Ackermann developed a mild and efficient C–H alkenylation of *N*-pyrimidylindoles and pyrroles with alkenyl acetates using a cobalt–NHC catalyst (Scheme 1a) [17]. The same catalytic system also promoted the alkenylation using alkenyl carbamates, carbonates, and phosphates. More recently, we have achieved an N-arylimine-directed arene C–H alkenylation reaction with alkenyl phosphates using a different cobalt–NHC catalyst (Scheme 1b) [28]. Meanwhile, we have also demonstrated that pivaloyl N–H imine serves as a powerful directing group for cobalt-catalyzed arene C–H functionalization reactions such as the hydroarylation of alkenes and alkylation/arylation using organic halides [29–30]. These previous studies have prompted us to expand the scope of cobalt catalysis for the C–H alkenylation and thus to develop an *ortho* C–H alkenylation reaction of pivalophenone N–H imine with alkenyl phosphates using a new cobalt–NHC catalyst, which is reported herein (Scheme 1c). The present alkenylation features a mild reaction temperature and displays applicability to a variety of substituted pivalophenone N–H imines and alkenyl phosphates. It should be emphasized that pivalophenone N–H imines and related bulky N–H imines can be readily prepared from the corresponding aryl nitriles and organolithium or Grignard reagents, while analogous N-substituted imines are nontrivial to synthesize because of sluggish ketone/amine condensation. As such, the present reaction would complement the N-arylimine-directed alkenylation.

## Results and Discussion

The present study commenced with screening of the reaction conditions for the coupling between pivalophenone N–H imine **1a** and cyclohexenyl phosphate **2a** (Table 1). Thus, the reaction was performed in the presence of CoBr_2_ (10 mol %), ligand (10–20 mol %), and *t*-BuCH_2_MgBr (2 equiv) in THF at room temperature. While monodentate phosphines such as PPh_3_ and PCy_3_ were entirely ineffective (Table 1, entries 1 and 2), common bulky NHC precursors such as 1,3-bis(2,4,6-trimethylphenyl)imidazolium chloride (IMes·HCl) and 1,3-bis(2,6-diisopropylphenyl)imidazolium chloride (IPr·HCl) promoted the coupling reaction to afford the desired alkenylation product **3aa** albeit in moderate yields (Table 1, entries 3 and 4). No significant improvement was observed using the saturated analogues of IMes·HCl and IPr·HCl (Table 1, entries 5 and 6) or the 2,6-diethylphenyl analogue (IEt·HCl, Table 1, entry 7). Furthermore, the NHC precursor featuring a cyclohexane backbone and 2,6-diethylphenyl groups (**L1**·HBr), which proved to be the optimal ligand for the C–H arylation of pivalophenone N–H imine as well as for the C–H alkenylation of N-arylimine (Scheme 1a, b) [28–29], was not particularly effective for the present reaction (Table 1, entry 8). To our delight, we observed a remarkable improvement in the reaction efficiency using the benzofused analogue of IPr·HCl (**L2**·HCl), affording **3aa** in 88% yield without any trace of a dialkenylation product (Table 1, entry 9). It should be noted that, unlike the C–H arylation of pivalophenone N–H imine and the C–H alkenylation of N-arylimine (Scheme 1a, b), the addition of TMEDA was not necessary to achieve high reaction efficiency, while the reason for this remains unclear. Note also that the present reaction could employ the relatively inexpensive diethyl phosphate, whereas, in the N-arylimine-directed alkenylation, the use of diisopropyl phosphate was necessary to achieve higher and more reproducible yields (cf. Scheme 1b) [28].

**Table 1:** Optimization of reaction conditions.^a^

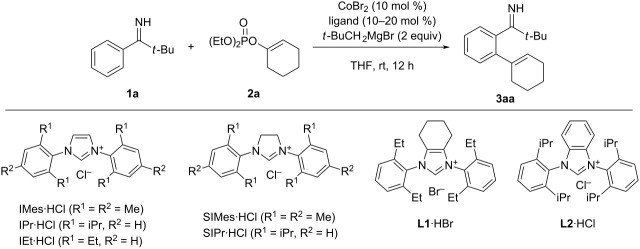		

entry	ligand (mol %)	yield (%)^b^

1	PPh_3_ (20)	0
2	PCy_3_ (20)	0
3	IMes·HCl (10)	29
4	IPr·HCl (10)	53
5	SIMes·HCl (10)	40
6	SIPr·HCl (10)	46
7	IEt·HCl (10)	38
8	**L1**·HBr (10)	37
9	**L2**·HCl (10)	88^c^

^a^The reaction was performed using 0.2 mmol of **1a** and 0.3 mmol (1.5 equiv) of **2a**. ^b^Determined by GC using *n*-tridecane as an internal standard. ^c^Isolated yield.

**Scheme 2:**
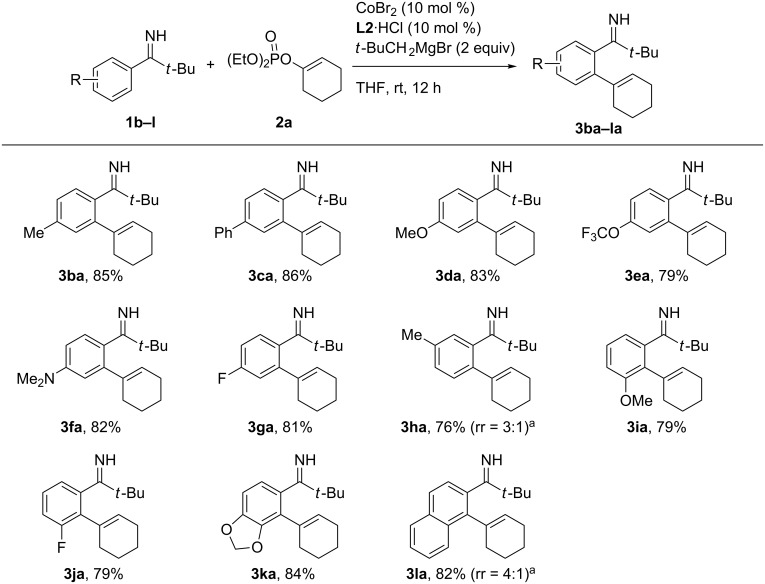
Reaction of substituted pivalophenone N–H imines with **2a.** ^a^The major regioisomer is shown (rr = regioisomer ratio).

With the optimized reaction conditions in hand, we explored the scope of the present alkenylation reaction. First, various substituted pivalophenone N–H imines were subjected to the reaction with **2a** (Scheme 2). Pivalophenone N–H imines bearing a series of *para*-substituents all participated in the alkenylation reaction to afford the desired products **3ba**–**ga** in good yields. The reaction of *m*-methyl-substituted imine took place preferentially at the less hindered position to afford **3ha** as the major isomer with a moderate regioselectivity of 3:1. By contrast, imines bearing *m*-methoxy, *m*-fluoro, or a 3,4-methylenedioxy group underwent exclusive alkenylation at the proximity of the functional group to afford the products **3ia**–**ka** in good yields. As was also observed in previously reported cobalt-catalyzed *ortho* C–H functionalization reactions [22–23,28–29], this regioselectivity may be ascribed to the role of the oxygen or fluorine atom as a secondary directing group to have an electrostatic interaction with the cobalt center during the C–H activation. For compound **3ja**, an increased acidity of the *ortho* position of the fluorine atom could have also contributed to the observed regioselectivity [31]. Curiously, the reaction of 2-naphthylimine resulted in the preferential alkenylation of the more hindered 1-position rather than the 3-position, with a regioselectivity of 4:1 (see **3la**).

**Scheme 3:**
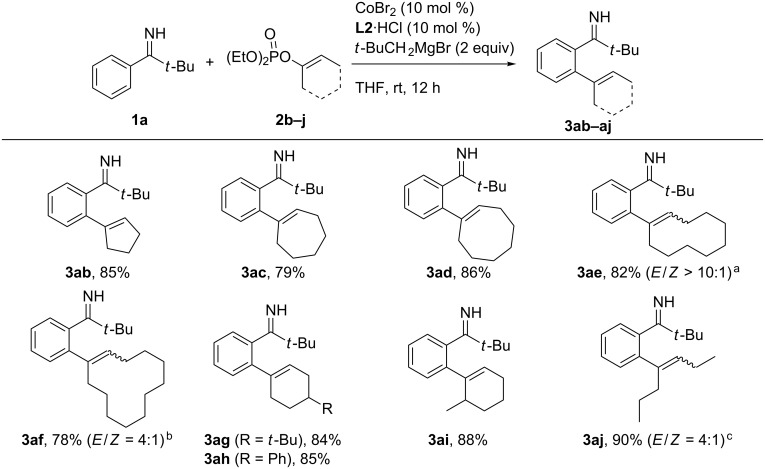
Reaction of **1a** with various alkenyl phosphates. ^a^A mixture of *E*- and *Z*-alkenyl phosphate (ca. 1:1) was used. ^b^*Z*-rich alkenyl phosphate (*Z*/*E* = ca. 9:1) was used. ^c^*E*-alkenyl phosphate was used.

Next, the reaction of the parent pivalophenone N–H imine **1a** with different alkenyl phosphates was explored (Scheme 3). The reaction of cyclopentenyl phosphate proceeded smoothly to afford the desired product **3ab** in a high yield of 85%. This is in a sharp contrast to the poor reactivity of the analogous diisopropyl phosphate in the N–PMP imine-directed alkenylation (12% yield). Other cycloalkenyl phosphates with larger ring sizes also efficiently underwent the C–H alkenylation to afford the respective products **3ac**–**af** in good yields. Notably, the cyclodecenylated product **3ae** was obtained in an *E*-rich form from an *E*/*Z* mixture (1:1) of the starting alkenyl phosphate, demonstrating the *E*/*Z* isomerization during the C–C-bond formation. The *E*/*Z* isomerization was also observed for the conversion of cyclododecenyl phosphate (*E*/*Z* = 9:1) to the product **3af** (*E*/*Z* = 3:1). Expectedly, 4-substituted cyclohexenyl phosphates reacted smoothly to afford the desired products **3ag** and **3ah**. Furthermore, a 6-methyl group on the cyclohexenyl phosphate did not interfere with the reaction (see **3ai**). Finally an acyclic alkenyl phosphate derived from 4-heptanone (*E* isomer) was also amenable to the alkenylation reaction, affording the product **3aj** with an *E*/*Z* ratio of 4:1.

**Scheme 4:**
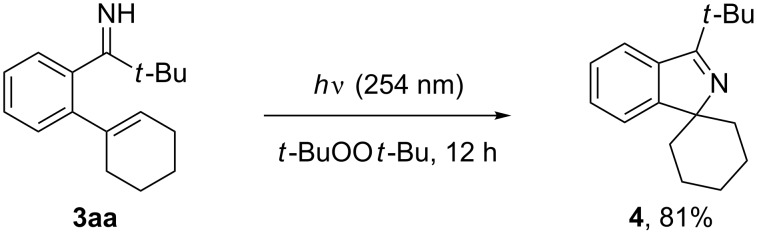
The cyclization of *o*-alkenylpivalophenone N–H imine.

In our previous study on the C–H alkylation and arylation of pivalophenone N–H imines, we demonstrated that the pivaloyl imine readily undergoes fragmentation into a cyano group via an iminyl radical under peroxide photolysis or copper-catalyzed aerobic conditions [29]. Under the same peroxide photolysis conditions (*t*-BuOO*t*-Bu with UV (254 nm) irradiation), the *ortho*-alkenylated imine **3aa** underwent a C–N bond-forming cyclization to afford the spirocyclic imine **4** in 81% yield (Scheme 4). The reaction likely involves the initial formation of an iminyl radical from **3aa** and a *tert*-butoxyl radical and its intramolecular addition to the cyclohexenyl group.

**Scheme 5:**
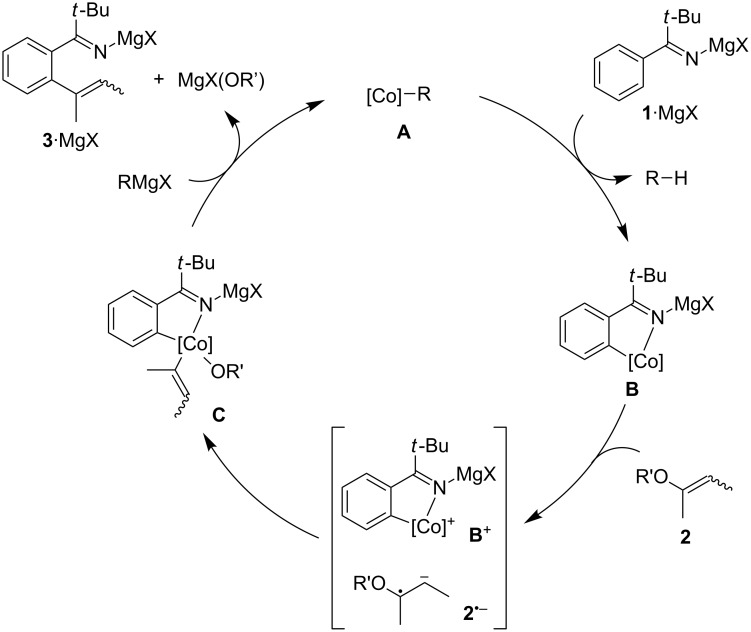
Proposed catalytic cycle (R = *t*-BuCH_2_, R' = P(O)(OEt)_2_).

On the basis of our previous studies on the N-arylimine-directed C–H alkenylation and the N–H imine-directed C–H alkylation/arylation [28–29], we are tempted to propose the catalytic cycle illustrated in Scheme 5. An alkylcobalt species **A**, generated from the cobalt precatalyst and the Grignard reagent, would undergo cyclometalation of magnesium alkylidene amide **1**·MgX, generated from imine **1** and the Grignard reagent, to give a cobaltacycle species **B** while liberating an alkane R–H. The species **B** would then undergo a single-electron transfer (SET) to the alkenyl phosphate **2** to generate a pair of an oxidized cobaltacycle **B**^+^ and a radical anion **2**^•−^. This would be followed by the elimination of a phosphate anion and immediate recombination of the cobalt center and the alkenyl radical to give a diorganocobalt intermediate **C**. The C–C-bond rotation of the radical anion **2**^•−^ or the transiently formed alkenyl radical might be responsible for the stereochemical mutation of the C=C bond observed in some cases. The reductive elimination of **C** and subsequent transmetalation with the Grignard reagent would furnish the alkenylation product **3**·MgX and regenerate the species **A**. While the relationship between the ligand and the catalytic activity remains unclear, we speculate that a strong σ-donating ability of NHC ligands would facilitate the SET step among others.

## Conclusion

In summary, we have developed an *ortho* C–H alkenylation reaction of pivalophenone N–H imines with alkenyl phosphates using a cobalt–NHC catalyst. The reaction takes place smoothly at room temperature and is applicable to a variety of substituted pivalophenone N–H imines and alkenyl phosphates. The NHC ligand architecture proved to have a significant impact on the efficiency of the present C–H/electrophile coupling. We anticipate that the elaboration of NHC ligands would also be instrumental to the improvement of other C–H activation and related transformations promoted by low-valent cobalt complexes [32–40].

## Experimental

**Typical procedure:** Cobalt-catalyzed alkenylation of pivalophenone N–H imine **1a** with alkenyl phosphate **2a**. A 10 mL Schlenk tube equipped with a magnetic stirring bar was charged with **L2**·HCl (9.5 mg, 0.020 mmol), CoBr_2_ (4.4 mg, 0.020 mmol), and THF (0.30 mL). The resulting solution was cooled in an ice bath, followed by the addition of *t*-BuCH_2_MgBr (2.0 M in THF, 0.20 mL, 0.40 mmol). After stirring for 30 min, 2,2-dimethyl-1-phenylpropan-1-imine (**1a**, 33 mg, 0.20 mmol) and cyclohex-1-en-1-yl diethyl phosphate (**2a**, 70 mg, 0.30 mmol) were added. The resulting mixture was warmed to room temperature, stirred for 12 h, and then filtered through a short silica-gel column, which was washed with ethyl acetate (5 mL). The filtrate was concentrated under reduced pressure. Silica gel chromatography (eluent: hexane/EtOAc/NEt_3_ 50:1:1) of the crude product afforded the desired alkenylation product as a colorless oil (43 mg, 88%).

## Supporting Information

File 1 Experimental details and characterization data of new compounds.
