# Supplementary material for: Cobalt-catalyzed directed C–H alkenylation of pivalophenone N–H imine with alkenyl phosphates
Source: Beilstein J Org Chem. 2018 Mar 28;14:709–15. doi: 10.3762/bjoc.14.60 (PMC5905286; doi:10.3762/bjoc.14.60)
Supplement: File 1 — Experimental details and characterization data of new compounds. [file Beilstein_J_Org_Chem-14-709-s001.pdf]

Supporting Information

for

Cobalt-catalyzed directed C–H alkenylation of pivalophenone N–H imine with alkenyl phosphates

Wengang Xu and Naohiko Yoshikai*

Address: Division of Chemistry and Biological Chemistry, School of Physical and Mathematical Sciences, Nanyang Technological University, Singapore 637371, Singapore

Email: Naohiko Yoshikai - nyoshikai@ntu.edu.sg

*Corresponding author

Experimental details and characterization data of new compounds

Table of Contents

Material and Methods S2

Preparation of Starting Materials S3

Cobalt-Catalyzed C–H Alkenylation of Pivalophenone Imines with Alkenyl Phosphates S6

Cyclization of Alkenylated N–H Imine under Peroxide Photolysis S15

References S16

NMR Spectra S17

Material and methods

General. All reactions dealing with air and moisture-sensitive compounds were carried out in oven-dried reaction vessels under nitrogen atmosphere. Analytical thin layer chromatography (TLC) was performed on Merck 60 F254 silica gel plates. Flash column chromatography was performed using 40–63μm silica gel (Si 60, Merck). ^{1}H, ^{13}C and ^{31}P nuclear magnetic resonance (NMR) spectra were recorded on a JEOL ECA-400 (400 MHz), Bruker AV-300 (300 MHz), or AV-400 (400 MHz) NMR spectrometer. ^{1}H and ^{13}C NMR spectra are reported in parts per million (ppm) downfield from the internal standard, tetramethylsilane (0 ppm), and ^{31}P NMR spectra are reported in reference to an external standard, 85% phosphoric acid (0 ppm). Gas chromatography (GC) analysis was performed on a Shimadzu GC-2010 system equipped with glass capillary column DB-5 (Agilent J&W, 0.25 mm id × 30 m, 0.25 μm film thickness). High-resolution mass spectra (HRMS) were obtained with a Q-Tof Premier LC HR mass spectrometer. Melting points were determined using a capillary melting point apparatus and are uncorrected.

Materials. Unless otherwise noted, commercial reagents were purchased from Aldrich, Alfa Aesar, and other commercial suppliers and were used as received. THF was distilled over Na/benzophenone. Anhydrous $CoBr_2$ (>98%) was purchased from Alfa Aesar and used as received. Grignard reagents were prepared from the corresponding alkyl halides and magnesium turnings in THF, and titrated before use. IEt·HCl,[1] **L1**·HBr,[2] and **L2**·HCl[3] were prepared according to literature procedures.

Preparation of the starting materials

Preparation of aryl imines

All imines shown below were synthesized according to the literature procedures[4] and purified by recrystallization or distillation. Spectral data for **1a**[5]**, 1b,c**[6]**, 1d**[4], **1e–g**[6], **1h**[5], and **1i–l**[6], showed good agreement with the literature data.

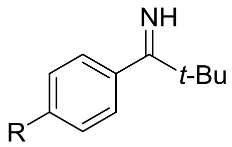

1a (R = H)
1b (R = Me)
1c (R = Ph)
1d (R = OMe)
1e (R = OCF_3)
1f (R = NMe_2)
1g (R = F)

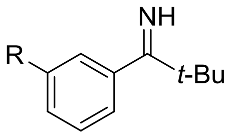

1h (R = Me)
1i (R = OMe)
1j (R = F)

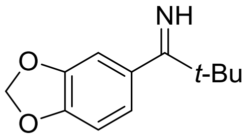

1k

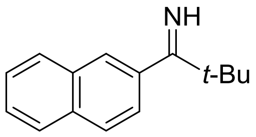

1l

Preparation of alkenyl phosphates

Enol phosphate esters **2a–j** were prepared according to the procedure described by Kumada and coworkers[7]. Spectral data for **2a**[7], **2b**[7], **2c**[8], **2f**[7], and **2i**[7] showed good agreement with the literature data.

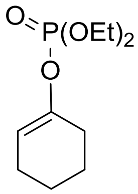

2a

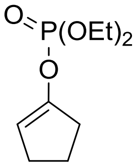

2b

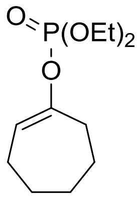

2c

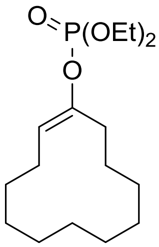

2f

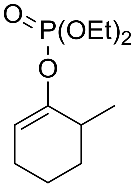

2i

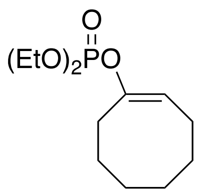

(*E*)-Cyclooct-1-en-1-yl diethyl phosphate (**2d**): Colorless liquid; ^{1}H NMR (400 MHz, $CDCl_3$): δ 5.37 (tt, *J* = 8.6, 2.2 Hz, 1H), 4.14–4.04 (m, 4H), 2.32–2.26 (dd, *J* = 8.2, 4.2 Hz, 2H), 2.06–1.97 (m, 2H), 1.64–1.55 (m, 2H), 1.54–1.43 (m, 6H), 1.33–1.25 (m, 6H); ^{13}C NMR (100 MHz,

$CDCl_3$): δ 149.7 (d, J_{C-P} = 9.3 Hz), 112.7 (d, J_{C-P} = 4.9 Hz), 64.1 (d, J_{C-P} = 6.0 Hz), 30.1, 29.4 (d, J_{C-P} = 4.4 Hz), 27.6, 26.2, 26.0, 25.2, 16.2 (d, J_{C-P} = 6.9 Hz); $^{31}P\{^1H\}$ NMR (162 MHz, $CDCl_3$): δ -5.6; HRMS (ESI) Calcd for $C_{12}H_{24}O_4P$ $[M + H]^+$ 263.1412, found 263.1408.

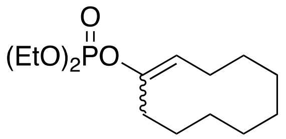

Cyclodec-1-en-1-yl diethyl phosphate (*E*/*Z* = ca. 1:1) (**2e**): Light yellow liquid; ^1H NMR (400 MHz, $CDCl_3$, both isomers): δ 5.21 (td, *J* = 8.7, 2.1 Hz, 1H, minor), 4.99 (td, *J* = 7.8, 0.9 Hz, 1H, major), 4.14–4.04 (m, 4H, both), 2.38–2.09 (m, 4H, both), 1.57–1.25 (m, 18H, both); ^{13}C NMR (100 MHz, $CDCl_3$, both isomers): δ 148.1 (d, *J* = 9.3 Hz), 147.0 (d, *J* = 7.6 Hz), 117.4 (d, *J* = 6.9 Hz), 114.9 (d, *J* = 4.5 Hz), 64.13 (d, *J* = 6.2 Hz), 64.06 (d, *J* = 6.2 Hz), 34.8, 27.4 (d, *J* = 4.3 Hz), 27.1, 26.9, 26.1 (two signals overlapped), 25.5, 25.4, 25.2, 25.0, 24.6 (two signals overlapped), 24.2, 23.9, 20.9, 20.6, 16.22 (d, *J* = 6.6 Hz), 16.18 (d, *J* = 6.6 Hz); $^{31}P\{^1H\}$ NMR (162 MHz, $CDCl_3$): δ -5.4. HRMS (ESI) Calcd for $C_{14}H_{28}O_4P$ $[M + H]^+$ 291.1725, found 291.1728.

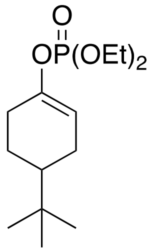

4-(*tert*-Butyl)cyclohex-1-en-1-yl diethyl phosphate (**2g**): Light yellow liquid; ^1H NMR (400 MHz, $CDCl_3$): δ 5.41 (dd, *J* = 5.1, 1.7 Hz, 1H), 4.13-4.09 (m, 4H), 2.28–2.00 (m, 3H), 1.86–1.76 (m, 2H), 1.34–1.21 (m, 8H), 0.83 (s, 9H); ^{13}C NMR (100 MHz, $CDCl_3$): δ 147.8 (d, J_{C-P} = 8.6 Hz), 110.6 (d, J_{C-P} = 5.4 Hz), 64.2 (d, J_{C-P} = 6.0 Hz), 43.5, 32.2, 28.9 (d, J_{C-P} = 3.8 Hz), 27.4, 25.1, 24.1, 16.3 (d, J_{C-P} = 6.8 Hz); $^{31}P\{^1H\}$ NMR (162 MHz, $CDCl_3$): δ -5.4; HRMS (ESI) Calcd for $C_{14}H_{28}O_4P$ $[M + H]^+$ 291.1725, found 291.1729.

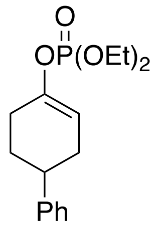

Diethyl (1,2,3,6-tetrahydro-[1,1'-biphenyl]-4-yl) phosphate (**2h**): Light yellow liquid; ^{1}H NMR (400 MHz, $CDCl_3$): δ 7.29 (t, J = 7.5 Hz, 2H), 7.22–7.17 (m, 3H), 5.59–5.55 (m, 1H), 4.21–4.12 (m, 4H), 2.83–2.75 (m, 1H), 2.49–2.19 (m, 4H), 2.03–1.83 (m, 2H), 1.36 (t, J = 7.1 Hz, 6H); ^{13}C NMR (100 MHz, $CDCl_3$): δ 147.5 (d, J_{C-P} = 9.0 Hz), 145.7, 128.4, 126.8, 126.2, 110.1 (d, J_{C-P} = 5.4 Hz), 64.1 (d, J_{C-P} = 6.0 Hz), 39.3, 31.5, 29.6, 28.1 (d, J_{C-P} = 4.1 Hz), 16.1 (d, J_{C-P} = 6.7 Hz); ^{31}P{^{1}H} NMR (162 MHz, $CDCl_3$): δ -5.5; HRMS (ESI) Calcd for $C_{16}H_{24}O_4P$ $[M + H]^+$ 311.1412, found 311.1413.

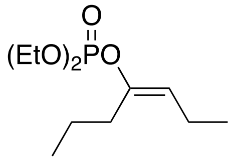

(*E*)-Diethyl hept-3-en-4-yl phosphate (**2j**): Light yellow liquid; ^{1}H NMR (400 MHz, $CDCl_3$): δ 5.45–5.41 (m, 1H), 4.14–4.06 (m, 4H), 2.34 (dd, J = 12.8, 6.0 Hz, 1H), 2.04–1.98 (m, 2H), 1.82–1.74 (m, 1H), 1.63–1.32 (m, 4H), 1.30 (t, J = 7.0 Hz, 6H), 1.05 (d, J = 7.0 Hz, 3H); ^{13}C NMR (100 MHz, $CDCl_3$): δ 151.6 (d, J_{C-P} = 9.6 Hz), 110.1 (d, J_{C-P} = 4.0 Hz), 64.2 (d, J_{C-P} = 5.4 Hz), 32.2 (d, J_{C-P} = 4.7 Hz), 31.4, 24.2, 19.6, 18.4, 16.2 (d, J_{C-P} = 6.8 Hz); ^{31}P{^{1}H} NMR (162 MHz, $CDCl_3$): δ -5.5; HRMS (ESI) Calcd for $C_{11}H_{24}O_4P$ $[M + H]^+$ 251.1412, found 251.1416.

Cobalt-catalyzed C–H alkenylation of pivalophenone imines with alkenyl phosphates

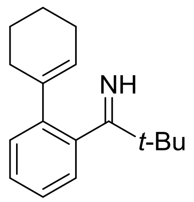

Typical procedure: 2,2-dimethyl-1-(2',3',4',5'-tetrahydro-[1,1'-biphenyl]-2-yl)propan-1-imine (3aa). A 10 mL Schlenk tube equipped with a magnetic stirring bar was charged with **L2**·HCl (9.5 mg, 0.020 mmol), $CoBr_2$ (4.4 mg, 0.020 mmol), and THF (0.30 mL). The resulting solution was cooled in an ice bath, followed by the addition of *t*-$BuCH_2MgBr$ (2.0 M in THF, 0.20 mL, 0.40 mmol). After stirring for 30 min, 2,2-dimethyl-1-phenylpropan-1-imine (**1a**, 33 mg, 0.20 mmol) and cyclohex-1-en-1-yl diethyl phosphate (**2a**, 70 mg, 0.30 mmol) were added. The resulting mixture was warmed to room temperature, stirred for 12 h, and then filtered through a short silica gel column, which was washed with ethyl acetate (5 mL). The filtrate was concentrated under reduced pressure. Silica gel chromatography (eluent: hexane/EtOAc/NEt_3 50:1:1) of the crude product afforded the title compound as a colorless oil (43 mg, 88%).

R_f 0.54 (hexane/EtOAc/NEt_3 = 10/1/1); ^{1}H NMR (400 MHz, $CDCl_3$): δ 9.33 (brs, 1H), 7.28–7.23 (m, 1H), 7.20–7.15 (m, 2H), 7.03–6.99 (m, 1H), 5.70 (brs, 1H), 2.32–2.25 (m, 2H), 2.15–2.09 (m, 2H), 1.75–1.67 (m, 2H), 1.65–1.58 (m, 2H), 1.18 (s, 9H); ^{13}C NMR (100 MHz, $CDCl_3$): δ 191.9, 141.0, 140.5, 137.6, 129.4, 128.1, 127.8, 127.4, 126.0, 40.4, 30.3, 29.4, 25.8, 23.2, 22.0; HRMS (ESI) Calcd for $C_{17}H_{24}N$ $[M + H]^+$ 242.1909, found 242.1910.

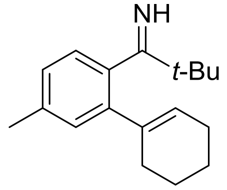

2,2-Dimethyl-1-(5-methyl-2',3',4',5'-tetrahydro-[1,1'-biphenyl]-2-yl)propan-1-imine (3ba): Light yellow oil (43 mg, 85%). R_f 0.53 (hexane/EtOAc/NEt_3 = 10/1/1); ^{1}H NMR (400 MHz, $CDCl_3$): δ 9.24 (brs, 1H), 7.01–6.97 (m, 2H), 6.90 (d, *J* = 7.6 Hz, 1H), 5.67 (t, *J* = 3.4 Hz, 1H), 2.33 (s, 3H), 2.30–2.24 (m, 2H), 2.14–2.08 (m, 2H), 1.74–1.57 (m, 2H), 1.17 (s, 9H); ^{13}C NMR

(100 MHz, $CDCl_3$): δ 192.1, 141.0, 137.8, 137.5, 129.2, 128.9 (two signals overlapped), 127.4, 126.8, 40.4, 30.4, 29.5, 25.9, 23.3, 22.1, 21.4; HRMS (ESI) Calcd for $C_{18}H_{26}N$ $[M + H]^+$ 256.2065, found 256.2062.

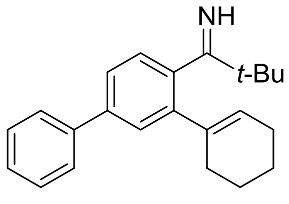

2,2-Dimethyl-1-(2'',3'',4'',5''-tetrahydro-[1,1':3',1''-terphenyl]-4'-yl)propan-1-imine (3ca): Light yellow oil (55 mg, 86%). R_f 0.48 (hexane/EtOAc/NEt_3 = 10/1/1); ^1H NMR (400 MHz, $CDCl_3$): δ 8.91 (brs, 1H), 7.60 (d, J = 7.1 Hz, 2H), 7.46–7.40 (m, 4H), 7.38–7.32 (m, 1H), 7.09 (d, J = 8.6 Hz, 1H), 5.77–5.74 (m, 1H), 2.36–2.30 (m, 2H), 2.17–2.11 (td, J = 6.1, 2.6 Hz, 2H), 1.76–1.70 (m, 2H), 1.67–1.61 (m, 2H), 1.21 (s, 9H); ^{13}C NMR (100 MHz, $CDCl_3$): δ 191.8, 141.6, 140.9, 140.8, 139.5, 137.8, 129.6, 129.0, 128.0, 127.7, 127.3, 127.1, 124.8, 40.5, 30.5, 29.5, 25.9, 23.3, 22.1; HRMS (ESI) Calcd for $C_{23}H_{28}N$ $[M + H]^+$ 318.2222, found 318.2223.

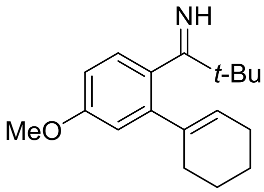

1-(5-Methoxy-2',3',4',5'-tetrahydro-[1,1'-biphenyl]-2-yl)-2,2-dimethylpropan-1-imine (3da): Light yellow oil (46 mg, 83%). R_f 0.37 (hexane/EtOAc/NEt_3 = 10/1/1); ^1H NMR (400 MHz, $CDCl_3$): δ 8.85 (brs, 1H), 6.94 (d, J = 9.2 Hz, 1H), 6.73–6.69 (m, 2H), 5.71–5.68 (m, 1H), 3.79 (s, 3H), 2.28–2.23 (m, 2H), 2.13–2.08 (m, 2H), 1.73–1.65 (m, 2H), 1.64–1.57 (m, 2H), 1.16 (s, 9H); ^{13}C NMR (100 MHz, $CDCl_3$): δ 191.9, 159.0, 142.7, 137.7, 133.3, 129.4, 128.6, 113.5, 111.4, 55.4, 40.5, 30.2, 29.5, 25.8, 23.2, 22.0; HRMS (ESI) Calcd for $C_{18}H_{26}NO$ $[M + H]^+$ 272.2014, found 272.2015.

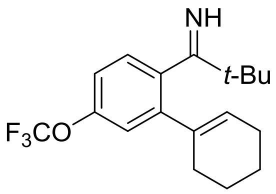

2,2-Dimethyl-1-(5-(trifluoromethoxy)-2',3',4',5'-tetrahydro-[1,1'-biphenyl]-2-yl)propan-1-imine (3ea): Light yellow oil (51 mg, 79%). R_f 0.52 (hexane/EtOAc/NEt_3 = 10/1/1); ^{1}H NMR (400 MHz, $CDCl_3$): δ 9.45 (brs, 1H), 7.04 (s, 3H), 5.75 (s, 1H), 2.30–2.23 (m, 2H), 2.29–2.24 (m, 2H), 1.74–1.59 (m, 4H), 1.16 (s, 9H); ^{13}C NMR (100 MHz, $CDCl_3$): δ 190.9, 148.7, 143.2, 136.7, 130.8 (two signals overlapped), 129.0, 120.7 (d, $^1J_{C\text{-}F}$ = 257.2 Hz), 120.6, 118.4, 40.6, 30.1, 29.3, 25.8, 23.1, 21.9; HRMS (ESI) Calcd for $C_{18}H_{23}NOF_3$ $[M + H]^+$ 326.1732, found 326.1732.

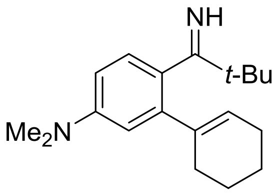

6-(1-Imino-2,2-dimethylpropyl)-*N*,*N*-dimethyl-2',3',4',5'-tetrahydro-[1,1'-biphenyl]-3-amine (3fa): Light yellow oil (47 mg, 82%). R_f 0.45 (hexane/EtOAc/NEt_3 = 10/1/1); ^{1}H NMR (400 MHz, $CDCl_3$): δ 9.45 (brs, 1H), 6.91 (d, J = 8.4 Hz, 1H), 6.54 (dd, J = 8.5, 2.6 Hz, 1H), 6.51 (d, J = 2.5 Hz, 1H), 5.70–5.66 (m, 1H), 2.95 (s, 6H), 2.28–2.24 (m, 2H), 2.14–2.08 (m, 2H), 1.73–1.58 (m, 4H), 1.19 (s, 9H); ^{13}C NMR (100 MHz, $CDCl_3$): δ 192.2, 150.0, 142.3, 138.7, 129.3, 128.6, 128.3, 112.0, 110.0, 40.7, 40.5, 30.4, 29.7, 25.8, 23.3, 22.1; HRMS (ESI) Calcd for $C_{19}H_{29}N_2$ $[M + H]^+$ 285.2331, found 285.2333.

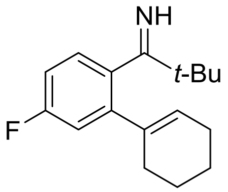

1-(5-Fluoro-2',3',4',5'-tetrahydro-[1,1'-biphenyl]-2-yl)-2,2-dimethylpropan-1-imine (3ga): Light yellow oil (42 mg, 81%). R_f 0.52 (hexane/EtOAc/NEt_3 = 10/1/1); ^{1}H NMR (400 MHz, $CDCl_3$): δ 9.39 (brs, 1H), 6.98 (dd, J = 8.2, 5.9 Hz, 1H), 6.92–6.85 (m, 2H), 5.76–5.72 (m, 1H), 2.28–2.23 (m, 2H), 2.14–2.09 (m, 2H), 1.74–1.67 (m, 2H), 1.65–1.57 (m, 2H), 1.15 (s, 9H); ^{13}C

NMR (100 MHz, $CDCl_3$): δ 191.2, 162.2 (d, $^1J_{C-F}$ = 246.4 Hz), 143.4 (d, $^3J_{C-F}$ = 8.0 Hz), 136.8, 130.4, 129.2 (d, $^3J_{C-F}$ = 7.7 Hz), 114.8 (d, $^2J_{C-F}$ = 21.1 Hz), 113.0 (d, $^2J_{C-F}$ = 21.3 Hz), 40.6, 30.1, 29.4, 25.8, 23.2, 21.9; HRMS (ESI) Calcd for $C_{17}H_{23}NF$ [M + H]$^+$ 260.1815, found 260.1815.

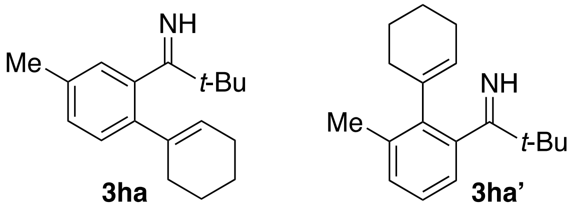

3ha **3ha’**

2,2-Dimethyl-1-(4-methyl-2',3',4',5'-tetrahydro-[1,1'-biphenyl]-2-yl)propan-1-imine (3ha): Light yellow oil (39 mg, 76%, obtained as an inseparable mixture with minor regioisomer **3ha'** (ratio = 3:1 as determined by ^{1}H NMR)). R_f 0.54 (hexane/EtOAc/NEt_3 = 10/1/1); ^{1}H NMR (400 MHz, $CDCl_3$, major isomer): δ 9.28 (s, 1H), 7.07 (d, J = 0.6 Hz, 2H), 6.81 (s, 1H), 5.66 (s, 1H), 2.31 (s, 3H), 2.28–2.22 (m, 2H), 2.13–2.07 (m, 2H), 1.73–1.66 (m, 2H), 1.64–1.57 (m, 2H), 1.17 (s, 9H); ^{13}C NMR (100 MHz, $CDCl_3$, major isomer): δ 192.2, 138.2, 137.5, 135.7, 129.1, 128.6, 128.0, 125.8, 123.4, 40.4, 30.4, 29.5, 25.9, 23.3, 22.1, 21.2; HRMS (ESI) Calcd for $C_{18}H_{26}N$ [M + H]$^+$ 256.2065, found 256.2065.

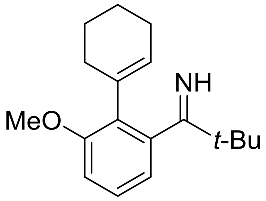

1-(6-Methoxy-2',3',4',5'-tetrahydro-[1,1'-biphenyl]-2-yl)-2,2-dimethylpropan-1-imine (3ia): Light yellow oil (43 mg, 79%). R_f 0.46 (hexane/EtOAc/NEt_3 = 10/1/1); ^{1}H NMR (400 MHz, $CDCl_3$): δ 9.25 (brs, 1H), 7.15 (dd, J = 8.3, 7.7 Hz, 1H), 6.81 (dd, J = 8.4, 1.3 Hz, 1H), 6.66 (dd, J = 7.7, 1.2 Hz, 1H), 5.55–5.50 (m, 1H), 3.80 (s, 3H), 2.29–2.09 (m, 4H), 1.76–1.58 (m, 4H), 1.20 (s, 9H); ^{13}C NMR (100 MHz, $CDCl_3$): δ 190.9, 157.4, 143.2, 135.0, 130.0, 129.3, 127.1, 119.1, 110.2, 56.0, 39.9, 30.0, 29.9, 25.7, 23.1, 22.2; HRMS (ESI) Calcd for $C_{18}H_{26}NO$ [M + H]$^+$ 272.2014, found 272.2017.

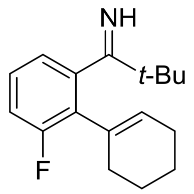

1-(6-Fluoro-2',3',4',5'-tetrahydro-[1,1'-biphenyl]-2-yl)-2,2-dimethylpropan-1-imine (3ja): Light yellow oil (41 mg, 79%). R_f 0.52 (hexane/EtOAc/NEt$_3$ = 10/1/1); ^{1}H NMR (400 MHz, CDCl$_3$): δ 9.26 (brs, 1H), 7.18–7.12 (m, 1H), 7.00–6.95 (m, 1H), 6.83 (d, J = 7.6 Hz, 1H), 5.66–5.62 (m, 1H), 2.21–2.17 (m, 2H), 2.16–2.09 (m, 2H), 1.74–1.60 (m, 4H), 1.18 (s, 9H); ^{13}C NMR (100 MHz, CDCl$_3$): δ 190.0, 160.3 (d, $^1J_{C\text{-}F}$ = 245.8 Hz), 143.5, 132.1, 131.3, 128.8 (d, $^2J_{C\text{-}F}$ = 17.1 Hz), 127.6 (d, $^3J_{C\text{-}F}$ = 8.9 Hz), 122.6, 115.1 (d, $^2J_{C\text{-}F}$ = 23.5 Hz), 40.2, 30.1, 29.6, 25.7, 23.0, 22.0; HRMS (ESI) Calcd for $C_{17}H_{23}NF$ [M + H]$^+$ 260.1815, found 260.1817.

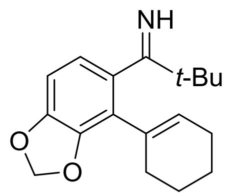

1-(4-(Cyclohex-1-en-1-yl)benzo[*d*][1,3]dioxol-5-yl)-2,2-dimethylpropan-1-imine (3ka): Light yellow oil (48 mg, 84%). R_f 0.45 (hexane/EtOAc/NEt$_3$ = 10/1/1); ^{1}H NMR (400 MHz, CDCl$_3$): δ 8.87 (s, 1H), 6.66 (d, J = 8.0 Hz, 1H), 6.51 (d, J = 8.0 Hz, 1H), 5.94 (s, 2H), 5.73–5.69 (m, 1H), 2.33–2.27 (m, 2H), 2.16–2.10 (m, 2H), 1.73–1.59 (m, 4H), 1.16 (s, 9H); ^{13}C NMR (100 MHz, CDCl$_3$): δ 191.0, 146.8, 144.8, 132.6, 131.2, 123.4, 120.8, 106.4, 101.0, 100.2, 40.4, 29.5, 29.0, 25.8, 23.0, 22.0; HRMS (ESI) Calcd for $C_{17}H_{24}N$ [M + H]$^+$ 242.1909, found 242.1912.

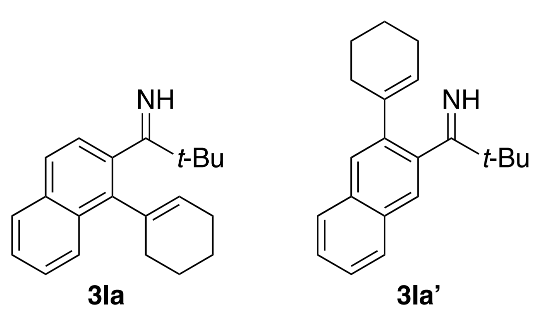

1-(1-(Cyclohex-1-en-1-yl)naphthalen-2-yl)-2,2-dimethylpropan-1-imine (3la): Light yellow oil (48 mg, 82%, obtained as an inseparable mixture with minor regioisomer **3la'** (ratio = 4:1 as determined by ^{1}H NMR)). R_f 0.52 (hexane/EtOAc/NEt$_3$ = 10/1/1); ^{1}H NMR (400 MHz, CDCl$_3$,

major isomer): δ 9.29 (s, 1H), 7.97–7.93 (m, 1H), 7.84–7.77 (m, 1H), 7.70 (d, *J* = 8.4 Hz, 1H), 7.50–7.44 (m, 2H), 7.20 (d, *J* = 8.5 Hz, 1H), 5.72–5.68 (m, 1H), 2.31–2.15 (m, 4H), 1.82–1.63 (m, 4H), 1.28 (s, 9H); ^{13}C NMR (100 MHz, $CDCl_3$, major isomer): δ 191.3, 138.8, 138.1, 136.1, 133.1, 132.4, 129.7, 128.2, 126.6, 126.5, 126.4, 126.1, 124.1, 39.9, 32.3, 30.4, 25.7, 23.2, 22.3; HRMS (ESI) Calcd for $C_{21}H_{26}N$ [M + H]$^+$ 292.2065, found 292.2068.

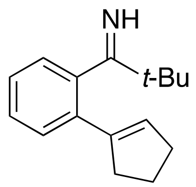

1-(2-(Cyclopent-1-en-1-yl)phenyl)-2,2-dimethylpropan-1-imine (3ab): Light yellow oil (39 mg, 85%). R_f 0.48 (hexane/EtOAc/NEt_3 = 10/1/1); ^{1}H NMR (400 MHz, $CDCl_3$): δ 9.41 (brs, 1H), 7.27–7.25 (m, 2H), 7.20–7.16 (m, 1H), 7.01 (d, J = 7.7 Hz, 1H), 5.85–5.82 (m, 1H), 2.68–2.63 (m, 2H), 2.50–2.41 (m, 2H), 1.99–1.89 (m, 2H), 1.15 (s, 9H); ^{13}C NMR (100 MHz, $CDCl_3$): δ 192.2, 142.6, 135.1, 134.8, 131.8, 127.8, 127.7, 127.5, 126.4, 47.4, 36.6, 34.0, 29.0, 23.7; HRMS (ESI) Calcd for $C_{16}H_{22}N$ [M + H]$^+$ 228.1752, found 228.1757.

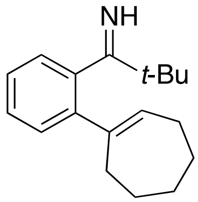

1-(2-(Cyclohept-1-en-1-yl)phenyl)-2,2-dimethylpropan-1-imine (3ac): Light yellow oil (40 mg, 79%). R_f 0.52 (hexane/EtOAc/NEt_3 = 10/1/1); ^{1}H NMR (400 MHz, $CDCl_3$): δ 9.34 (brs, 1H), 7.23 (dd, *J* = 8.1, 1.8 Hz, 1H), 7.18–7.14 (m, 2H), 7.02 (dd, *J* = 7.3, 1.9 Hz, 1H), 5.80 (t, *J* = 6.5 Hz, 1H), 2.45–2.41 (m, 2H), 2.22–2.17 (m, 2H), 1.82–1.76 (m, 2H), 1.67–1.61 (m, 2H), 1.57–1.51 (m, 2H), 1.18 (s, 9H); ^{13}C NMR (100 MHz, $CDCl_3$): δ 191.5, 144.7, 143.3, 134.1, 128.6, 128.2, 127.8, 127.1, 125.9, 43.7, 35.9, 32.7, 29.7, 29.2, 27.4, 26.8; HRMS (ESI) Calcd for $C_{18}H_{26}N$ [M + H]$^+$ 256.2065, found 256.2065.

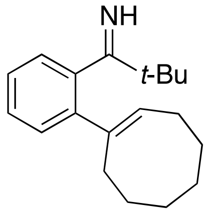

1-(2-(Cyclooct-1-en-1-yl)phenyl)-2,2-dimethylpropan-1-imine (3ad): Light yellow oil (46 mg, 86%). R_f 0.54 (hexane/EtOAc/NEt_3 = 10/1/1); ^{1}H NMR (400 MHz, $CDCl_3$): δ 9.25 (brs, 1H), 7.26–7.23 (m, 1H), 7.21–7.17 (m, 2H), 7.05 (d, J = 7.3 Hz, 1H), 5.64 (t, J = 8.3 Hz, 1H), 2.45–2.40 (m, 2H), 2.24–2.17 (m, 2H), 1.64–1.51 (m, 8H), 1.18 (s, 9H); ^{13}C NMR (100 MHz, $CDCl_3$): δ 191.8, 142.0, 140.9, 140.7, 132.4, 129.2, 127.8, 127.0, 126.1, 40.3, 31.4, 29.8 (two signals overlapped), 29.5, 27.2, 27.0, 26.5; HRMS (ESI) Calcd for $C_{19}H_{28}N$ $[M + H]^+$ 270.2222, found 270.2226.

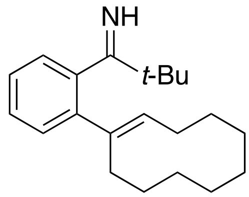

(*E*)-1-(2-(cyclodec-1-en-1-yl)phenyl)-2,2-dimethylpropan-1-imine (3ae): Light yellow oil (49 mg, 82%, *E*/*Z* > 10:1 as judged from ^{1}H NMR). R_f 0.54 (hexane/EtOAc/NEt_3 = 10/1/1); ^{1}H NMR (400 MHz, $CDCl_3$): δ 9.36 (brs, 1H), 7.26–7.23 (m, 1H), 7.21–7.17 (m, 2H), 7.05 (d, J = 7.3 Hz, 1H), 5.64 (t, J = 8.3 Hz, 1H), 2.45–2.40 (m, 2H), 2.24–2.17 (m, 2H), 1.64–1.51 (m, 8H), 1.18 (s, 9H); ^{13}C NMR (100 MHz, $CDCl_3$): δ 191.8, 141.5, 140.8 (two signals overlapped), 133.5, 130.0, 128.9, 126.5, 126.2, 40.3, 30.0, 28.3, 28.0, 27.4, 27.1, 25.5, 25.1, 21.5, 21.0; HRMS (ESI) Calcd for $C_{21}H_{32}N$ $[M + H]^+$ 298.2535, found 298.2531. The *E*-stereochemistry was assigned in light of our previous study on a related C–H alkenylation reaction.[2]

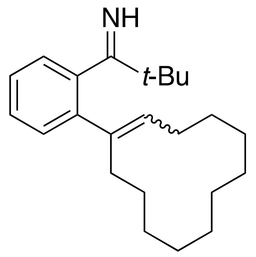

1-(2-(Cyclododec-1-en-1-yl)phenyl)-2,2-dimethylpropan-1-imine (*E*/*Z* = 4/1) (3af): Light yellow oil (51 mg, 78%). R_f 0.56 (hexane/EtOAc/NEt_3 = 10/1/1); ^{1}H NMR (400 MHz, $CDCl_3$, both isomers): δ 9.33 (brs, 1H, both isomers), 7.35–7.09 (m, 3H, both isomers), 7.04 (dd, *J* = 7.4, 1.4 Hz, 1H, major isomer), 6.97 (dd, *J* = 7.5, 0.9 Hz, 1H, minor isomer), 5.78 (t, *J* = 7.9 Hz, 1H minor isomer), 5.34 (t, *J* = 7.9 Hz, 1H major isomer), 2.46–2.16 (m, 4H, both isomers), 1.60–1.19 (m, 25H, both isomers); ^{13}C NMR (100 MHz, $CDCl_3$, major isomer): δ 191.3, 141.7, 141.4, 140.7, 133.3, 131.3, 127.8, 126.4, 126.1, 47.4, 40.3, 29.9, 27.6, 27.3, 25.7, 25.2, 24.9, 24.8, 24.7, 22.62, 22.56; HRMS (ESI) Calcd for $C_{23}H_{37}N$ $[M + H]^+$ 326.2848, found 326.2846. The E-stereochemistry of the major isomer was assigned in light of our previous study on a related C–H alkenylation reaction.[2]

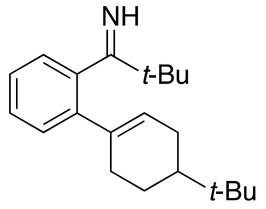

1-(4'-(*tert*-Butyl)-2',3',4',5'-tetrahydro-[1,1'-biphenyl]-2-yl)-2,2-dimethylpropan-1-imine (3ag): Light yellow oil (50 mg, 84%). R_f 0.64 (hexane/EtOAc/NEt_3 = 10/1/1); ^{1}H NMR (400 MHz, $CDCl_3$): δ 9.27 (brs, 1H), 7.28–7.23 (m, 1H), 7.20–7.15 (m, 2H), 7.01 (d, *J* = 7.2 Hz, 1H), 5.72–5.68 (m, 1H), 2.45–2.11 (m, 3H), 1.94–1.82 (m, 2H), 1.32–1.14 (m, 11H), 0.89 (s, 9H); ^{13}C NMR (100 MHz, $CDCl_3$): δ 191.9, 140.7, 140.5, 137.5, 129.7, 128.2, 127.8, 127.4, 126.1, 43.7, 40.4, 32.4, 31.9, 29.5, 27.4, 24.7; HRMS (ESI) Calcd for $C_{21}H_{32}N$ $[M + H]^+$ 298.2535, found 298.2534.

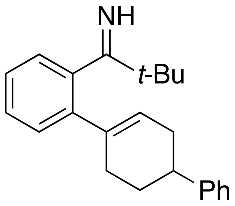

2,2-Dimethyl-1-(2',3',4',5'-tetrahydro-[1,1':4',1''-terphenyl]-2-yl)propan-1-imine (3ah): Light yellow oil (54 mg, 85%). R_f 0.48 (hexane/EtOAc/NEt_3 = 10/1/1); ^{1}H NMR (400 MHz, $CDCl_3$): δ 9.29 (brs, 1H), 7.34–7.18 (m, 8H), 7.05 (d, J = 7.3 Hz, 1H), 5.82–5.78 (m, 1H), 2.88–2.80 (m, 1H), 2.59–2.35 (m, 3H), 2.30–2.20 (m, 1H), 2.08–2.02 (m, 1H), 1.90–1.79 (m, 1H), 1.21 (s, 9H); ^{13}C NMR (100 MHz, $CDCl_3$): δ 191.8, 146.9 (two signals overlapped), 140.5, 137.7, 128.9, 128.7, 128.3, 128.0, 127.5, 127.1, 126.4, 126.3, 40.5, 39.6, 34.1, 31.1, 30.4, 29.5; HRMS (ESI) Calcd for $C_{23}H_{28}N$ $[M + H]^+$ 318.2222, found 318.2222.

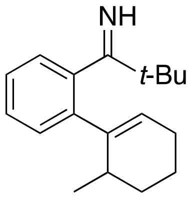

2,2-Dimethyl-1-(2'-methyl-2',3',4',5'-tetrahydro-[1,1'-biphenyl]-2-yl)propan-1-imine (3ai): Light yellow oil (45 mg, 88%). R_f 0.52 (hexane/EtOAc/NEt_3 = 10/1/1); ^{1}H NMR (400 MHz, $CDCl_3$): δ 9.34 (brs, 1H), 7.26–7.17 (m, 2H), 7.11 (d, J = 7.3 Hz, 1H), 7.03 (d, J = 7.3 Hz, 1H), 5.68–5.58 (m, 1H), 2.58–2.42 (m, 1H), 2.22–2.06 (m, 2H), 1.89–1.64 (m, 2H), 1.44–1.19 (m, 11H), 0.84 (d, J = 7.0 Hz, 3H); ^{13}C NMR (100 MHz, $CDCl_3$): δ 191.6, 144.1, 140.8, 140.5, 130.6, 128.7, 127.7, 126.4, 126.0, 40.3, 33.0, 31.5, 30.0, 26.2, 20.3, 19.8; HRMS (ESI) Calcd for $C_{18}H_{26}NO$ $[M + H]^+$ 256.2065, found 256.2061.

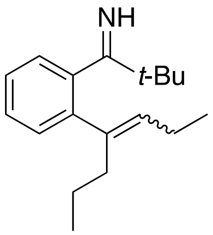

1-(2-(Hept-3-en-4-yl)phenyl)-2,2-dimethylpropan-1-imine (*E*/*Z* = 4/1) (3aj): Light yellow oil (46 mg, 90%). R_f 0.52 (hexane/EtOAc/NEt_3 = 10/1/1); ^{1}H NMR (400 MHz, $CDCl_3$, major isomer): δ 9.30 (brs, 1H), 7.26–7.13 (m, 3H), 7.05–7.02 (m, 1H), 5.37 (t, *J* = 7.2 Hz, 1H), 2.29–2.24 (m, 2H), 2.14 (p, *J* = 7.5 Hz, 2H), 1.30 (q, *J* = 7.5 Hz, 2H), 1.21 (s, 9H), 1.01 (t, *J* = 7.6 Hz, 3H), 0.86 (t, *J* = 7.1 Hz, 3H); ^{13}C NMR (100 MHz, $CDCl_3$, major isomer): δ 191.4, 141.6, 140.7, 140.5, 134.4, 130.5, 127.8, 126.6, 126.1, 40.3, 34.2, 29.9, 22.1, 21.8, 14.4, 14.2; HRMS (ESI) Calcd for $C_{18}H_{28}N$ $[M + H]^+$ 258.2222, found 258.2224. The *E*-stereochemistry of the major isomer was assigned in light of our previous study on a related C–H alkenylation reaction.[2]

Cyclization of alkenylated N–H imine under peroxide photolysis

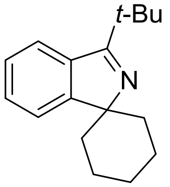

3'-(*tert*-Butyl)spiro[cyclohexane-1,1'-isoindole] (4): 2,2-Dimethyl-1-(2',3',4',5'-tetrahydro-[1,1'-biphenyl]-2-yl)propan-1-imine (**3aa**, 48 mg, 0.20 mmol) was weighed into a 10 mL vial containing a stirring bar and then dissolved in *t*-BuOO*t*-Bu (0.5 mL). The vial was placed in Luzchem LZC-4V photoreactor and irradiated at 254 nm for 12 h. Then the mixture was concentrated under reduced pressure, and the residue was subjected to silica gel chromatography (eluent: hexane/EtOAc 100:1) to afford the title product as a yellow oil (39 mg, 81%).

R_f 0.41 (hexane/EtOAc = 10/1); ^{1}H NMR (400 MHz, $CDCl_3$): δ 7.72–7.69 (m, 1H), 7.55–7.51 (m, 1H), 7.33–7.29 (m, 2H), 2.05–1.93 (m, 2H), 1.82–1.72 (m, 2H), 1.71–1.56 (m, 4H), 1.49–1.40 (m, 11H); ^{13}C NMR (100 MHz, $CDCl_3$): δ 175.4, 160.8, 137.1, 127.2, 126.7, 123.9, 122.0, 75.6, 36.3, 35.5, 28.9, 26.2, 23.6; HRMS (ESI) Calcd for $C_{17}H_{24}N$ $[M + H]^+$ 242.1909, found 242.1912.

References

(1) Bernardi, T.; Badel, S.; Mayer, P.; Groelly, J.; De Fremont, P.; Jacques, B.; Braunstein, P.; Teyssot, M.-L.; Gaulier, C.; Cisnetti, F.; Gautier, A.; Roland, S. *ChemMedChem* **2014**, *9*, 1140.

(2) Lee, P.-S.; Xu, W.; Yoshikai, N. *Adv. Synth. Catal.* **2017**, *359*, 4340.

(3) Grieco, G.; Blacque, O.; Berke, H. *Beilstein J. Org. Chem.* **2015**, *11*, 1656.

(4) He, R.; Huang, Z.-T.; Zheng, Q.-Y.; Wang, C. *Angew. Chem. Int. Ed.* **2014**, *53*, 4950.

(5) Port, A.; Sanchez-Aris, M.; Cervelló, E.; Jaime, C.; Virgili, A.; Farriol, M.; Gallardo, I. *Polycyclic Aromat. Compd.* **2003**, *23*, 457.

(6) Xu, W. G.; Yoshikai, N. *Chem. Sci.* **2017**, *8*, 5299.

(7) Hayashi, T.; Fujiwa, T.; Okamoto, Y.; Katsuro, Y.; Kumada, M. *Synthesis* **1981**, *12*, 1001.

(8) Emmett, E. J.; Hayter, R. B.; Willis, C. M. *Angew. Chem. Int. Ed.* **2014**, *53*, 10204.

NMR Spectra

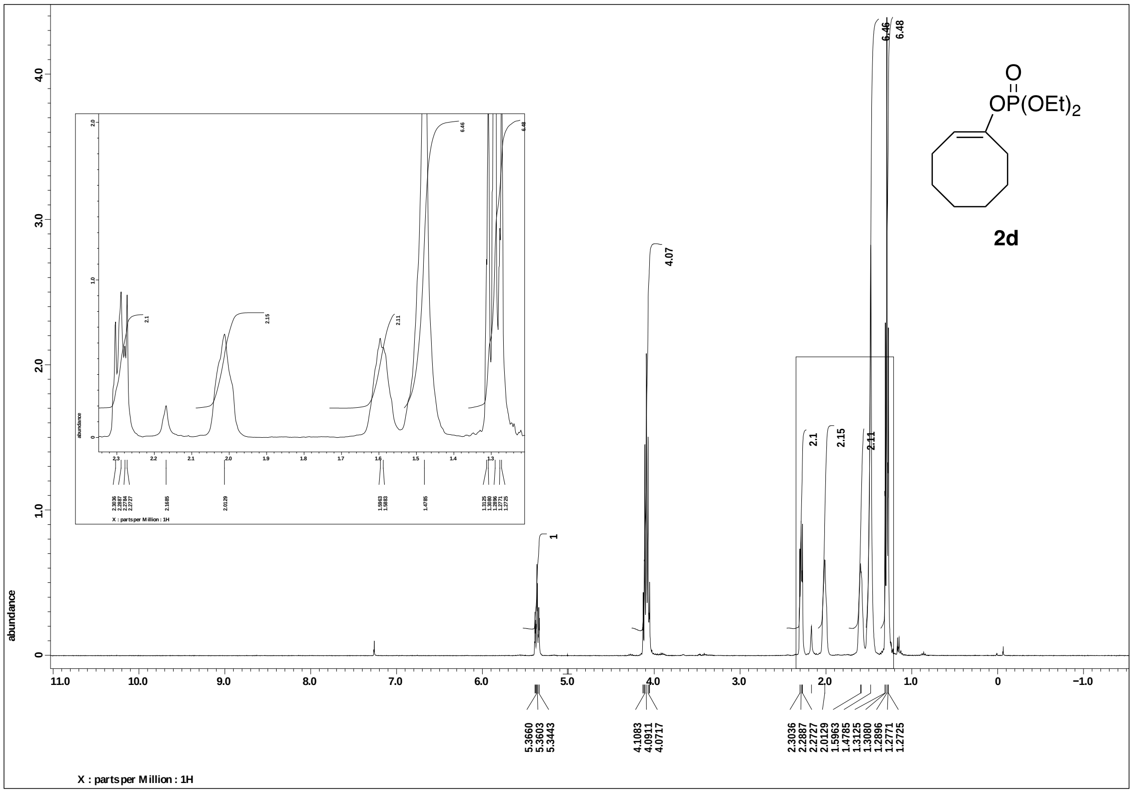
OP(OEt)2
2d
4.07
1
2.1
2.15
2.11
6.46
6.48
5.3660
5.3603
5.3443
4.1083
4.0911
4.0717
2.3036
2.2887
2.2727
2.0129
1.5963
1.4785
1.3125
1.3080
1.2896
1.2771
1.2725
X : parts per Million : 1H
abundance

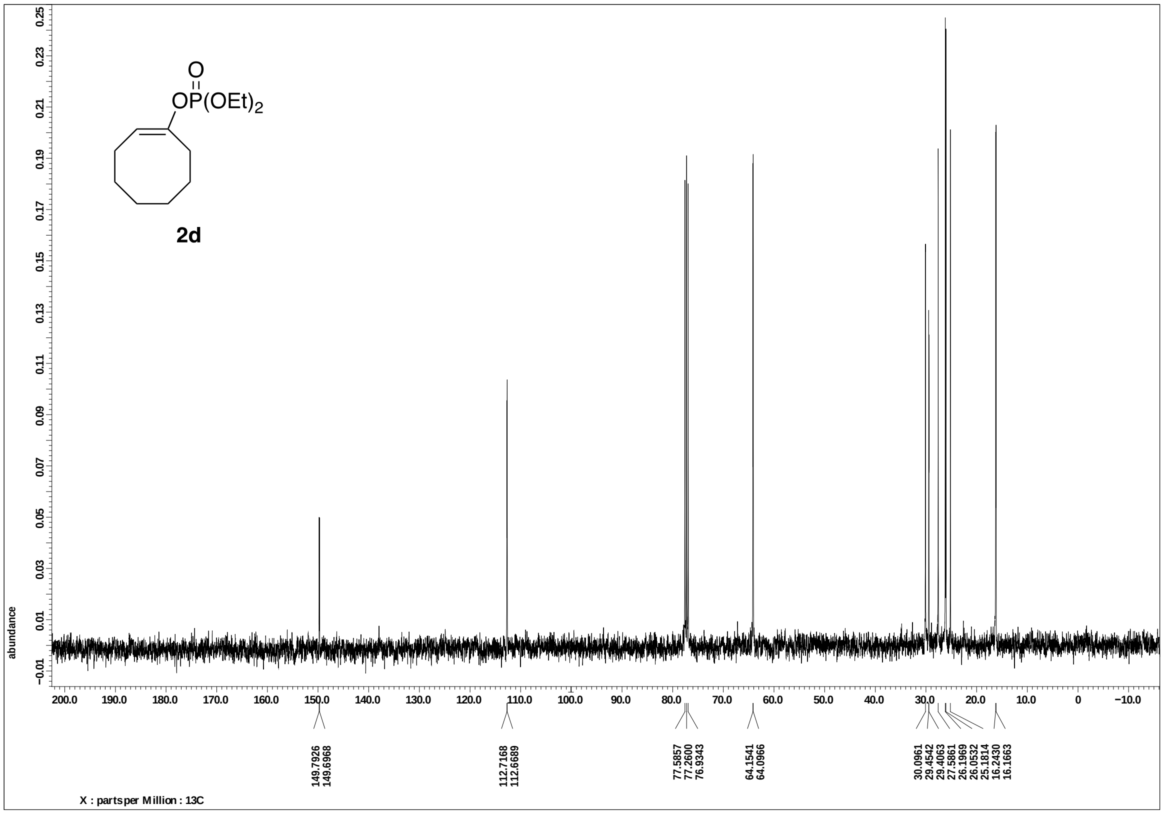
OP(OEt)2
2d
149.7926
149.6968
112.7168
112.6689
77.5857
77.2600
76.9343
64.1541
64.0966
30.0961
29.4542
29.4063
27.5861
26.1909
26.0532
25.1814
16.2430
16.1663
X : parts per Million : 13C
abundance

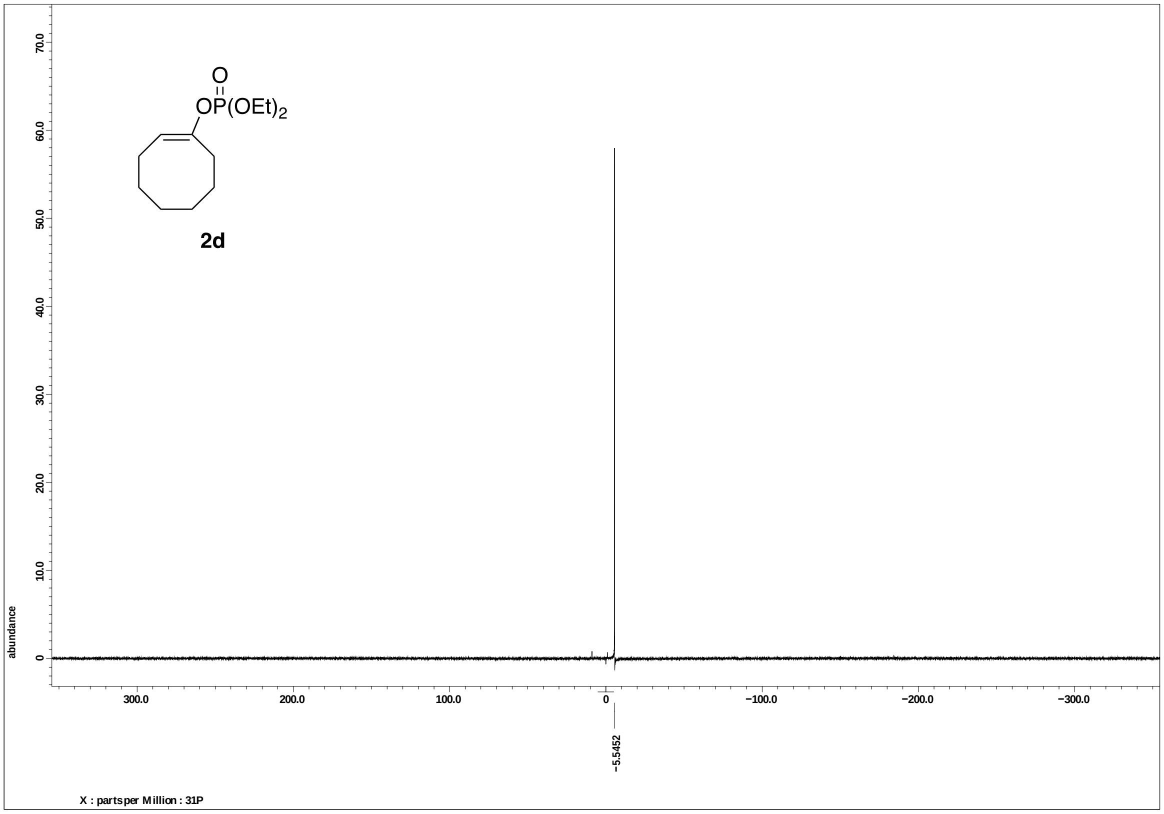
OP(OEt)2
O
2d
abundance
X : partsper Million : 31P
-5.5452
300.0
200.0
100.0
0
−100.0
−200.0
−300.0

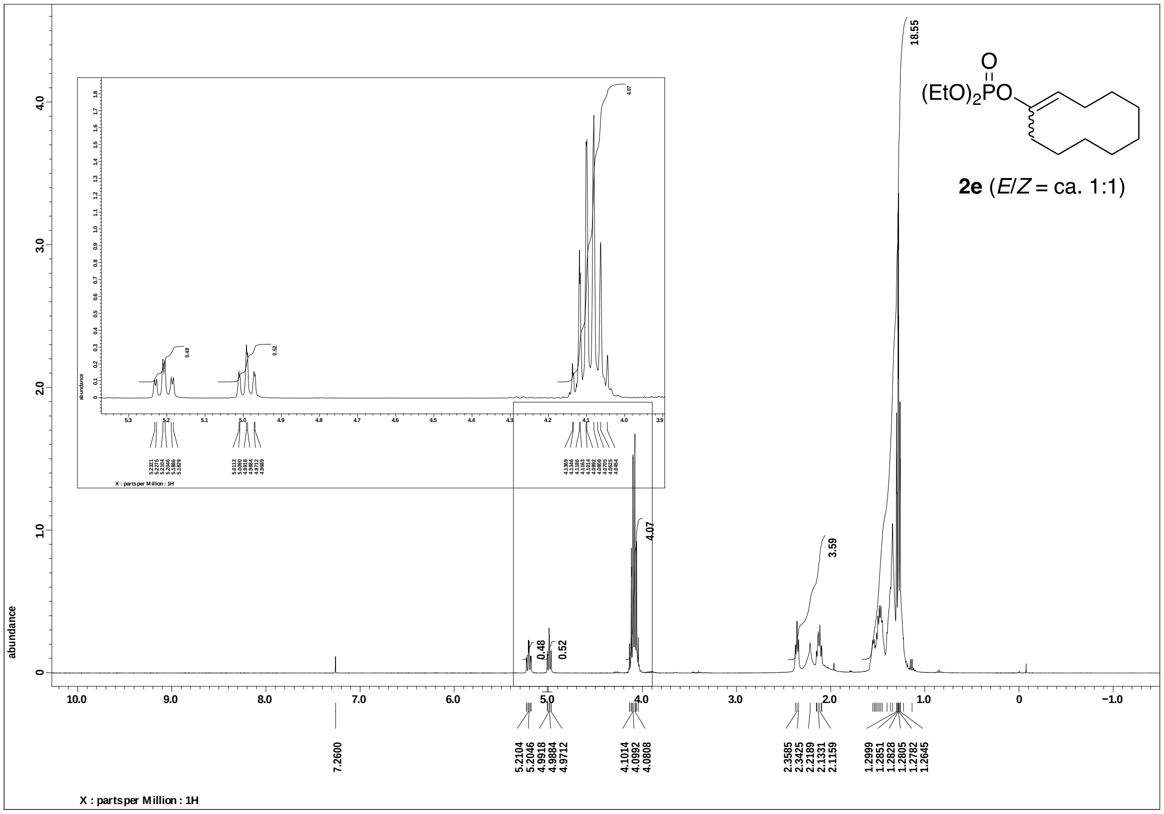

(EtO)2PO
2e (E/Z = ca. 1:1)
abundance
18.55
4.07
3.59
0.48
0.52
7.2600
5.2104
5.2046
4.9918
4.9884
4.9712
4.1014
4.0992
4.0808
2.3585
2.3425
2.2189
2.1331
2.1159
1.2999
1.2851
1.2828
1.2805
1.2782
1.2645
X : parts per Million : 1H

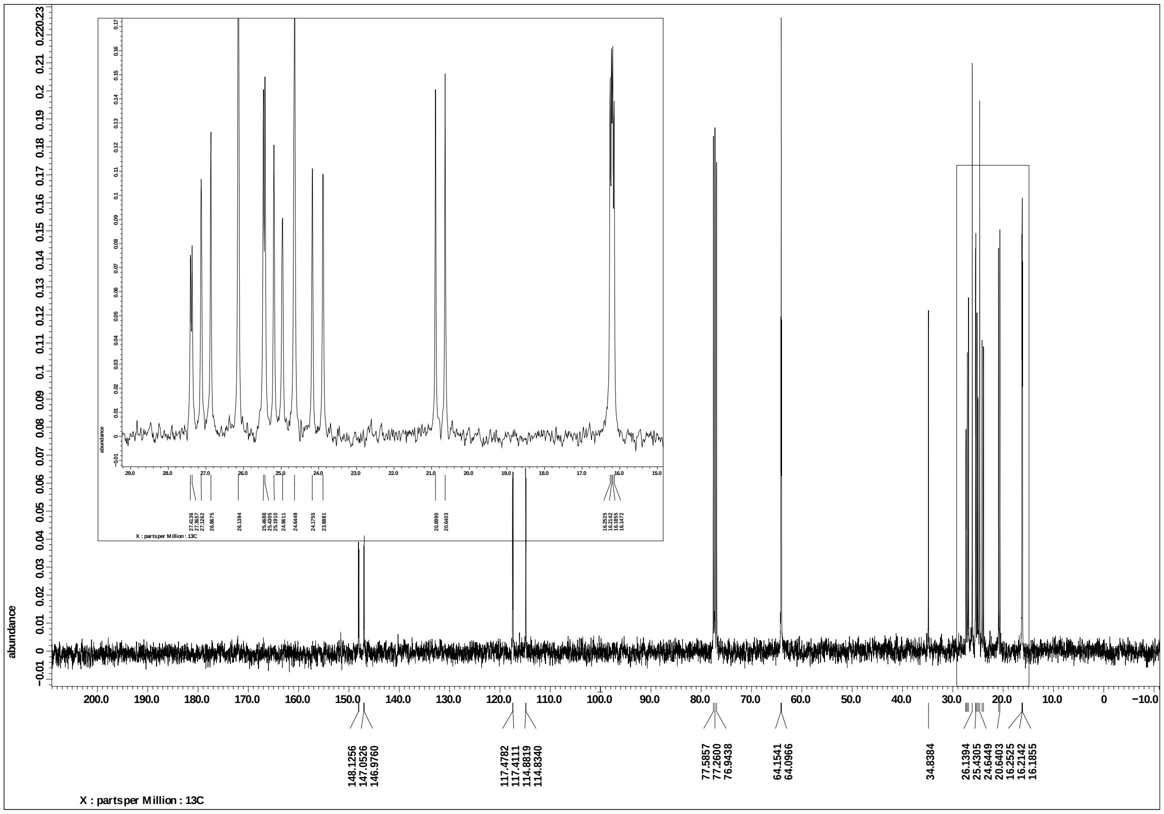

abundance
148.1256
147.0526
146.9760
117.4782
117.4111
114.8819
114.8340
77.5857
77.2600
76.9438
64.1541
64.0966
34.8384
26.1394
25.4305
24.6449
20.6403
16.2525
16.2142
16.1855
X : parts per Million : 13C

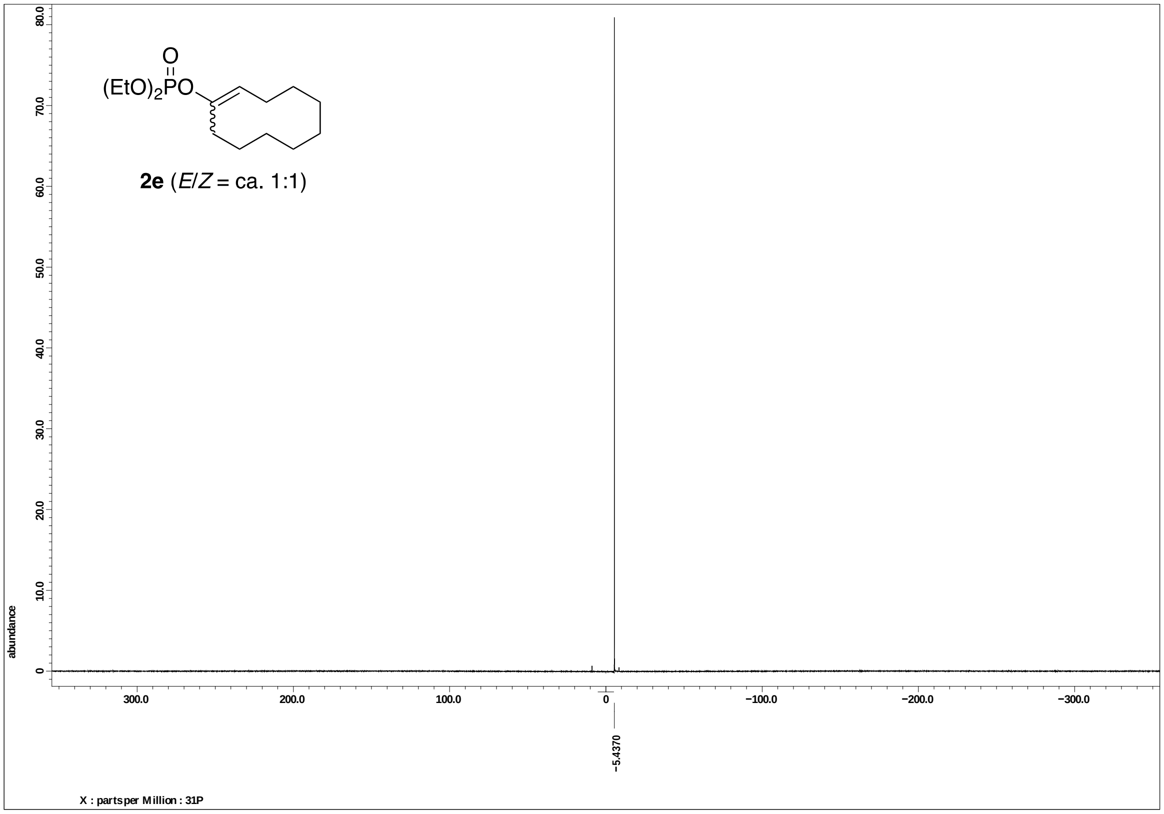

(EtO)2PO
O
2e (E/Z = ca. 1:1)
abundance
80.0
70.0
60.0
50.0
40.0
30.0
20.0
10.0
0
300.0
200.0
100.0
0
−100.0
−200.0
−300.0
−5.4370
X : parts per Million : 31P

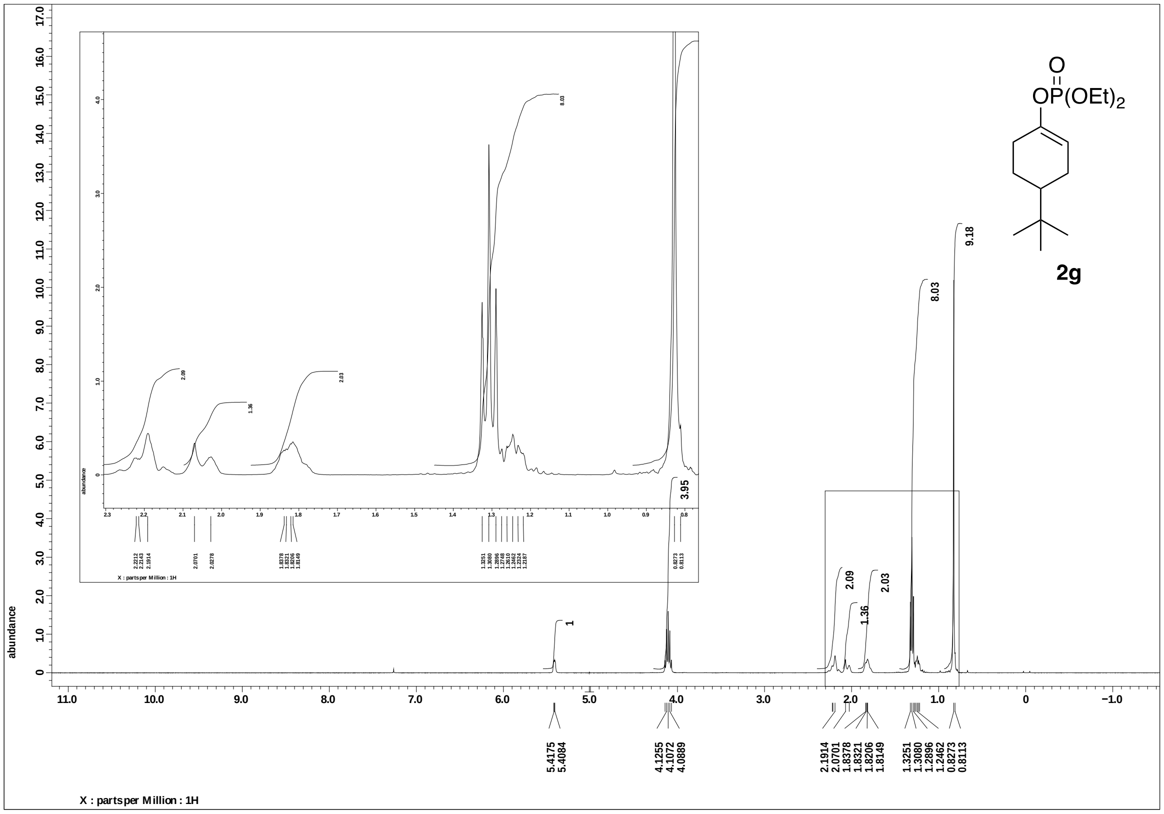

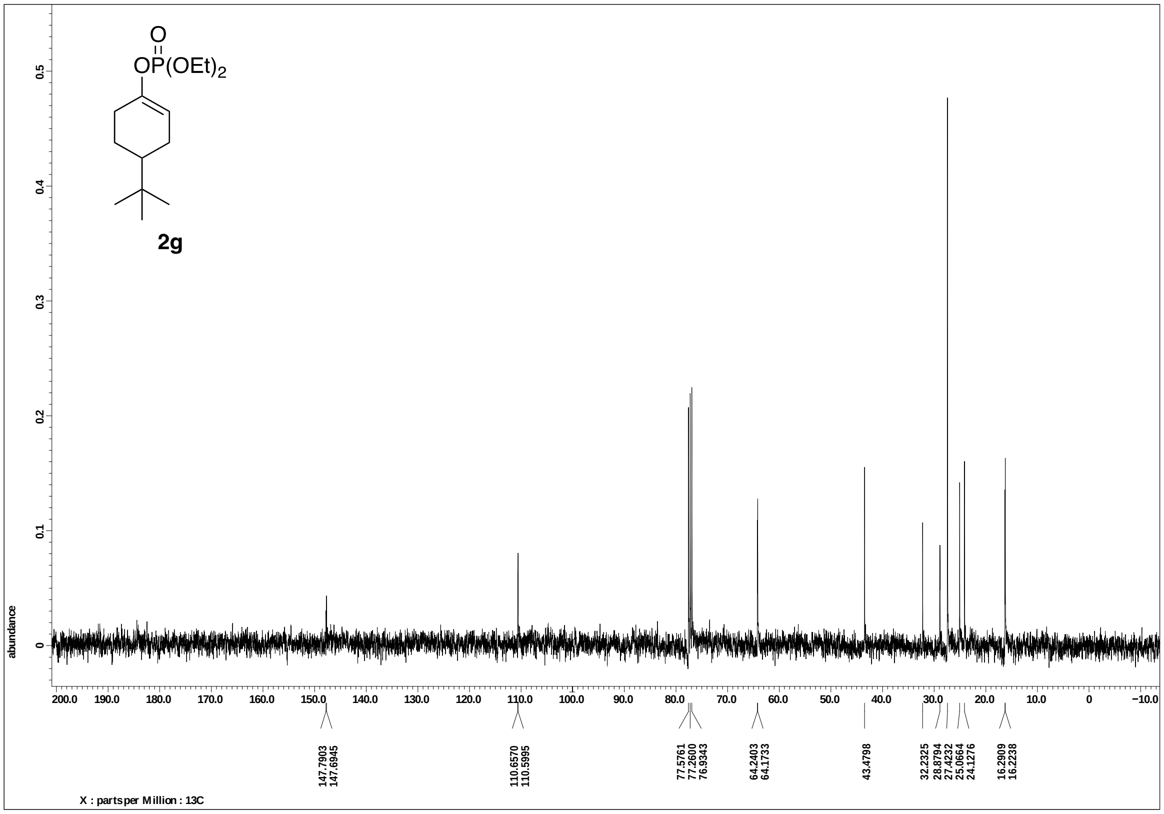

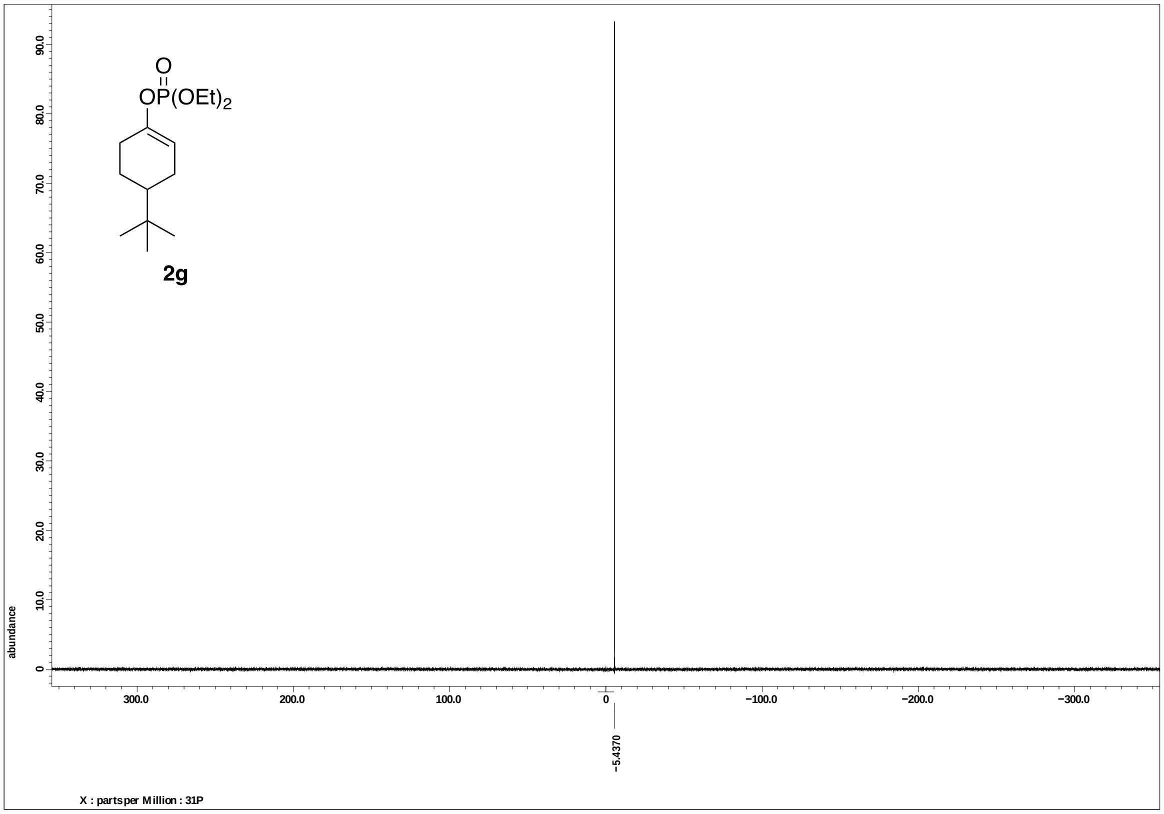
OP(OEt)2
O
2g
abundance
90.0
80.0
70.0
60.0
50.0
40.0
30.0
20.0
10.0
0
300.0
200.0
100.0
0
−100.0
−200.0
−300.0
−5.4370
X : parts per Million : 31P

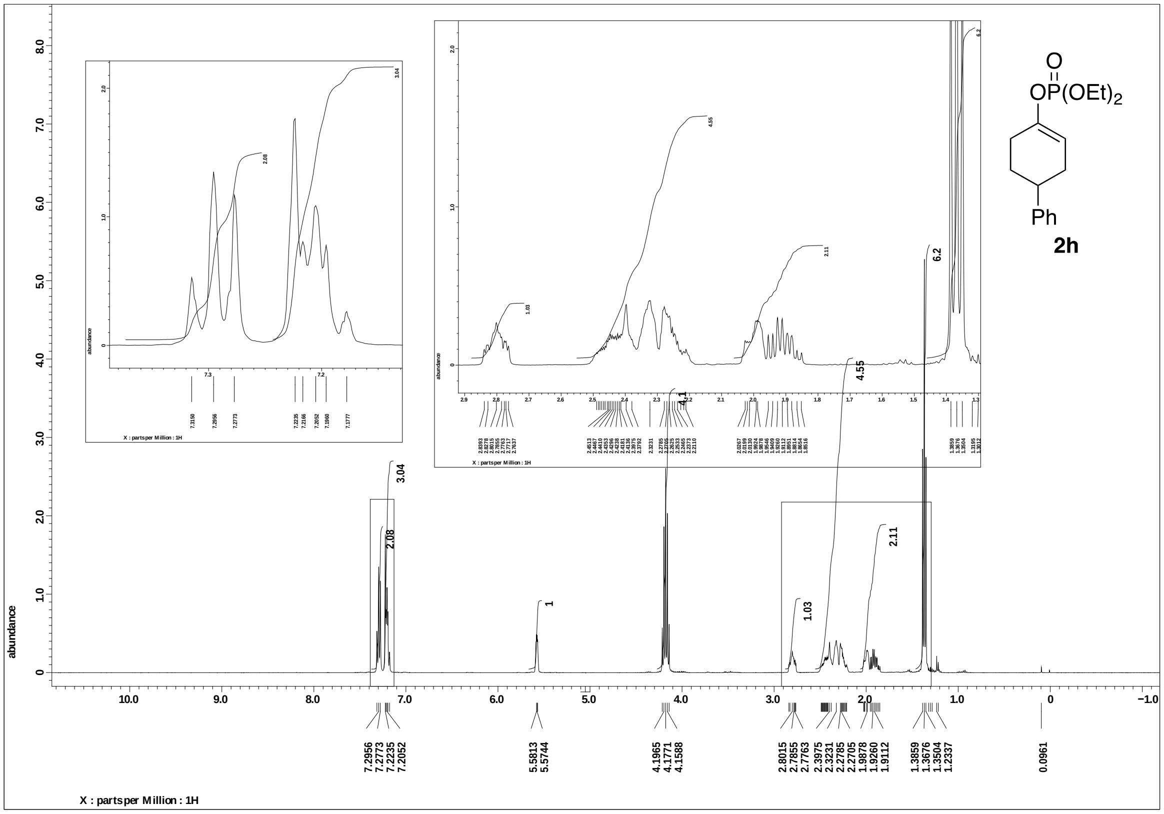
O
OP(OEt)2
Ph
2h
abundance
X : partsper Million : 1H
3.04
2.08
1
1.03
4.55
2.11
6.2
7.2956
7.2773
7.2235
7.2052
5.5813
5.5744
4.1965
4.1771
4.1588
2.8015
2.7855
2.7763
2.3975
2.3231
2.2785
2.2705
1.9878
1.9260
1.9112
1.3859
1.3676
1.3504
1.2337
0.0961

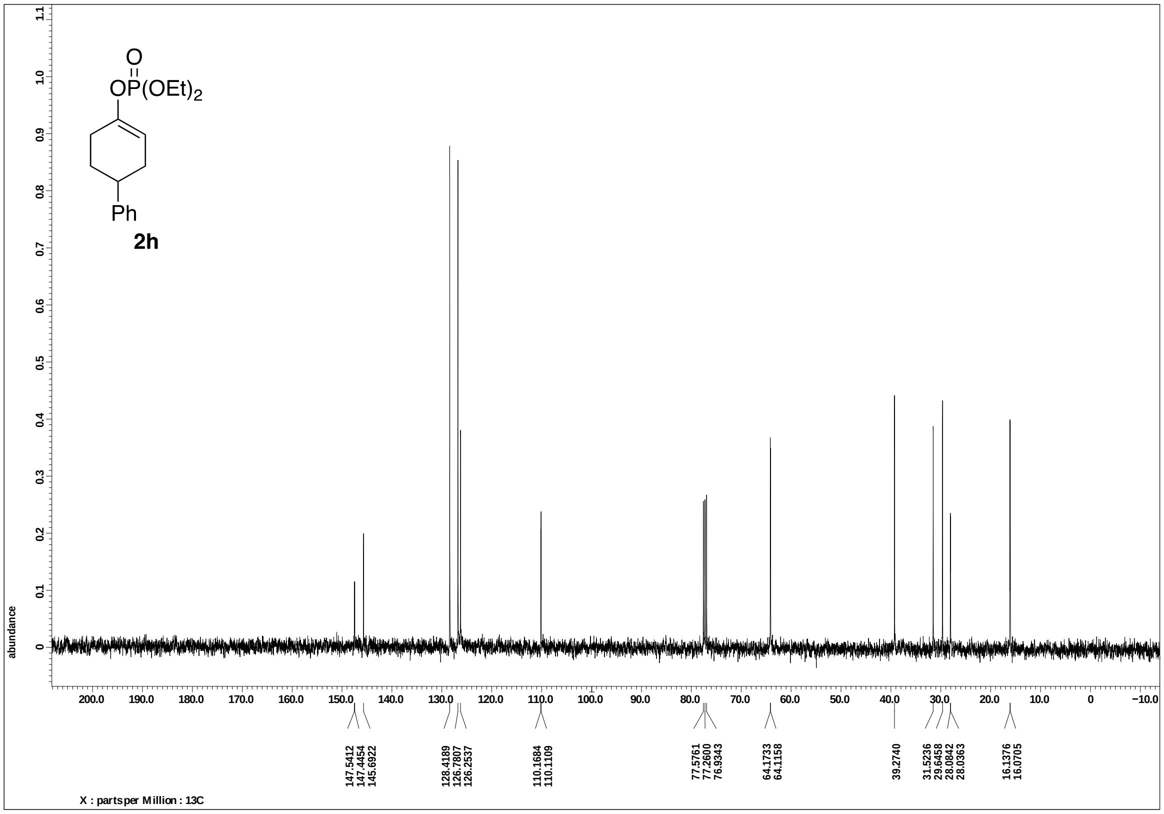
O
OP(OEt)2
Ph
2h
abundance
X : partsper Million : 13C
147.5412
147.4454
145.6922
128.4189
126.7807
126.2537
110.1684
110.1109
77.5761
77.2600
76.9343
64.1733
64.1158
39.2740
31.5236
29.6458
28.0842
28.0363
16.1376
16.0705

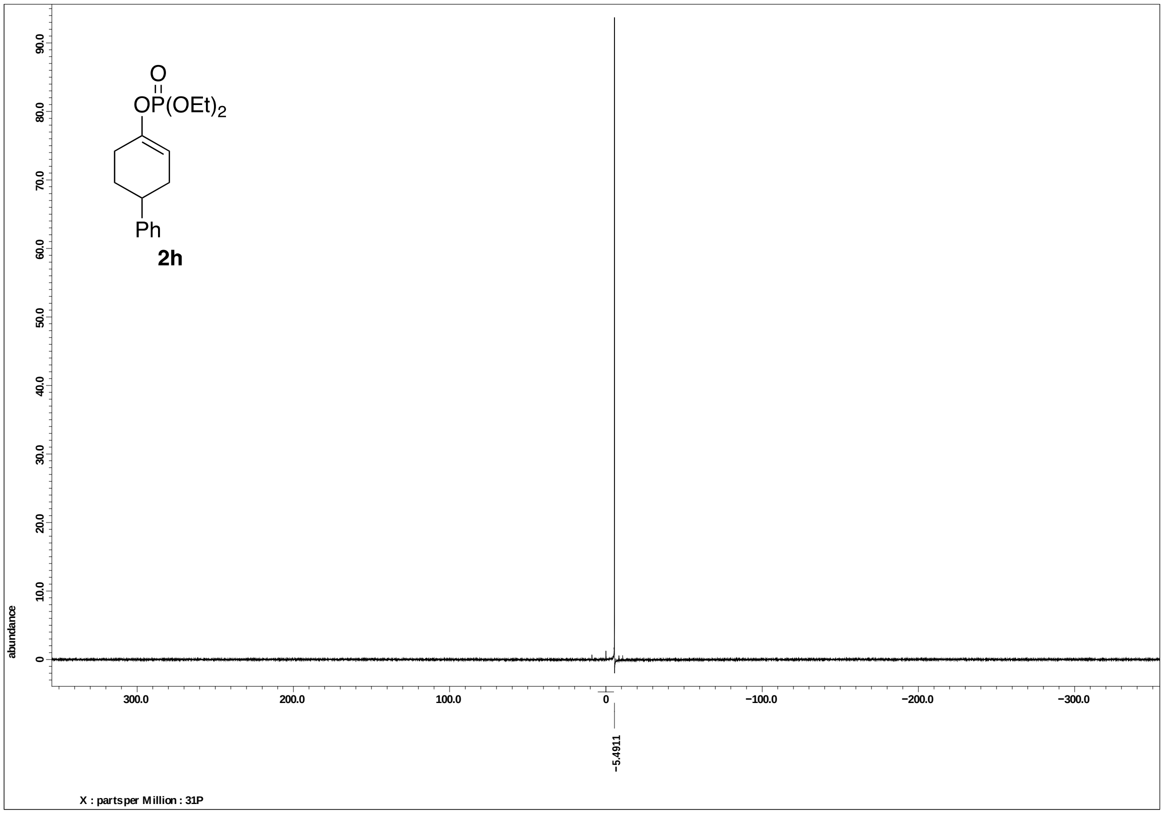
O
OP(OEt)2
Ph
2h
abundance
90.0
80.0
70.0
60.0
50.0
40.0
30.0
20.0
10.0
0
300.0
200.0
100.0
0
−100.0
−200.0
−300.0
−5.4911
X : partsper Million : 31P

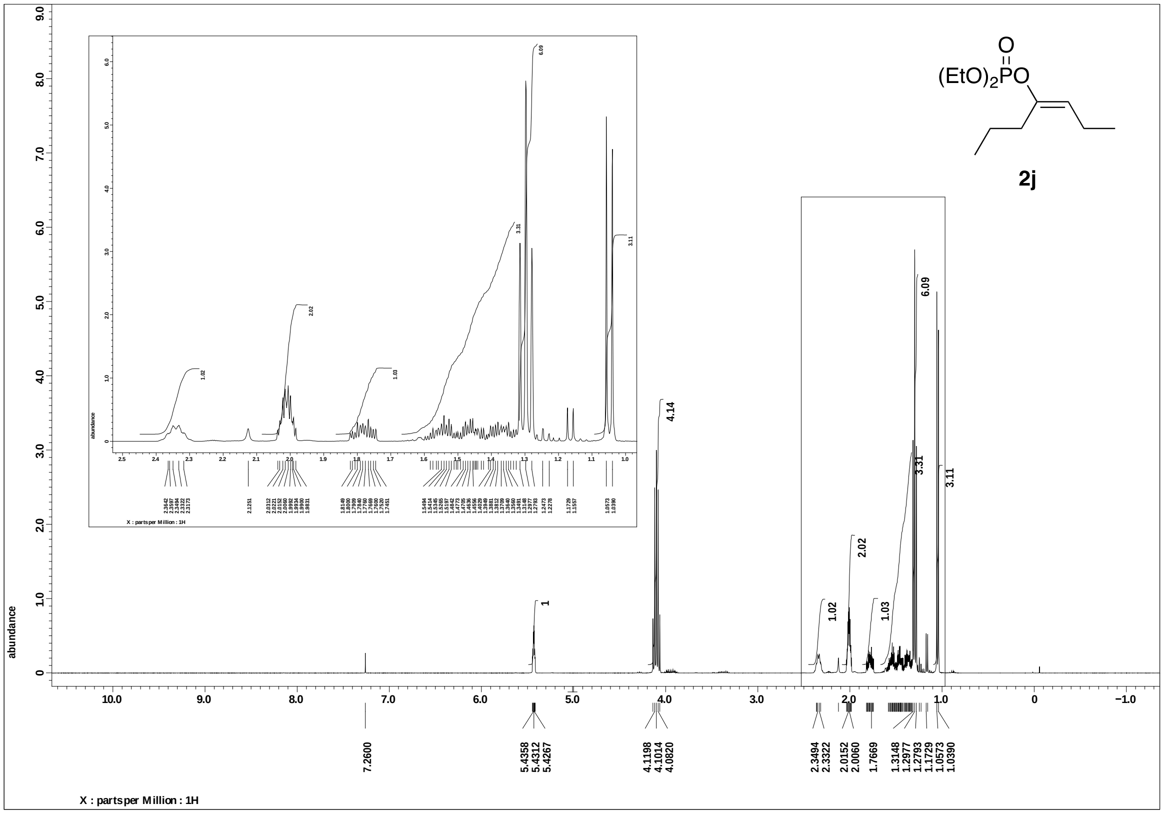

(EtO)2PO
O
2j
abundance
X : partsper Million : 1H
7.2600
5.4358
5.4312
5.4267
4.1198
4.1014
4.0820
2.3494
2.3322
2.0152
2.0060
1.7669
1.3148
1.2977
1.2793
1.1729
1.0573
1.0390
1
4.14
1.02
2.02
1.03
3.31
6.09
3.11

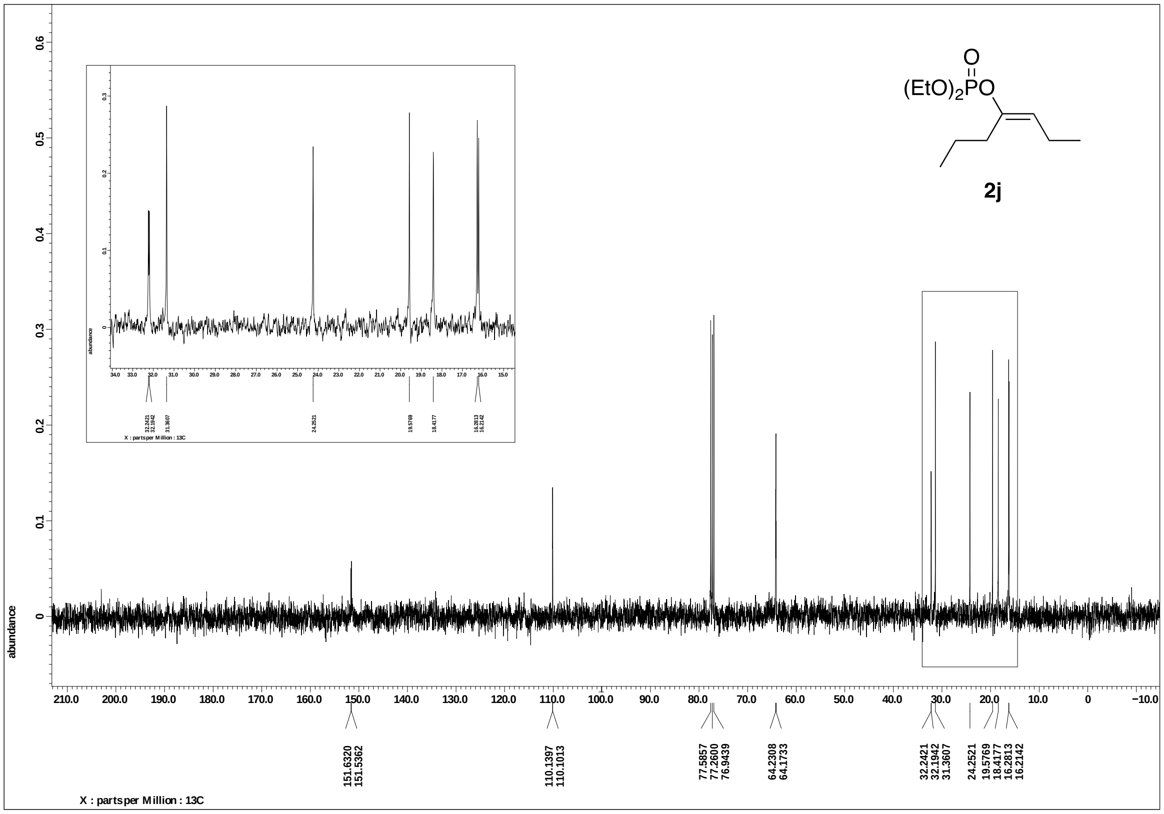

(EtO)2PO
O
2j
abundance
X : partsper Million : 13C
151.6320
151.5362
110.1397
110.1013
77.5857
77.2600
76.9439
64.2308
64.1733
32.2421
32.1942
31.3607
24.2521
19.5769
18.4177
16.2813
16.2142

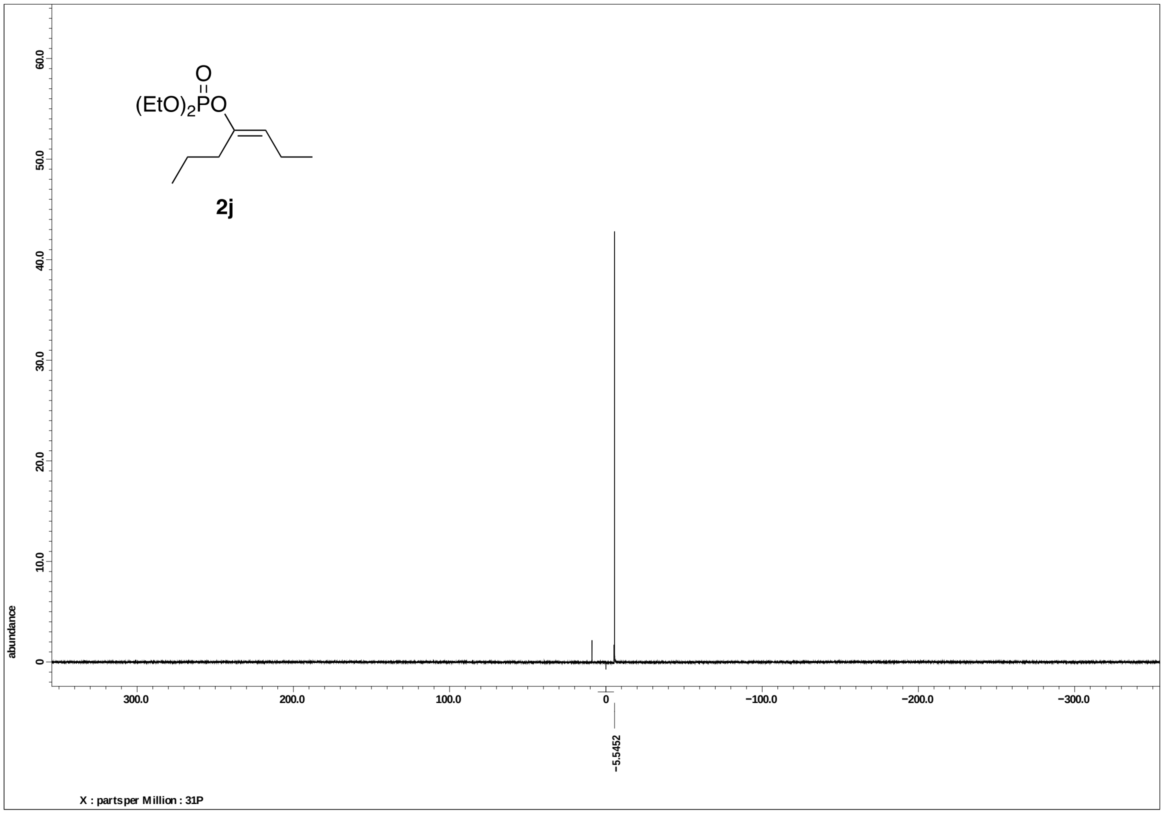
(EtO)2PO
O
2j
abundance
60.0
50.0
40.0
30.0
20.0
10.0
0
300.0
200.0
100.0
0
−100.0
−200.0
−300.0
−5.5452
X : parts per Million : 31P

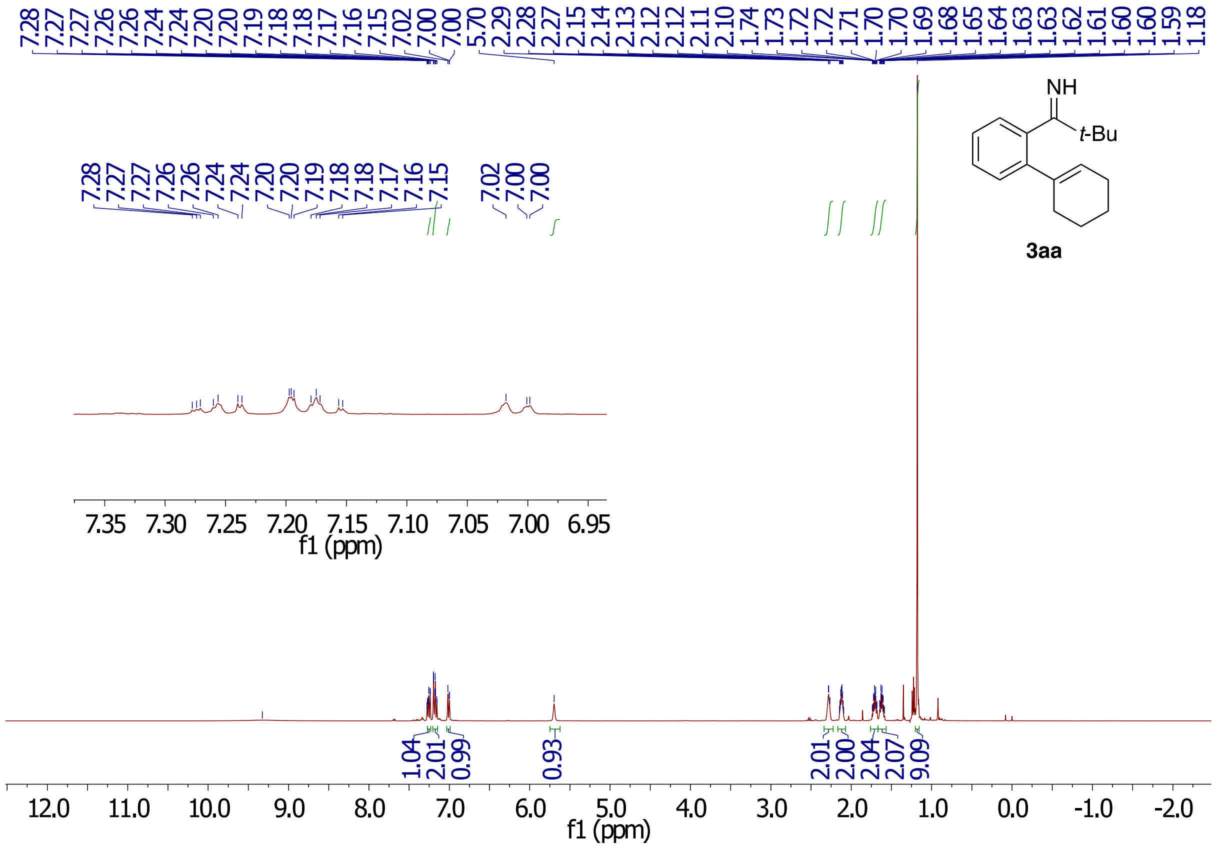
7.28 7.27 7.27 7.26 7.26 7.24 7.24 7.20 7.20 7.19 7.18 7.18 7.17 7.16 7.15 7.02 7.00 7.00 5.70 2.29 2.28 2.27 2.15 2.14 2.13 2.12 2.12 2.11 2.10 1.74 1.73 1.72 1.72 1.71 1.70 1.70 1.69 1.68 1.65 1.64 1.63 1.63 1.62 1.61 1.60 1.60 1.59 1.18
NH
t-Bu
3aa
7.35 7.30 7.25 7.20 7.15 7.10 7.05 7.00 6.95
f1 (ppm)
1.04 2.01 0.99 0.93 2.01 2.00 2.04 2.07 9.09
12.0 11.0 10.0 9.0 8.0 7.0 6.0 5.0 4.0 3.0 2.0 1.0 0.0 -1.0 -2.0
f1 (ppm)

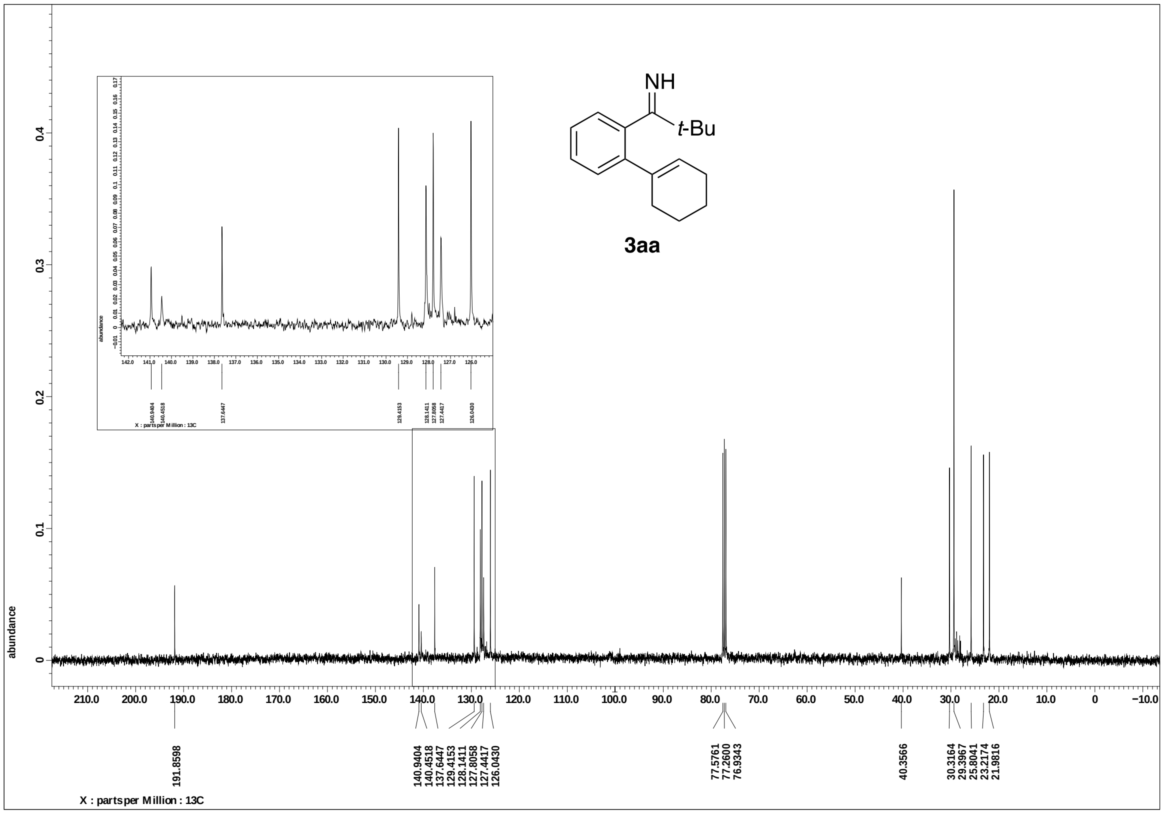
NH
t-Bu
3aa
abundance
X : parts per Million : 13C
191.8598
140.9404 140.4518 137.6447 129.4153 128.1411 127.8058 127.4417 126.0430
77.5761 77.2600 76.9343
40.3566
30.3164 29.3967 25.8041 23.2174 21.9816

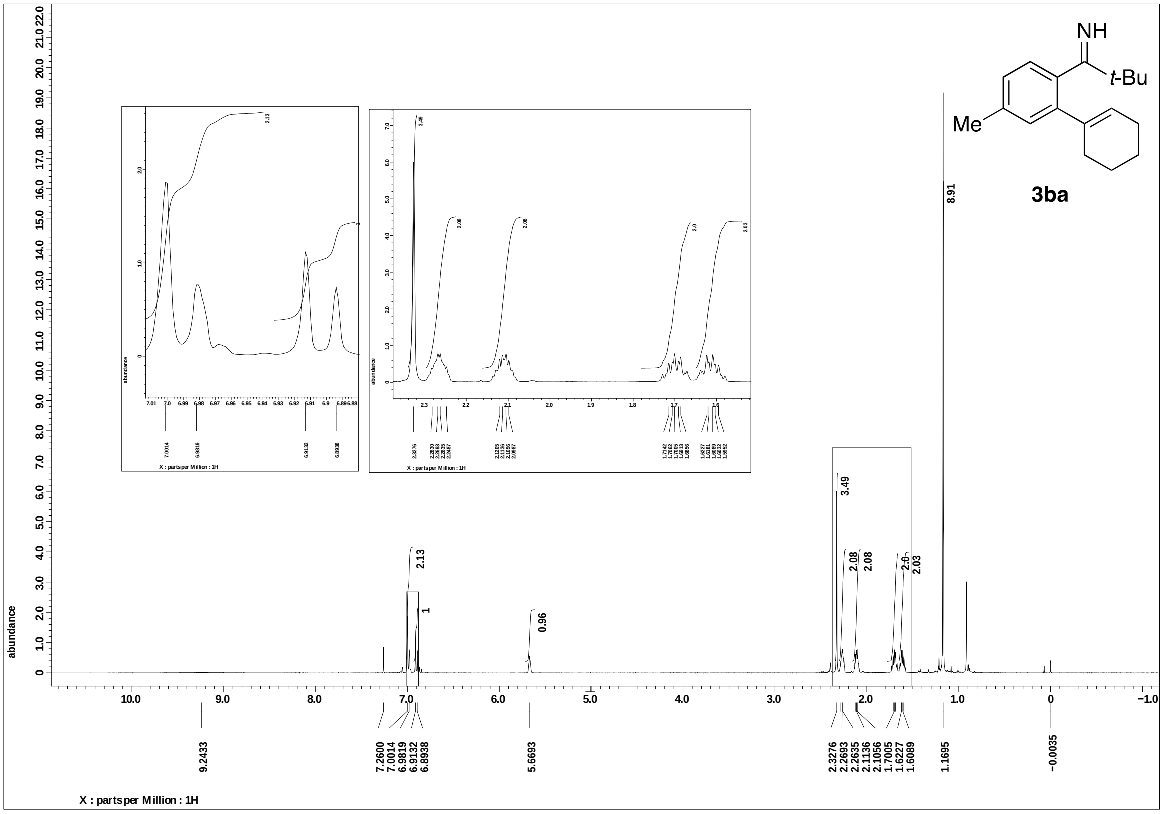

NH
t-Bu
Me
3ba
abundance
X : parts per Million : 1H
8.91
3.49
2.08
2.08
2.0
2.03
2.13
1
0.96
9.2433
7.2600
7.0014
6.9819
6.9132
6.8938
5.6693
2.3276
2.2693
2.2635
2.1136
2.1056
1.7005
1.6227
1.6089
1.1695
−0.0035

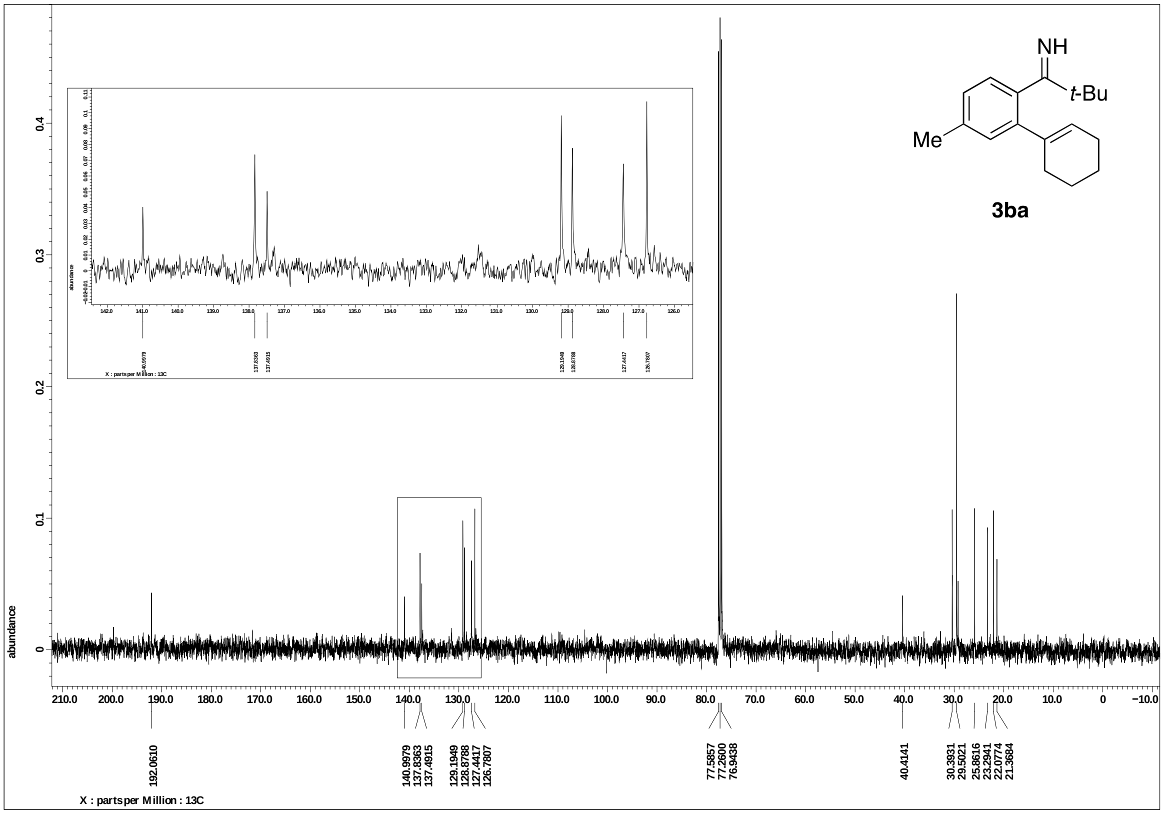

NH
t-Bu
Me
3ba
abundance
X : parts per Million : 13C
192.0610
140.9979
137.8363
137.4915
129.1949
128.8788
127.4417
126.7807
77.5857
77.2600
76.9438
40.4141
30.3931
29.5021
25.8616
23.2941
22.0774
21.3684

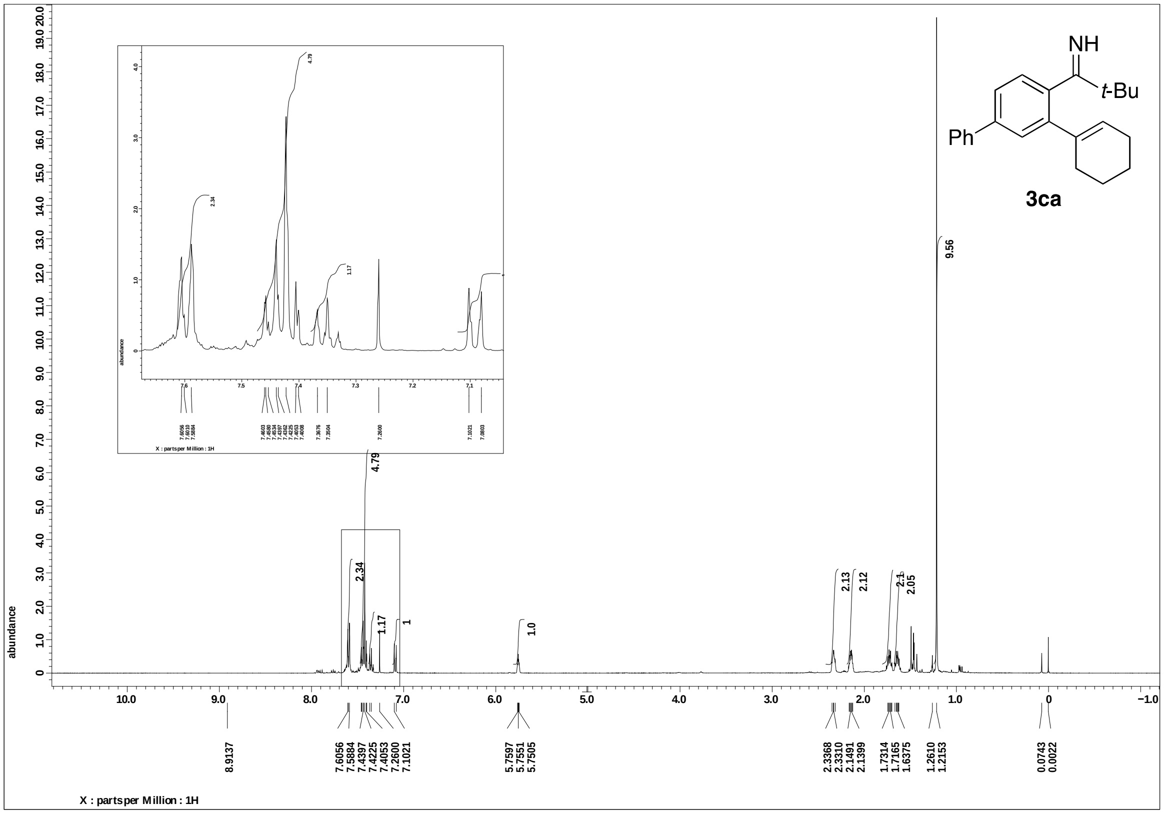
NH
t-Bu
Ph
3ca
abundance
X : partsper Million : 1H
8.9137
7.6056
7.5884
7.4397
7.4225
7.4053
7.2600
7.1021
5.7597
5.7551
5.7505
2.3368
2.3310
2.1491
2.1399
1.7314
1.7165
1.6375
1.2610
1.2153
0.0743
0.0022
4.79
2.34
1.17
1
1.0
2.13
2.12
2.1
2.05
9.56

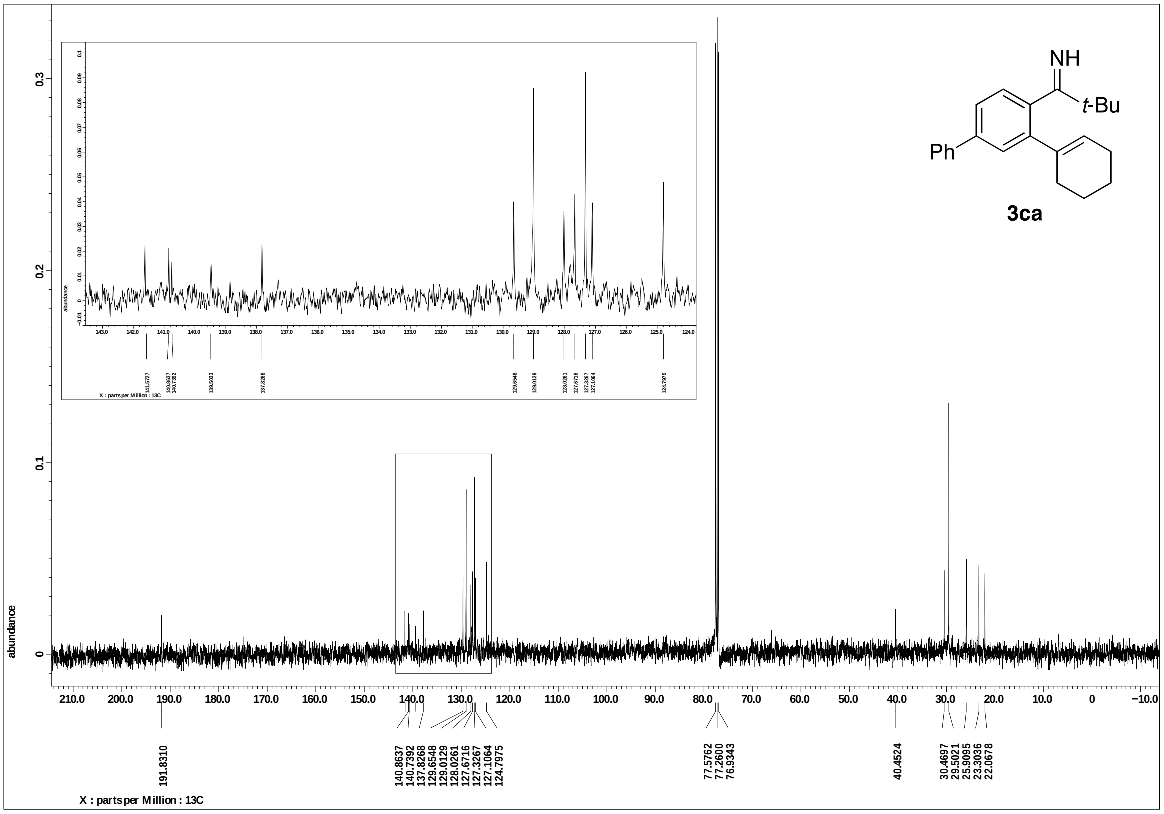
NH
t-Bu
Ph
3ca
abundance
X : partsper Million : 13C
191.8310
140.8637
140.7392
137.8268
129.6548
129.0129
128.0261
127.6716
127.3267
127.1064
124.7975
77.5762
77.2600
76.9343
40.4524
30.4697
29.5021
25.9095
23.3036
22.0678

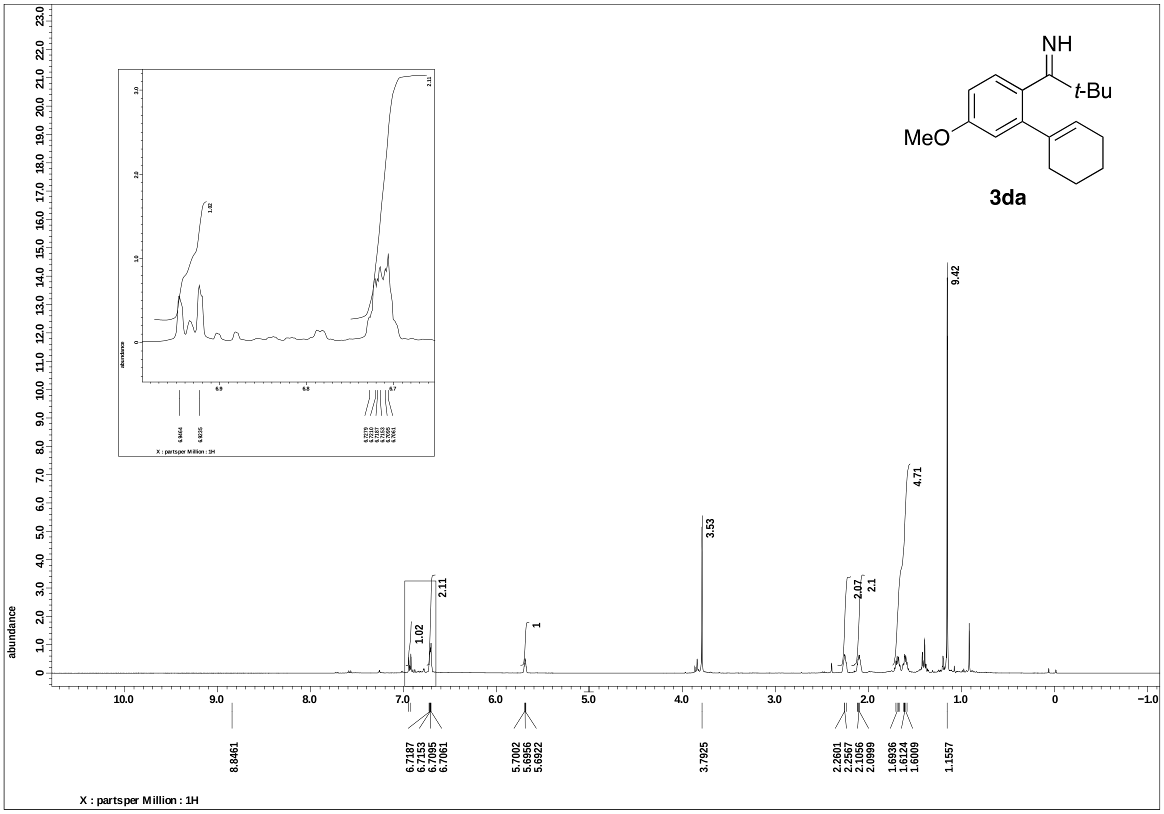
NH
t-Bu
MeO
3da
abundance
X : partsper Million : 1H
1.02
2.11
1
3.53
2.07
2.1
4.71
9.42
8.8461
6.7187
6.7153
6.7095
6.7061
5.7002
5.6956
5.6922
3.7925
2.2601
2.2567
2.1056
2.0999
1.6936
1.6124
1.6009
1.1557

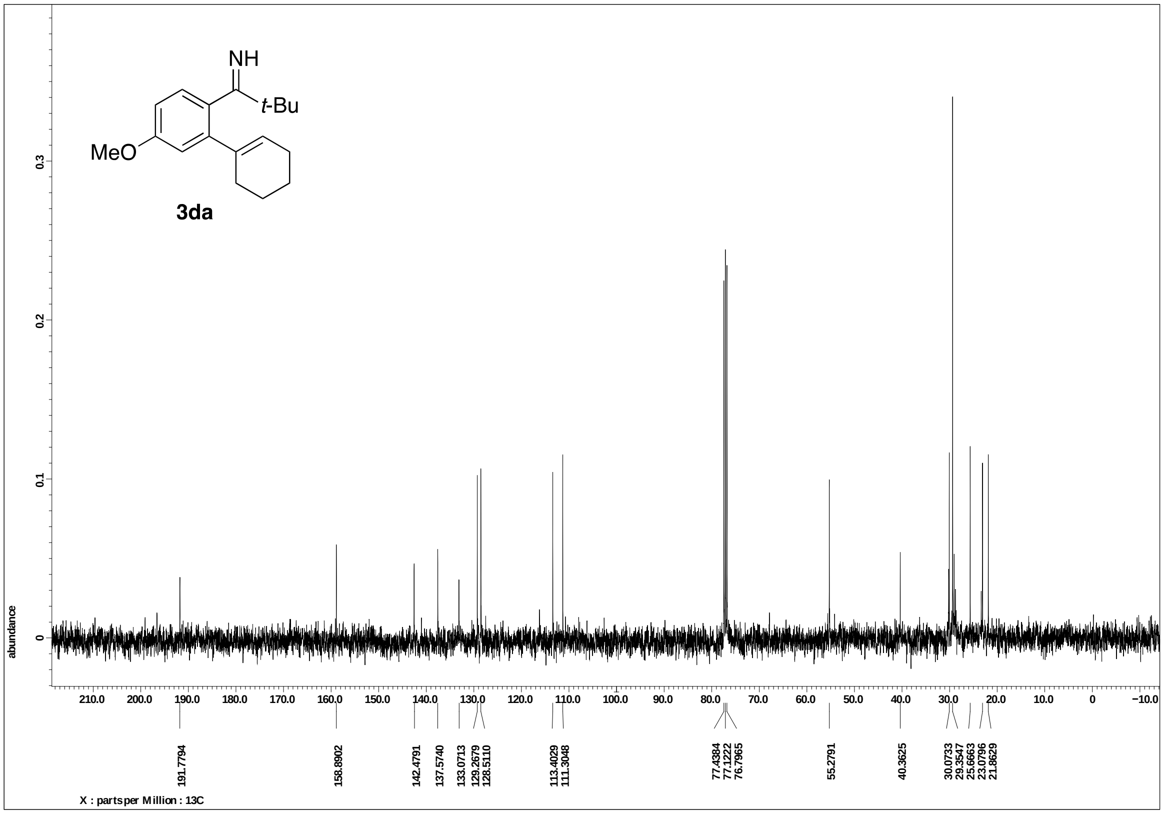
NH
t-Bu
MeO
3da
abundance
X : partsper Million : 13C
191.7794
158.8902
142.4791
137.5740
133.0713
129.2679
128.5110
113.4029
111.3048
77.4384
77.1222
76.7965
55.2791
40.3625
30.0733
29.3547
25.6663
23.0796
21.8629

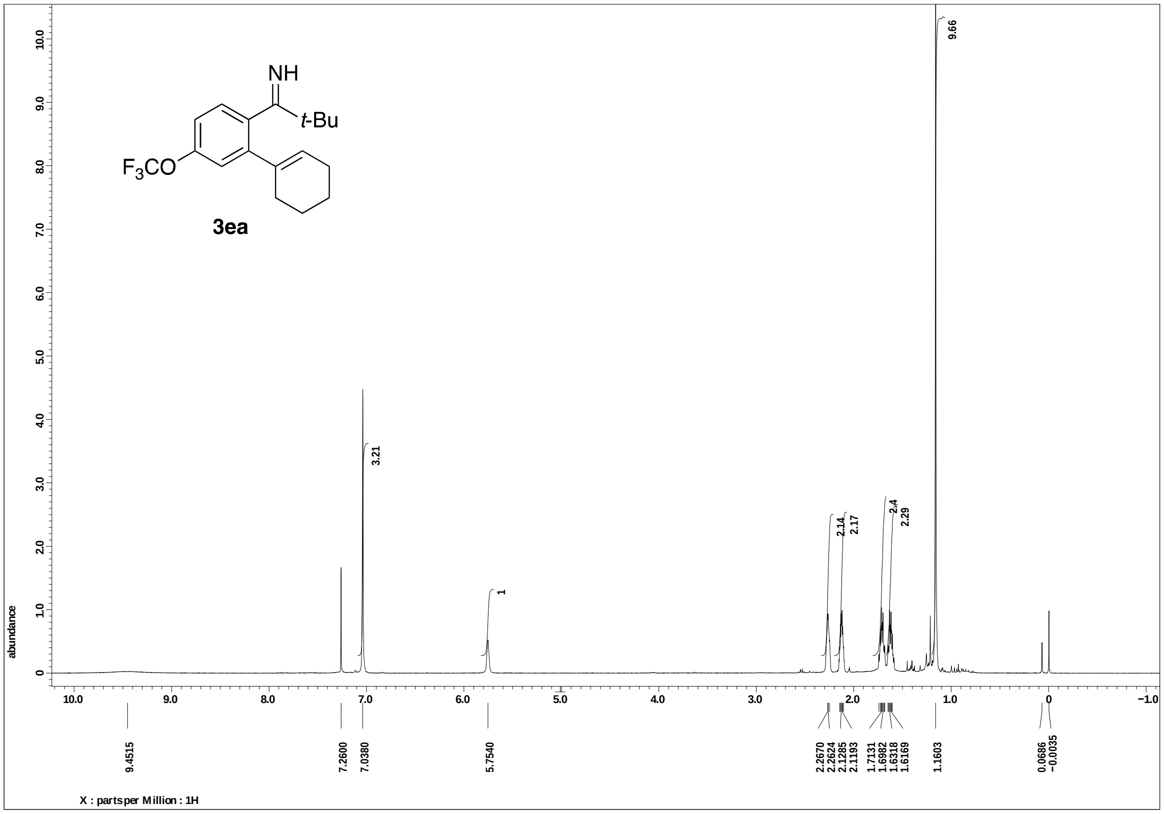
NH
t-Bu
F3CO
3ea
abundance
X : partsper Million : 1H
9.4515
7.2600
7.0380
5.7540
2.2670
2.2624
2.1285
2.1193
1.7131
1.6982
1.6318
1.6169
1.1603
0.0686
−0.0035
3.21
1
2.14
2.17
2.4
2.29
9.66

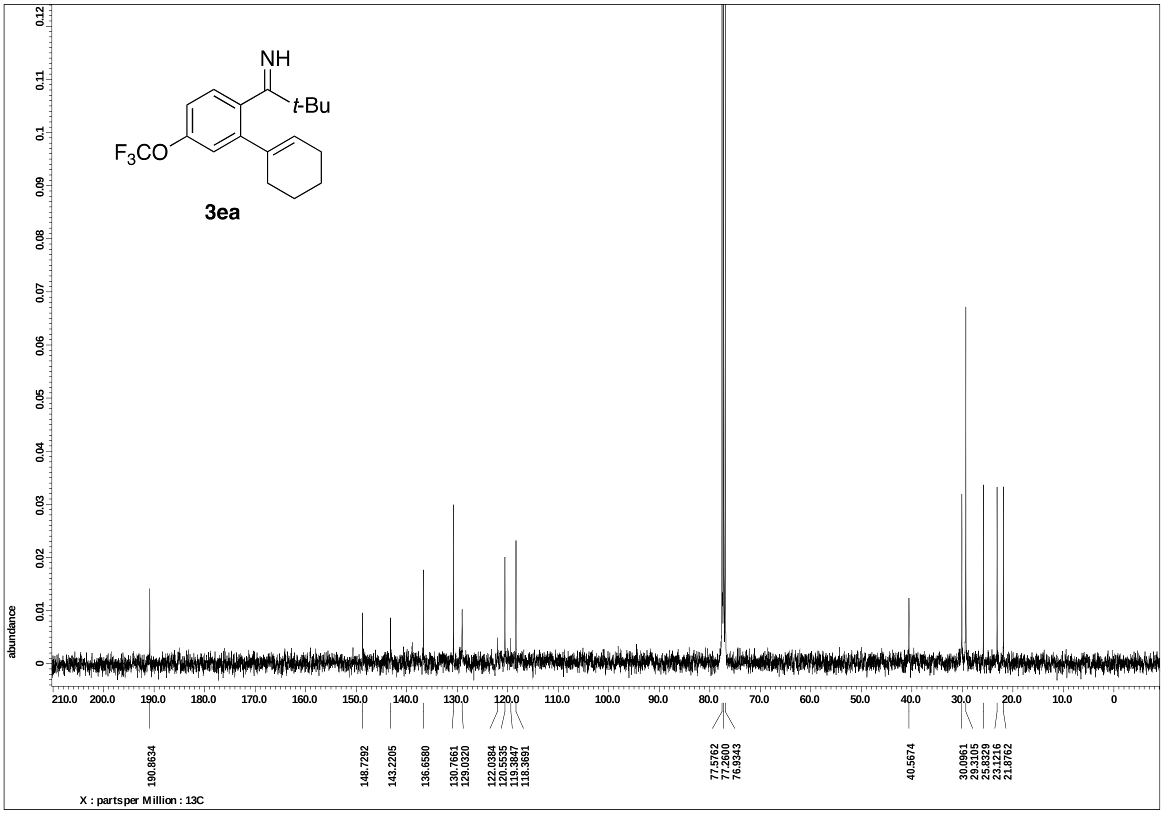
NH
t-Bu
F3CO
3ea
abundance
X : partsper Million : 13C
190.8634
148.7292
143.2205
136.6580
130.7661
129.0320
122.0384
120.5535
119.3847
118.3691
77.5762
77.2600
76.9343
40.5674
30.0961
29.3105
25.8329
23.1216
21.8762

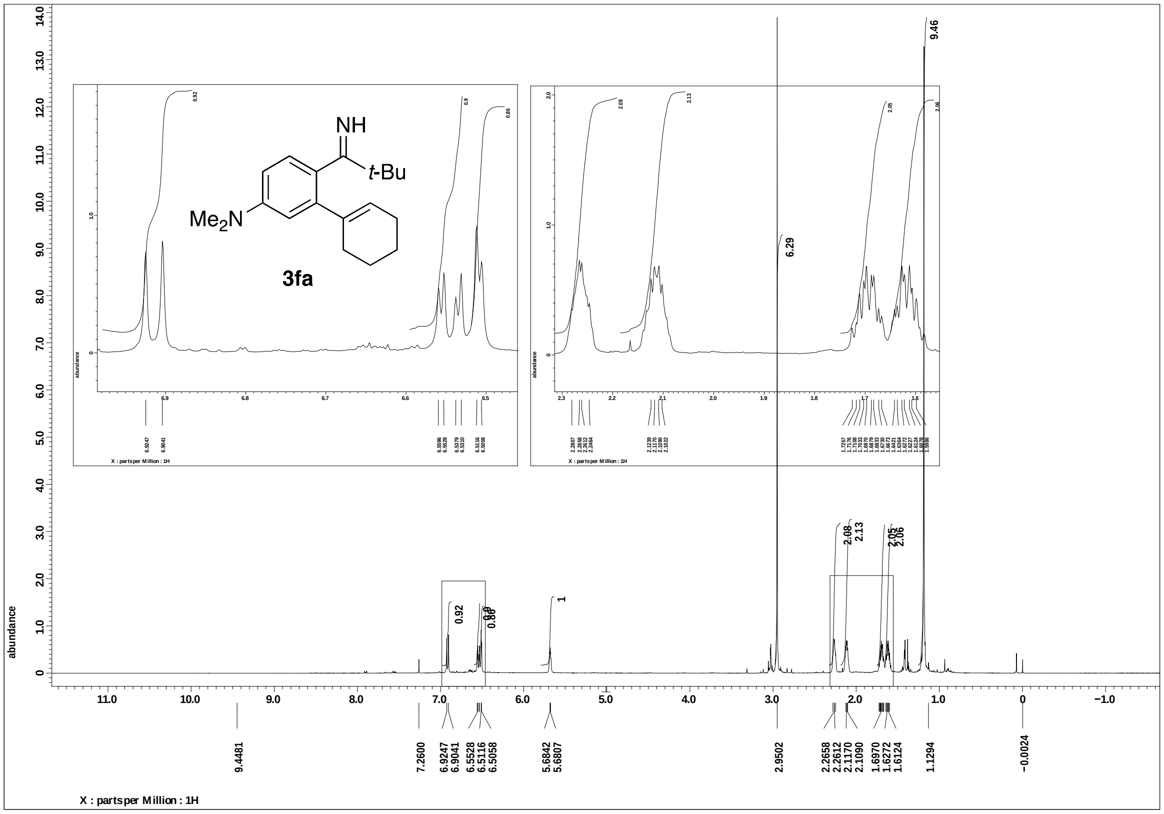
NH
t-Bu
Me2N
3fa
abundance
X : partsper Million : 1H
9.4481
7.2600
6.9247
6.9041
6.5528
6.5116
6.5058
5.6842
5.6807
2.9502
2.2658
2.2612
2.1170
2.1090
1.6970
1.6272
1.6124
1.1294
−0.0024
0.92
1
6.29
2.08
2.13
2.05
2.06
9.46

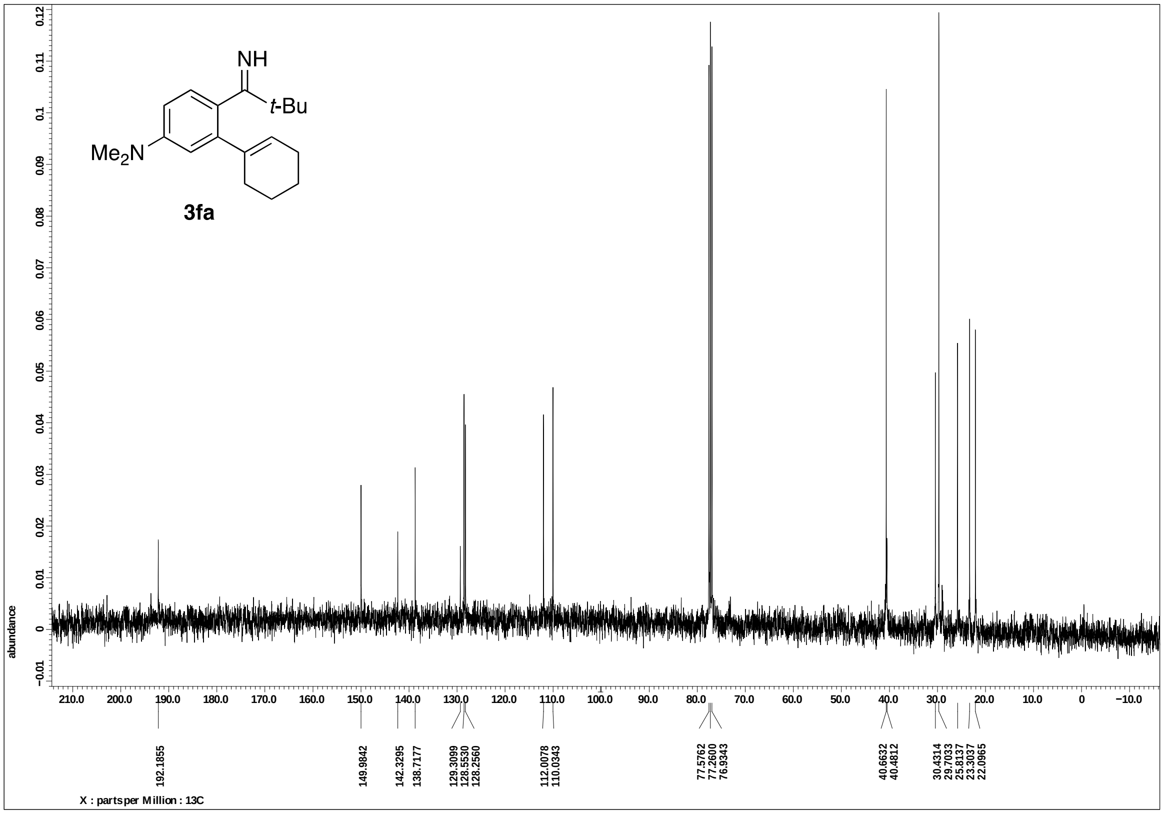
NH
t-Bu
Me2N
3fa
abundance
X : partsper Million : 13C
192.1855
149.9842
142.3295
138.7177
129.3099
128.5530
128.2560
112.0078
110.0343
77.5762
77.2600
76.9343
40.6632
40.4812
30.4314
29.7033
25.8137
23.3037
22.0965

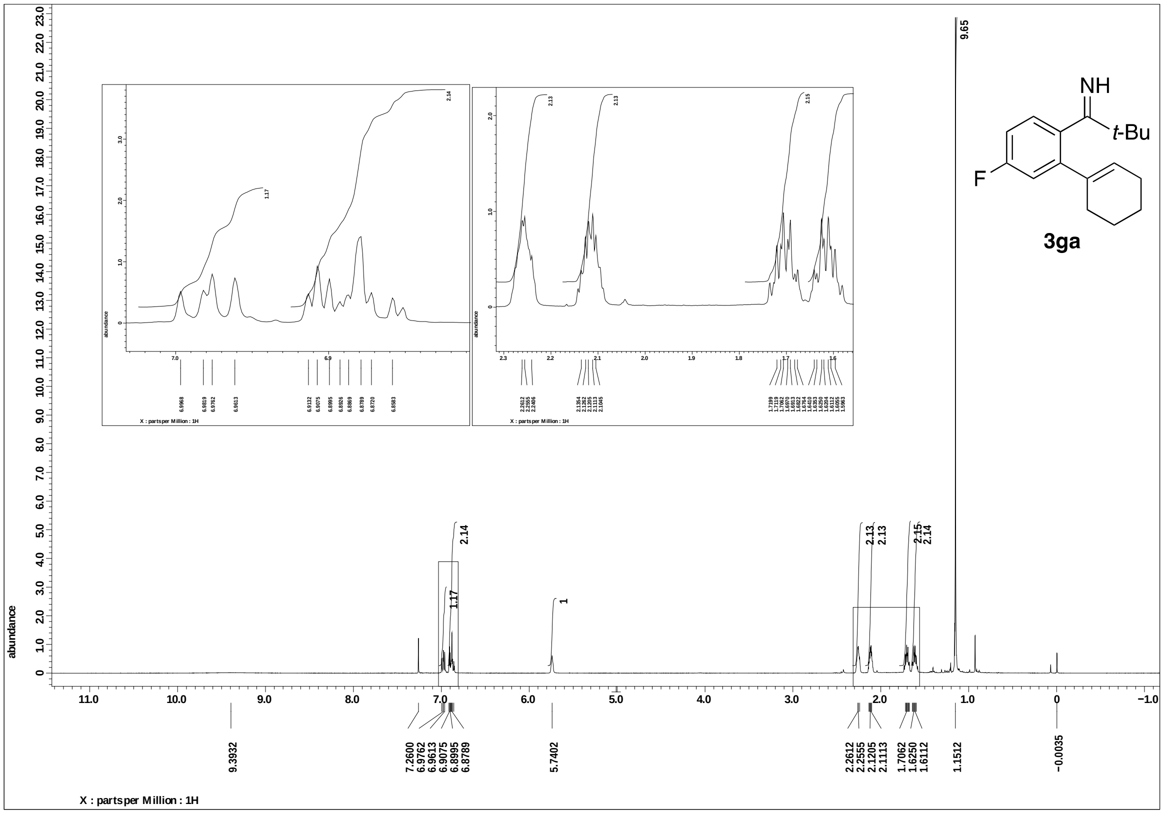
9.65
NH
t-Bu
F
3ga
abundance
X : parts per Million : 1H
9.3932
7.2600
6.9762
6.9613
6.9075
6.8995
6.8789
5.7402
2.2612
2.2555
2.1205
2.1113
1.7062
1.6250
1.6112
1.1512
-0.0035
2.14
1.17
1
2.13
2.13
2.15
2.14

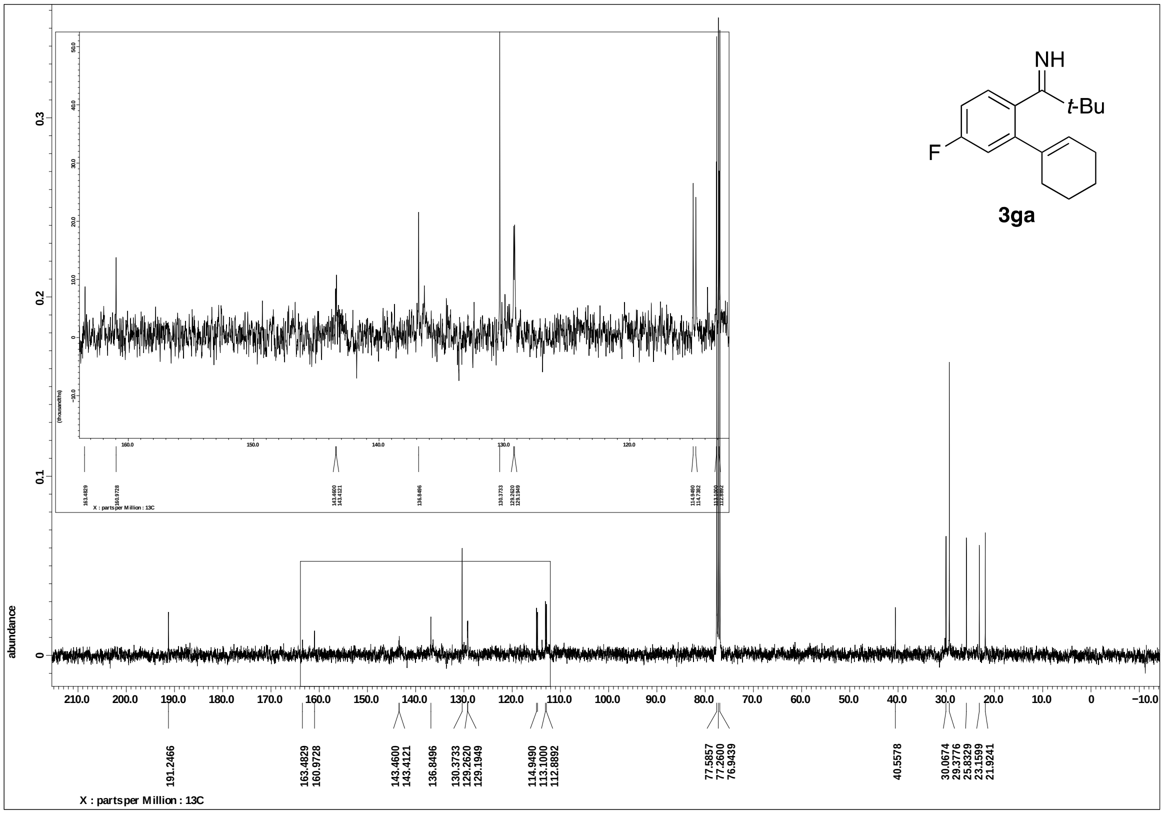
NH
t-Bu
F
3ga
abundance
X : parts per Million : 13C
191.2466
163.4829
160.9728
143.4600
143.4121
136.8496
130.3733
129.2620
129.1949
114.9490
113.1000
112.8892
77.5857
77.2600
76.9439
40.5578
30.0674
29.3776
25.8329
23.1599
21.9241

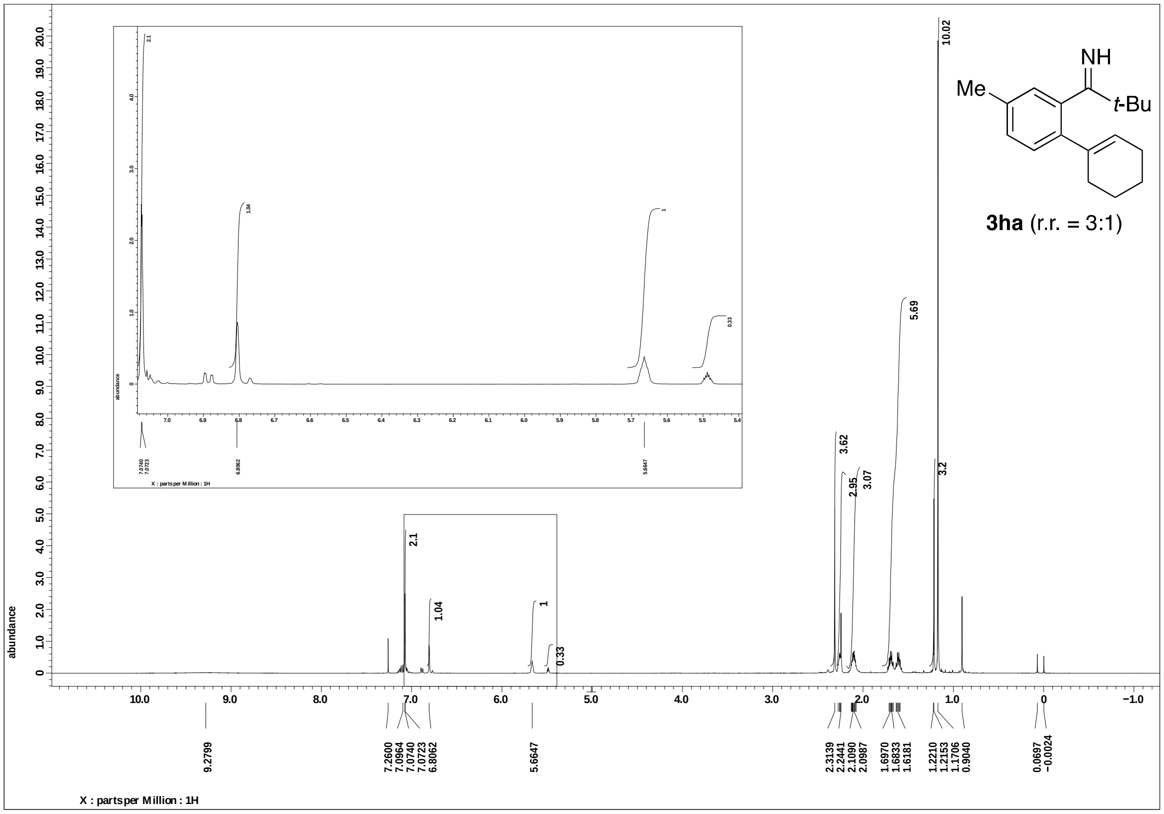

Me
NH
t-Bu
3ha (r.r. = 3:1)
abundance
X : parts per Million : 1H
9.2799
7.2600
7.0964
7.0740
7.0723
6.8062
5.6647
2.3139
2.2441
2.1090
2.0987
1.6970
1.6833
1.6181
1.2210
1.2153
1.1706
0.9040
0.0697
−0.0024
2.1
1.04
1
0.33
3.62
2.95
3.07
5.69
3.2
10.02

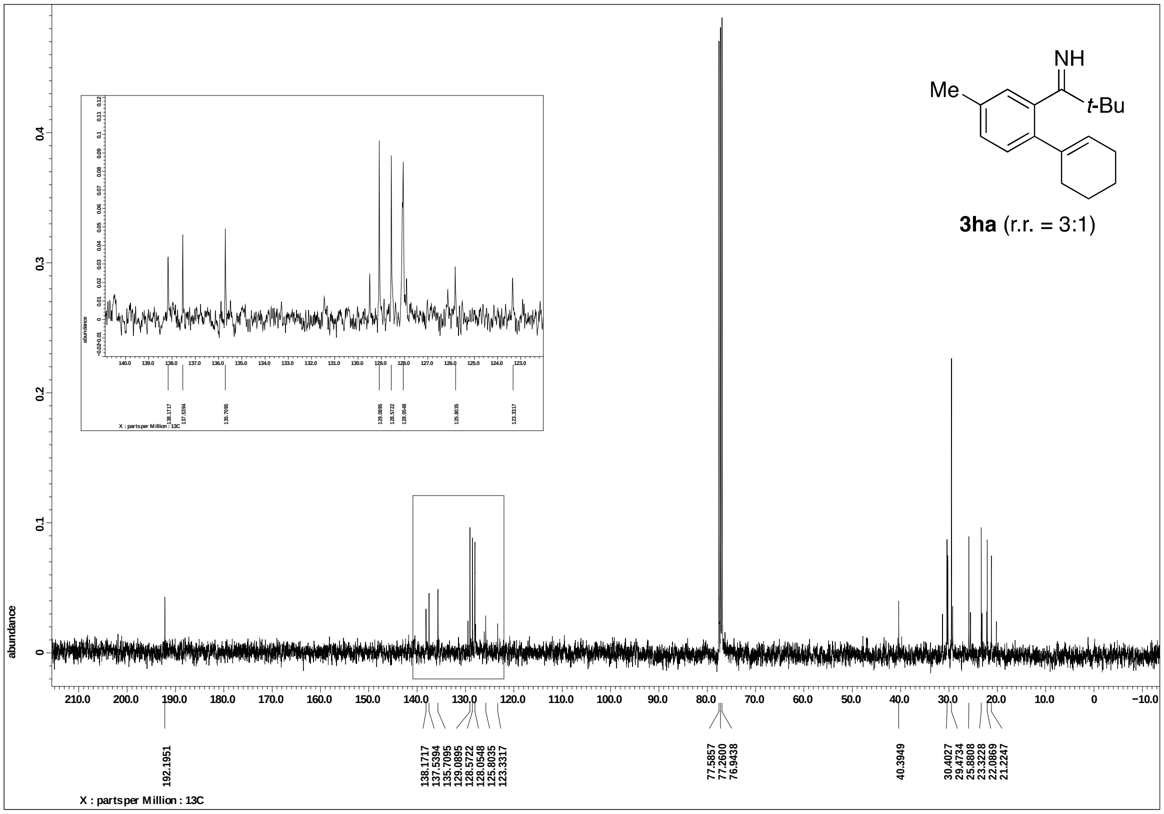

Me
NH
t-Bu
3ha (r.r. = 3:1)
abundance
X : parts per Million : 13C
192.1951
138.1717
137.5394
135.7095
129.0895
128.5722
128.0548
125.8035
123.3317
77.5857
77.2600
76.9438
40.3949
30.4027
29.4734
25.8808
23.3228
22.0869
21.2247

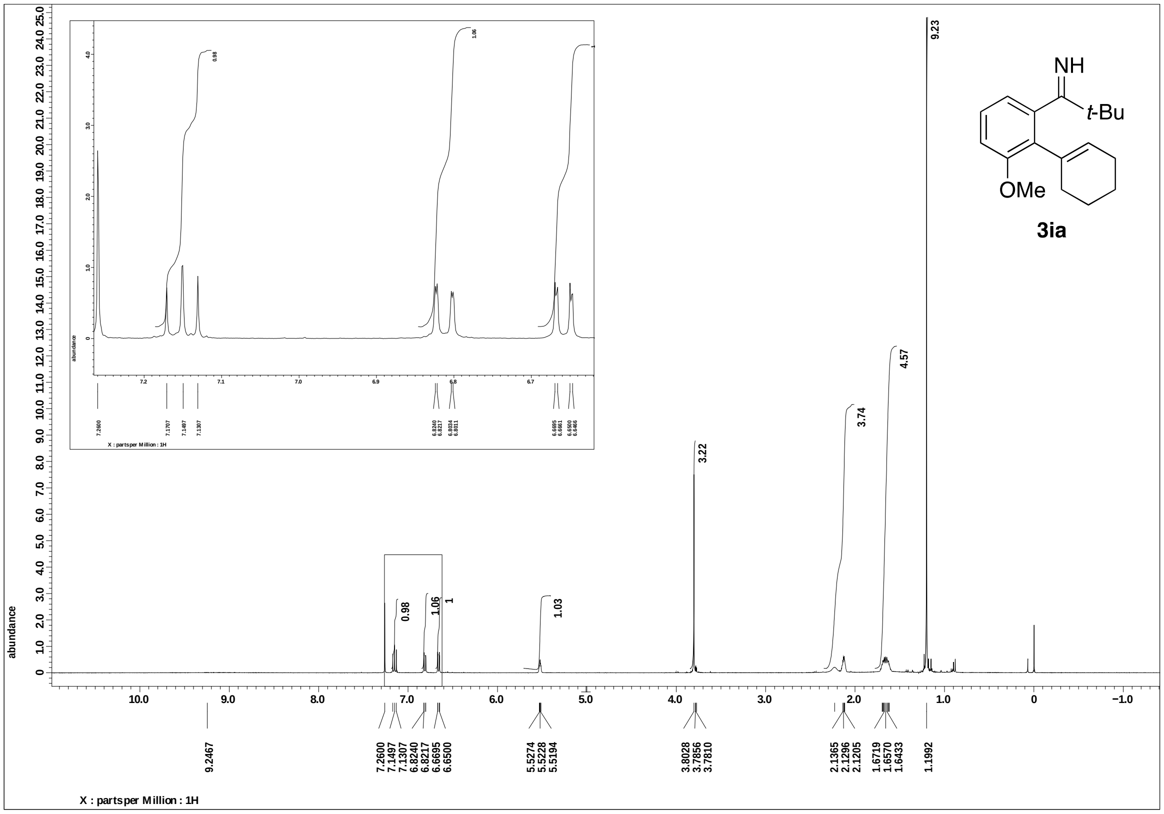
NH
t-Bu
OMe
3ia
abundance
X : partsper Million : 1H
9.23
3.22
3.74
4.57
0.98
1.06
1
1.03
9.2467
7.2600
7.1497
7.1307
6.8240
6.8217
6.6695
6.6500
5.5274
5.5228
5.5194
3.8028
3.7856
3.7810
2.1365
2.1296
2.1205
1.6719
1.6570
1.6433
1.1992

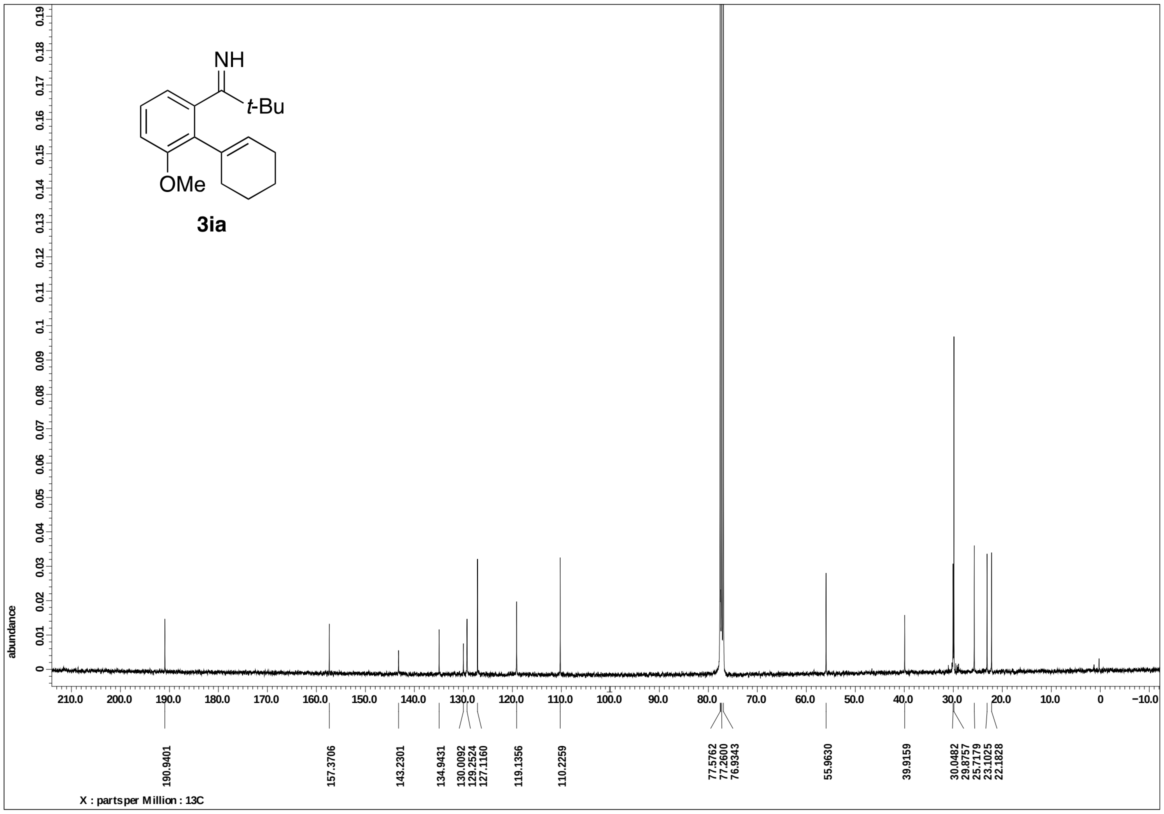
NH
t-Bu
OMe
3ia
abundance
X : partsper Million : 13C
190.9401
157.3706
143.2301
134.9431
130.0092
129.2524
127.1160
119.1356
110.2259
77.5762
77.2600
76.9343
55.9630
39.9159
30.0482
29.8757
25.7179
23.1025
22.1828

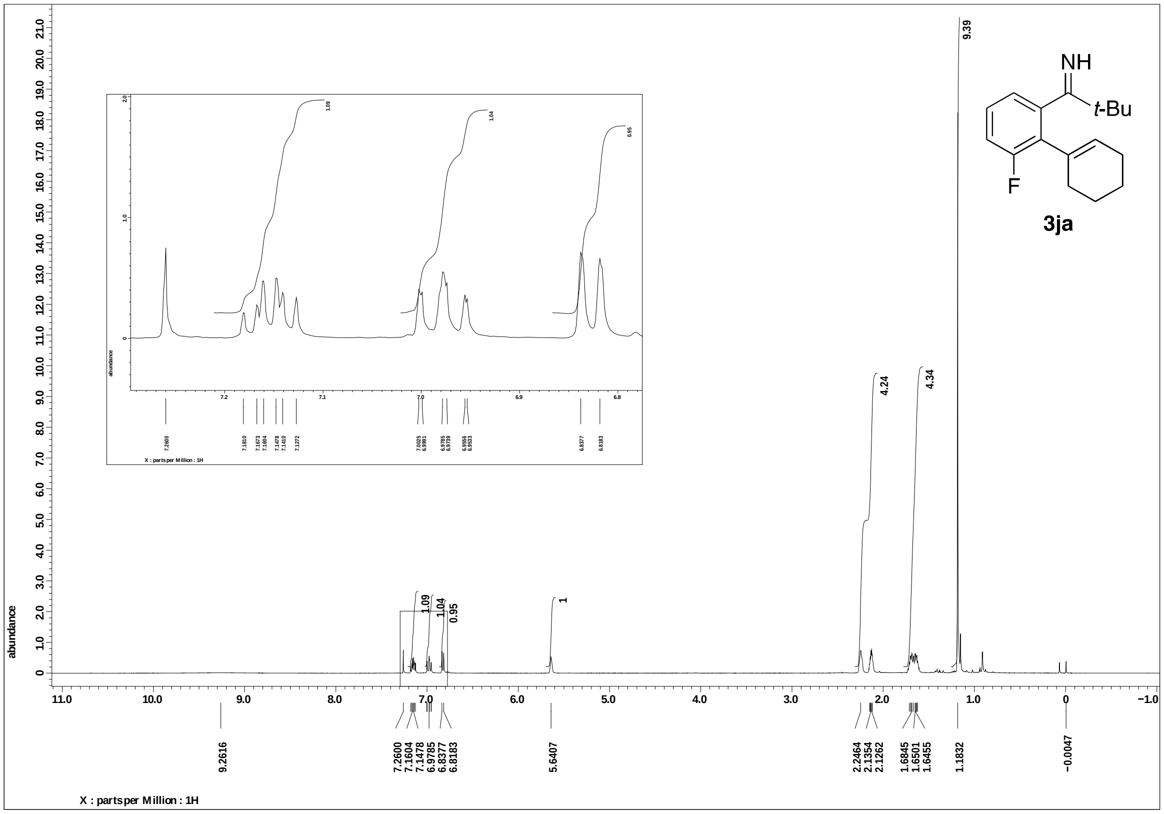
NH
t-Bu
F
3ja
abundance
X : partsper Million : 1H
9.2616
7.2600
7.1604
7.1478
6.9785
6.8377
6.8183
5.6407
2.2464
2.1354
2.1262
1.6845
1.6501
1.6455
1.1832
−0.0047
9.39
4.24
4.34
1.09
1.04
0.95

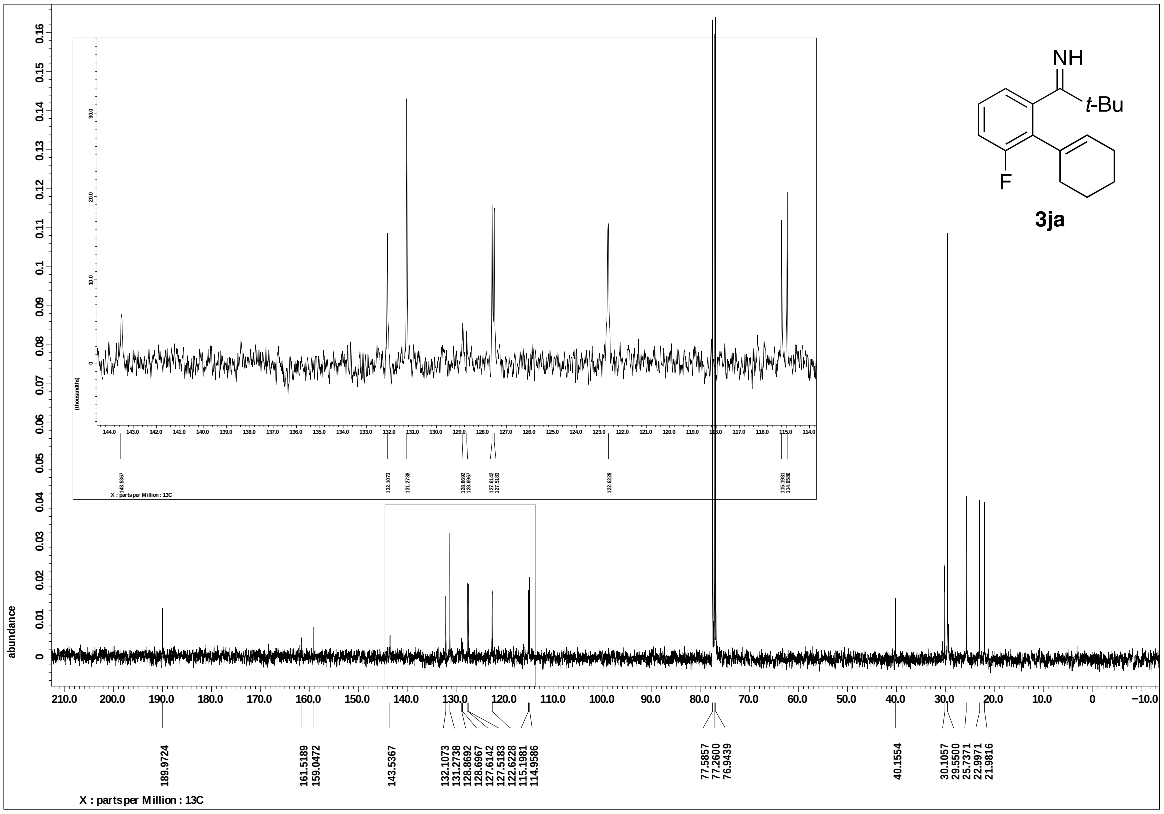
NH
t-Bu
F
3ja
abundance
X : partsper Million : 13C
189.9724
161.5189
159.0472
143.5367
132.1073
131.2738
128.8692
128.6967
127.6142
127.5183
122.6228
115.1981
114.9586
77.5857
77.2600
76.9439
40.1554
30.1057
29.5500
25.7371
22.9971
21.9816

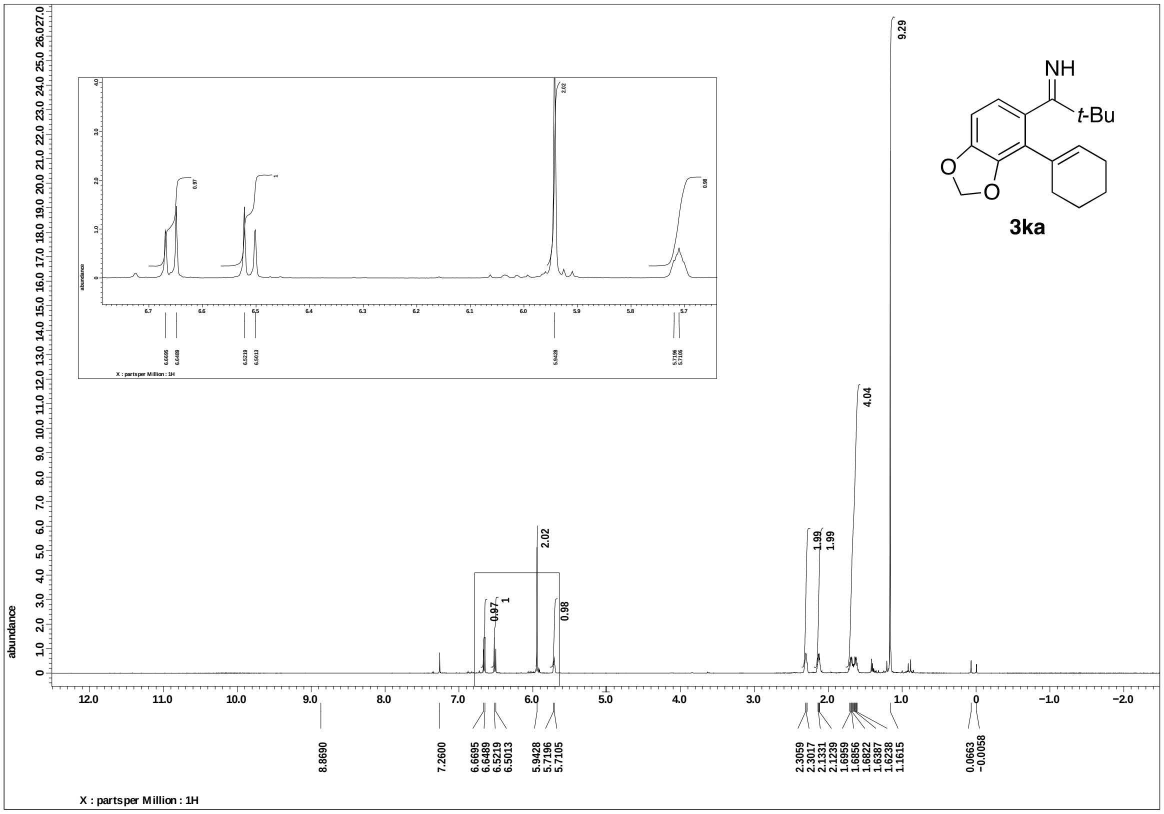
NH
t-Bu
O
O
3ka
X : partsper Million : 1H
abundance

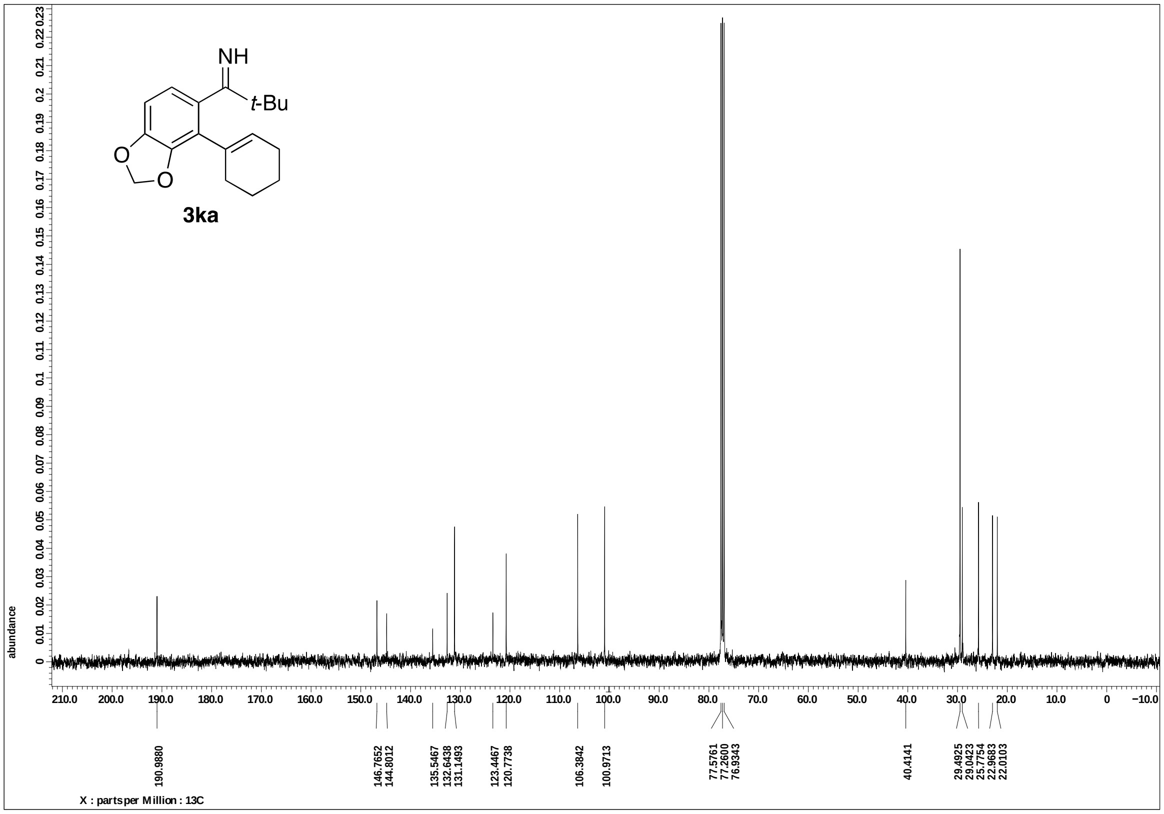
NH
t-Bu
O
O
3ka
X : partsper Million : 13C
abundance

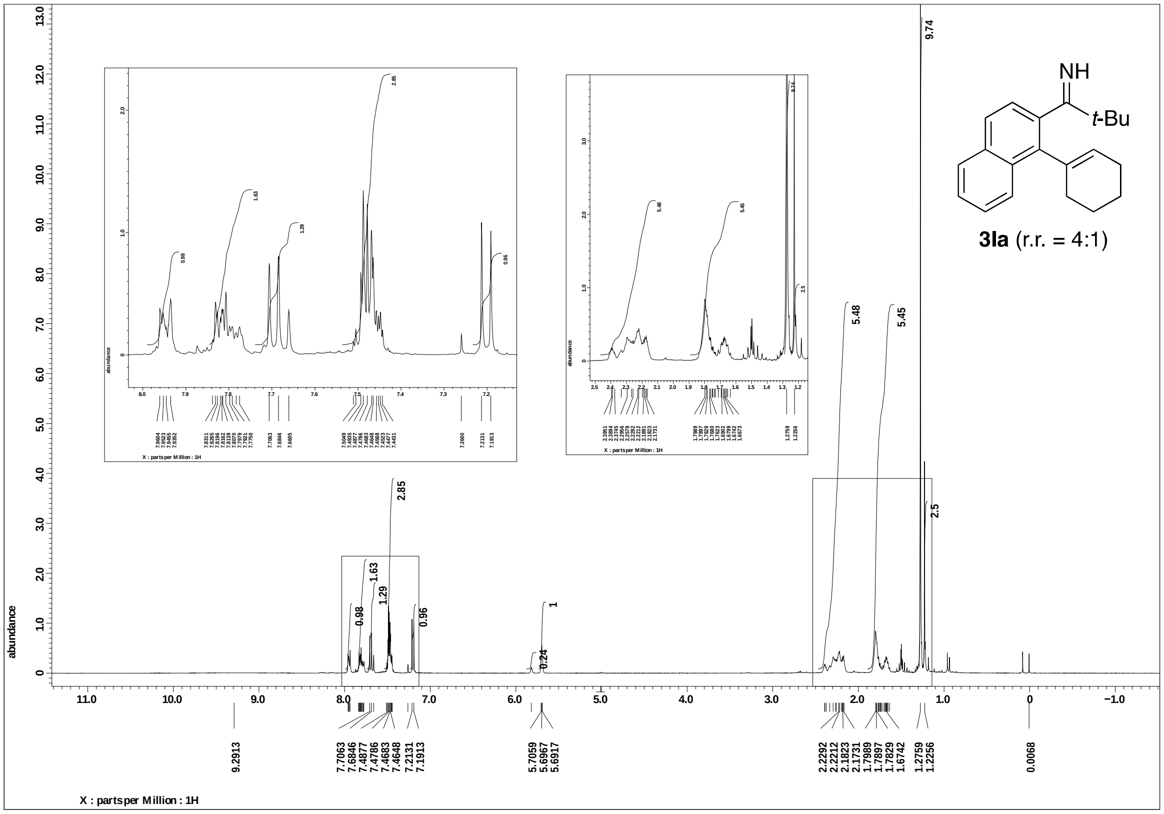
NH
t-Bu
3la (r.r. = 4:1)
abundance
X : parts per Million : 1H
9.2913
7.7063
7.6846
7.4877
7.4786
7.4683
7.4648
7.2131
7.1913
5.7059
5.6967
5.6917
2.2292
2.2212
2.1823
2.1731
1.7989
1.7897
1.7829
1.6742
1.2759
1.2256
0.0068
9.74
5.48
5.45
2.85
2.5
1.63
1.29
0.98
0.96
1
0.24

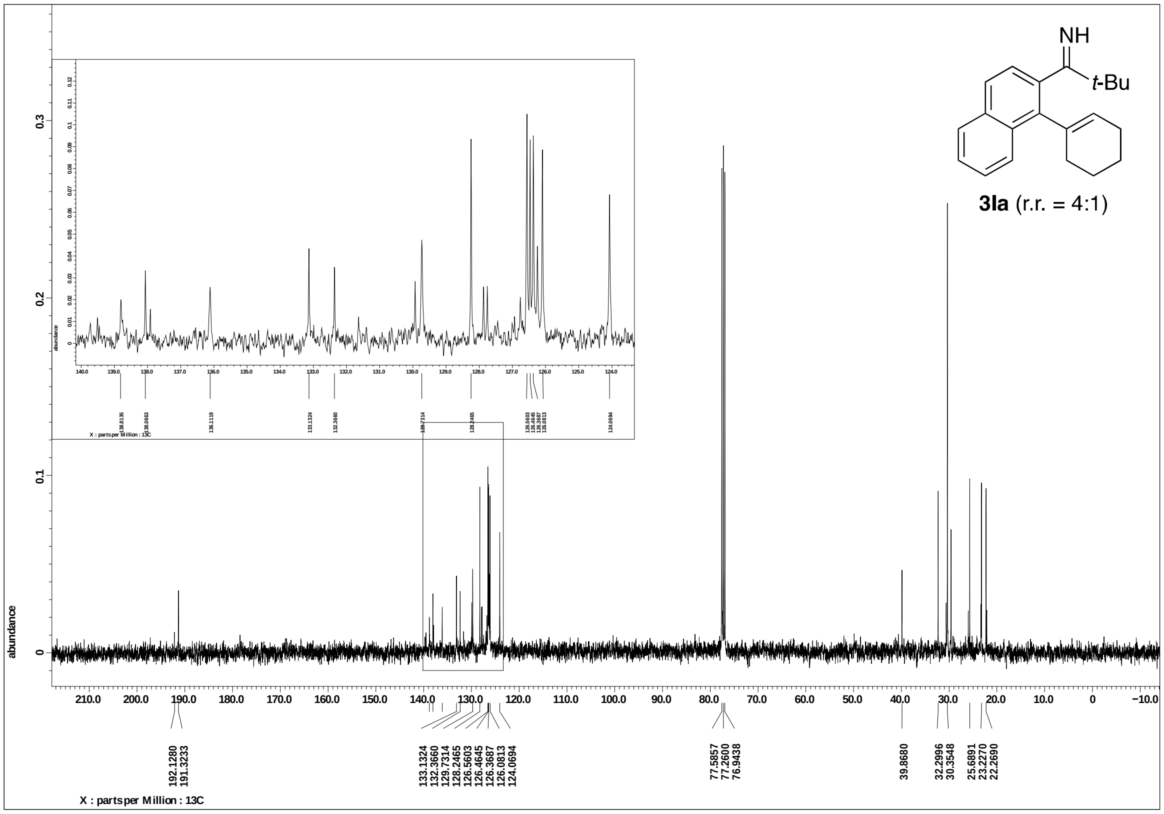
NH
t-Bu
3la (r.r. = 4:1)
abundance
X : parts per Million : 13C
192.1280
191.3233
133.1324
132.3660
129.7314
128.2465
126.5603
126.4645
126.3687
126.0813
124.0694
77.5857
77.2600
76.9438
39.8680
32.2996
30.3548
25.6891
23.2270
22.2690

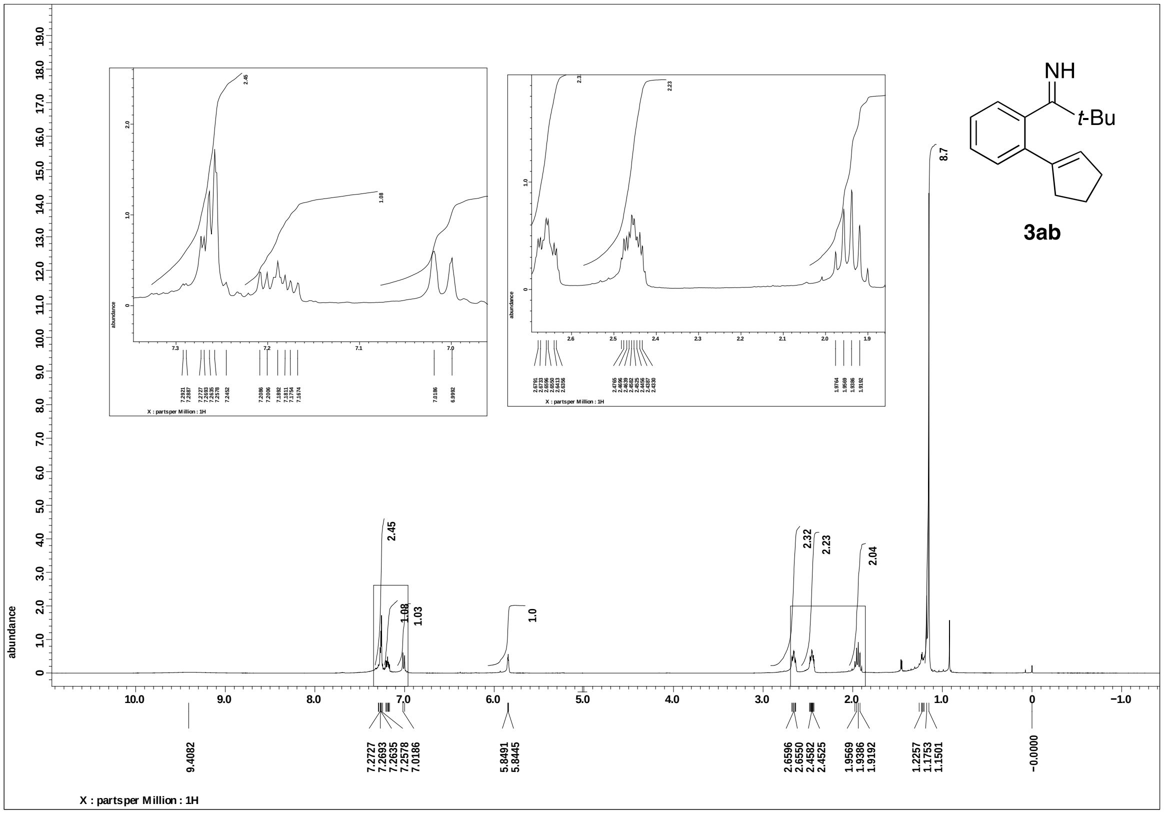
NH
t-Bu
3ab
abundance
X : partsper Million : 1H
9.4082
7.2727
7.2693
7.2635
7.2578
7.0186
5.8491
5.8445
2.6596
2.6550
2.4582
2.4525
1.9569
1.9386
1.9192
1.2257
1.1753
1.1501
−0.0000
2.45
1.08
1.03
1.0
2.32
2.23
2.04
8.7

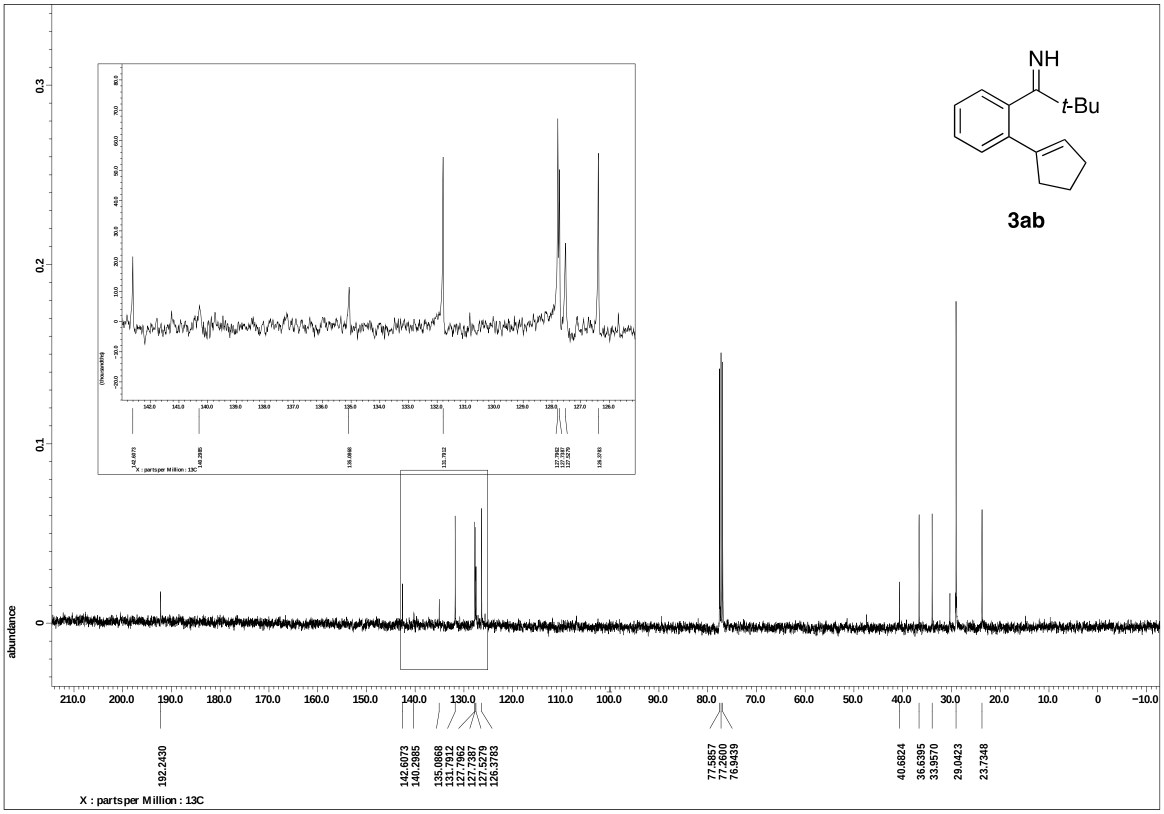
NH
t-Bu
3ab
abundance
X : partsper Million : 13C
192.2430
142.6073
140.2985
135.0868
131.7912
127.7962
127.7387
127.5279
126.3783
77.5857
77.2600
76.9439
40.6824
36.6395
33.9570
29.0423
23.7348

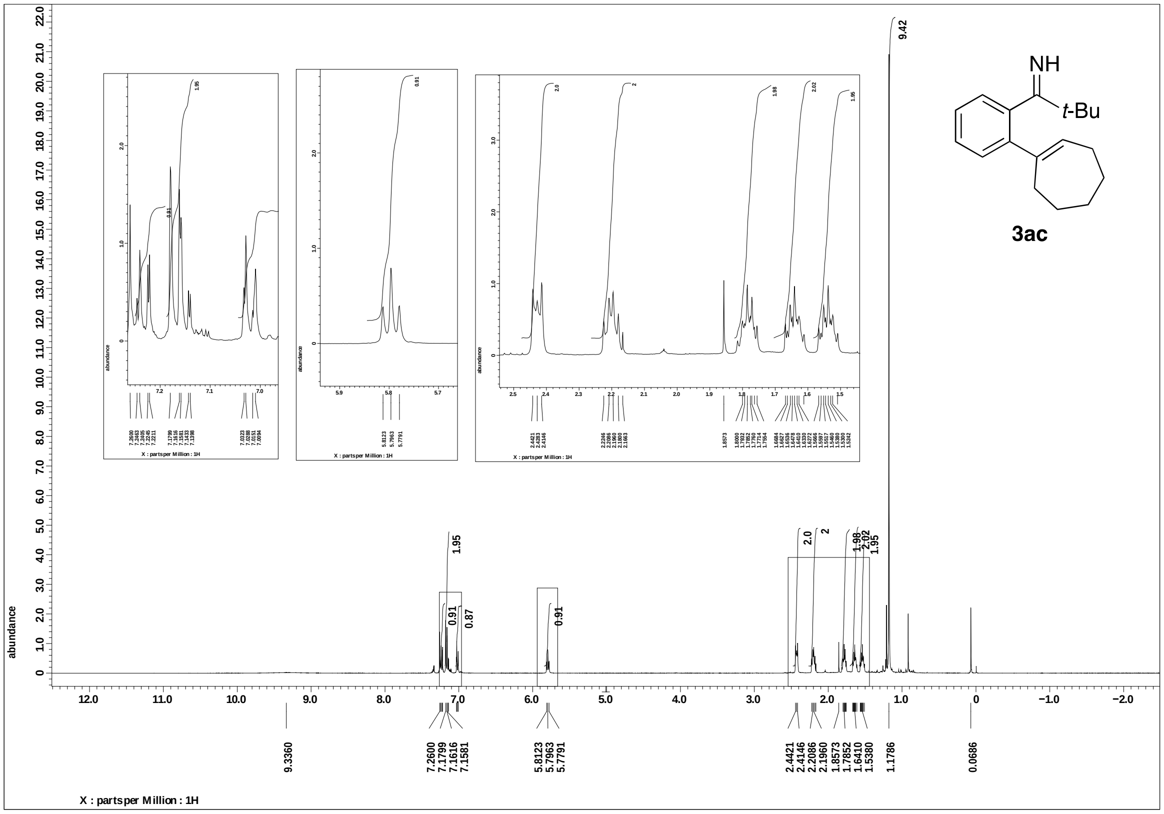

abundance
X : parts per Million : 1H
9.3360
7.2600
7.1799
7.1616
7.1581
5.8123
5.7963
5.7791
2.4421
2.4146
2.2086
2.1960
1.8573
1.7852
1.6410
1.5380
1.1786
0.0686
9.42
1.95
0.91
0.87
0.91
2.0
2
1.98
2.02
1.95
NH
t-Bu
3ac

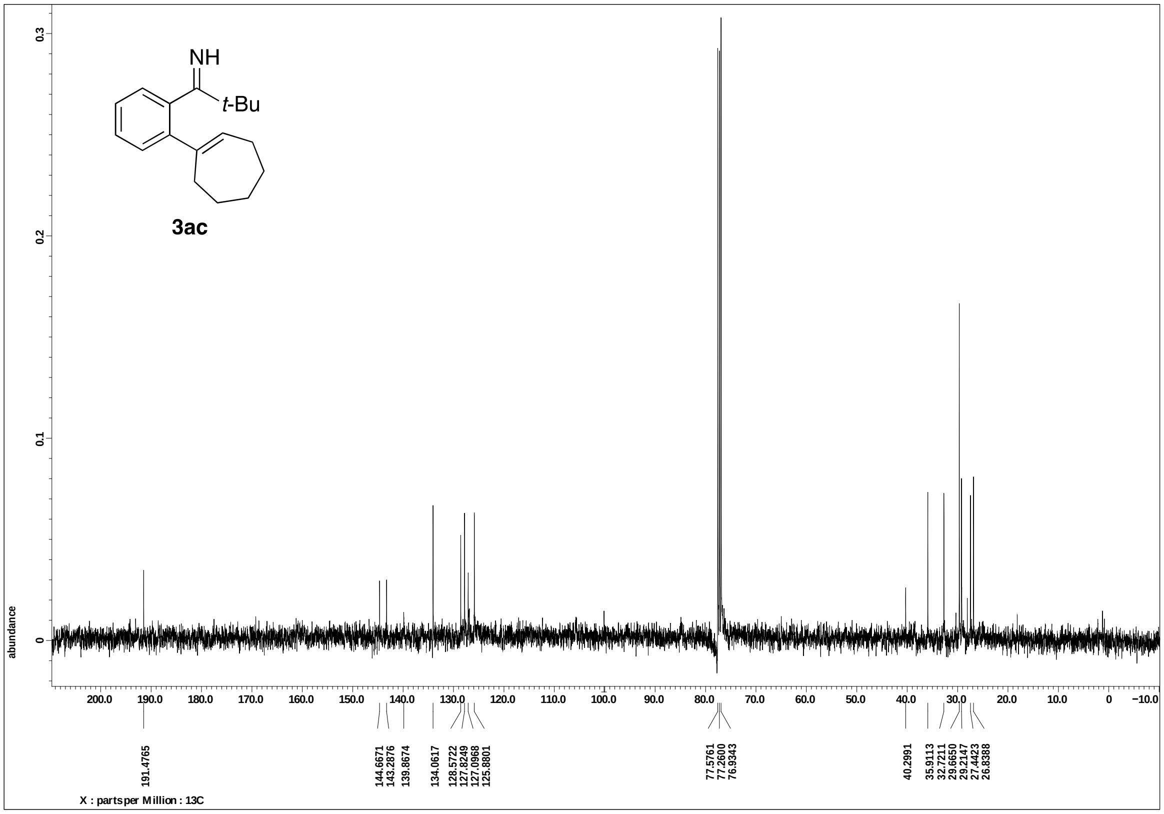

abundance
X : parts per Million : 13C
191.4765
144.6671
143.2876
139.8674
134.0617
128.5722
127.8249
127.0968
125.8801
77.5761
77.2600
76.9343
40.2991
35.9113
32.7211
29.6650
29.2147
27.4423
26.8388
NH
t-Bu
3ac

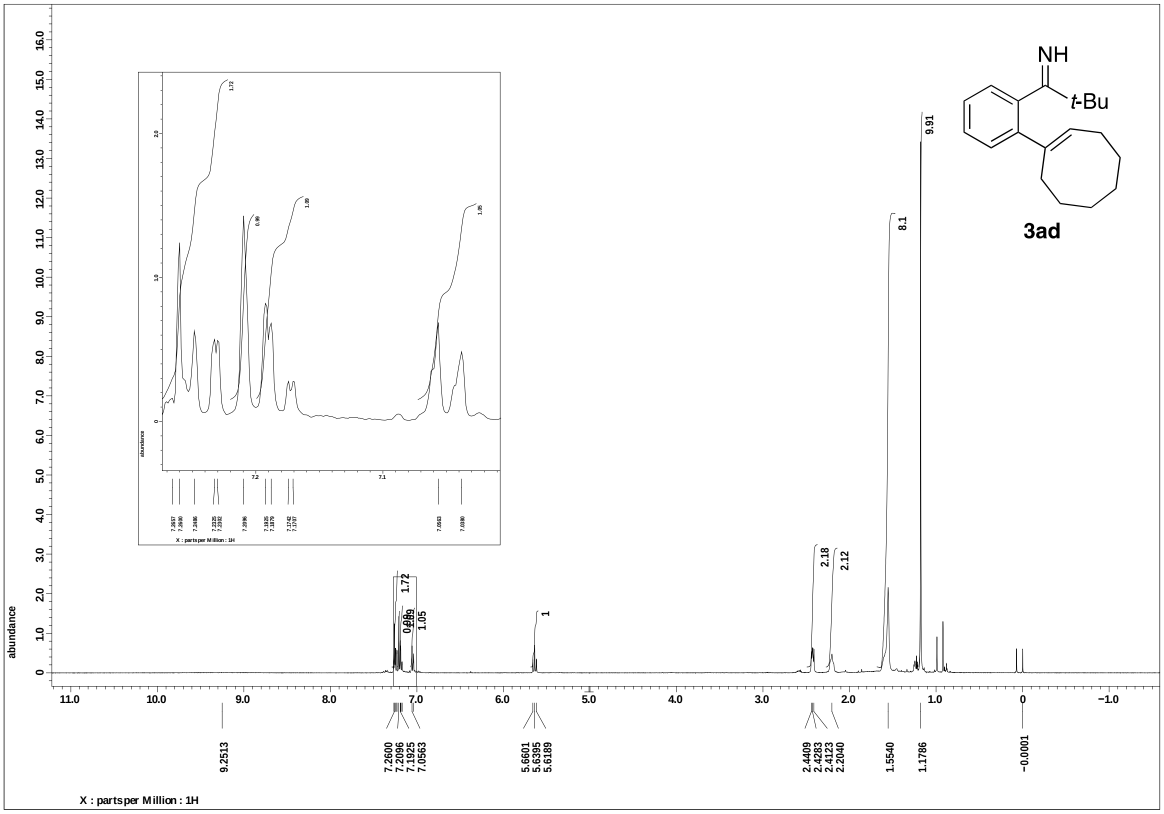

NH
t-Bu
3ad
abundance
X : parts per Million : 1H
9.2513
7.2600
7.2096
7.1925
7.0563
5.6601
5.6395
5.6189
2.4409
2.4283
2.4123
2.2040
1.5540
1.1786
−0.0001
2.18
2.12
8.1
9.91
1.72
1.05
1

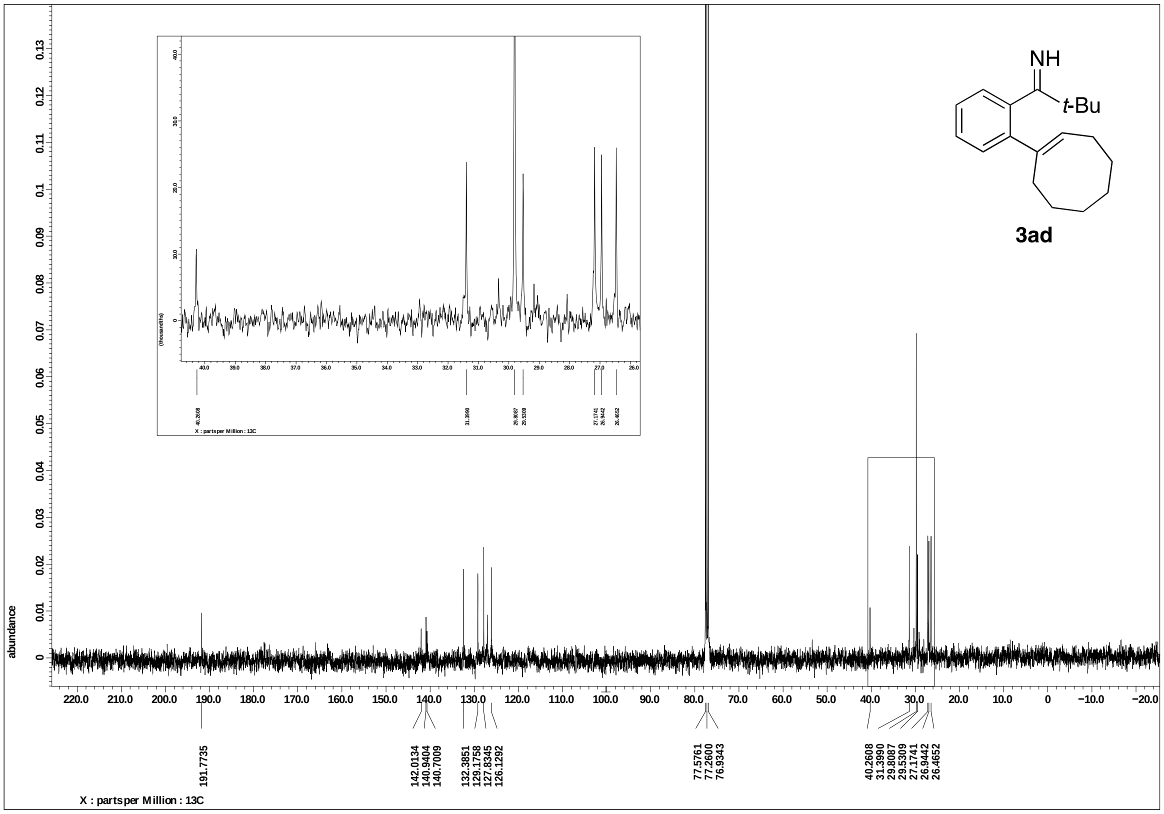

NH
t-Bu
3ad
abundance
X : parts per Million : 13C
191.7735
142.0134
140.9404
140.7009
132.3851
129.1758
127.8345
126.1292
77.5761
77.2600
76.9343
40.2608
31.3990
29.8087
29.5309
27.1741
26.9442
26.4652

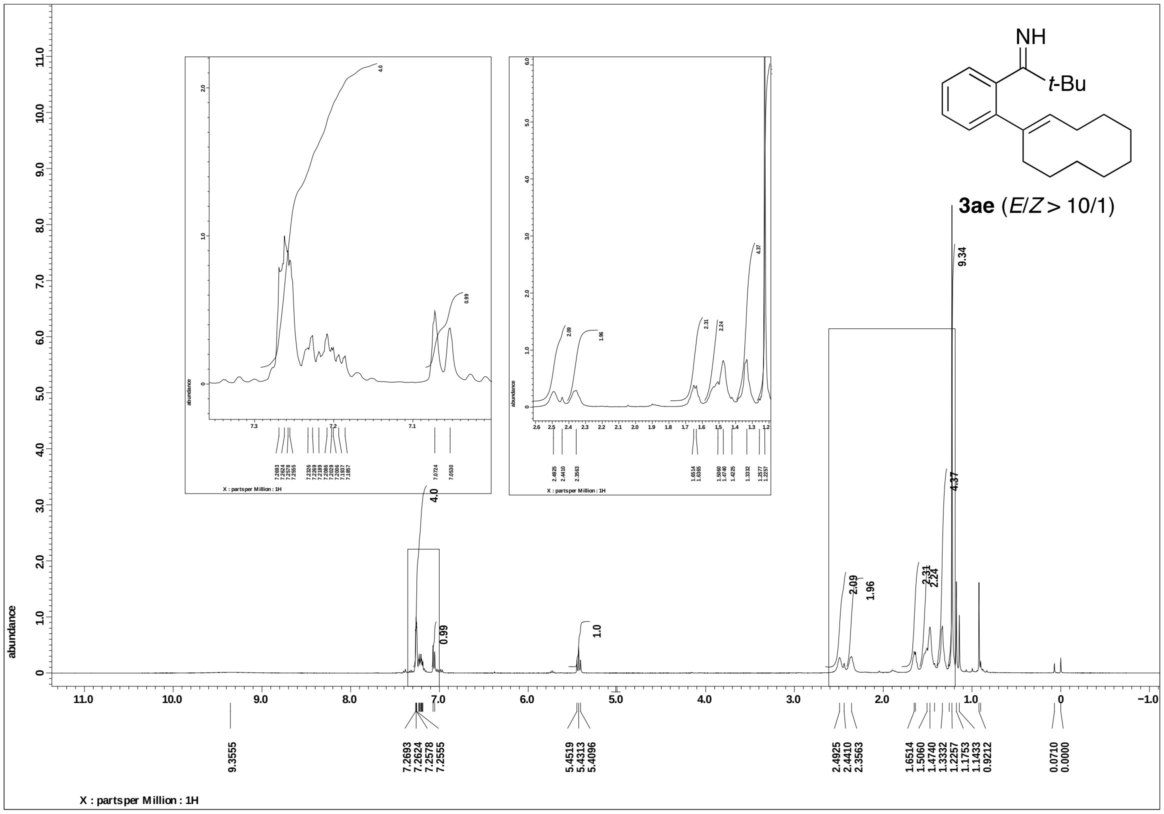

NH
t-Bu
3ae (E/Z > 10/1)
abundance
X : partsper Million : 1H
9.3555
7.2693
7.2624
7.2578
7.2555
5.4519
5.4313
5.4096
2.4925
2.4410
2.3563
1.6514
1.5060
1.4740
1.3332
1.2257
1.1753
1.1423
0.9212
0.0710
0.0000

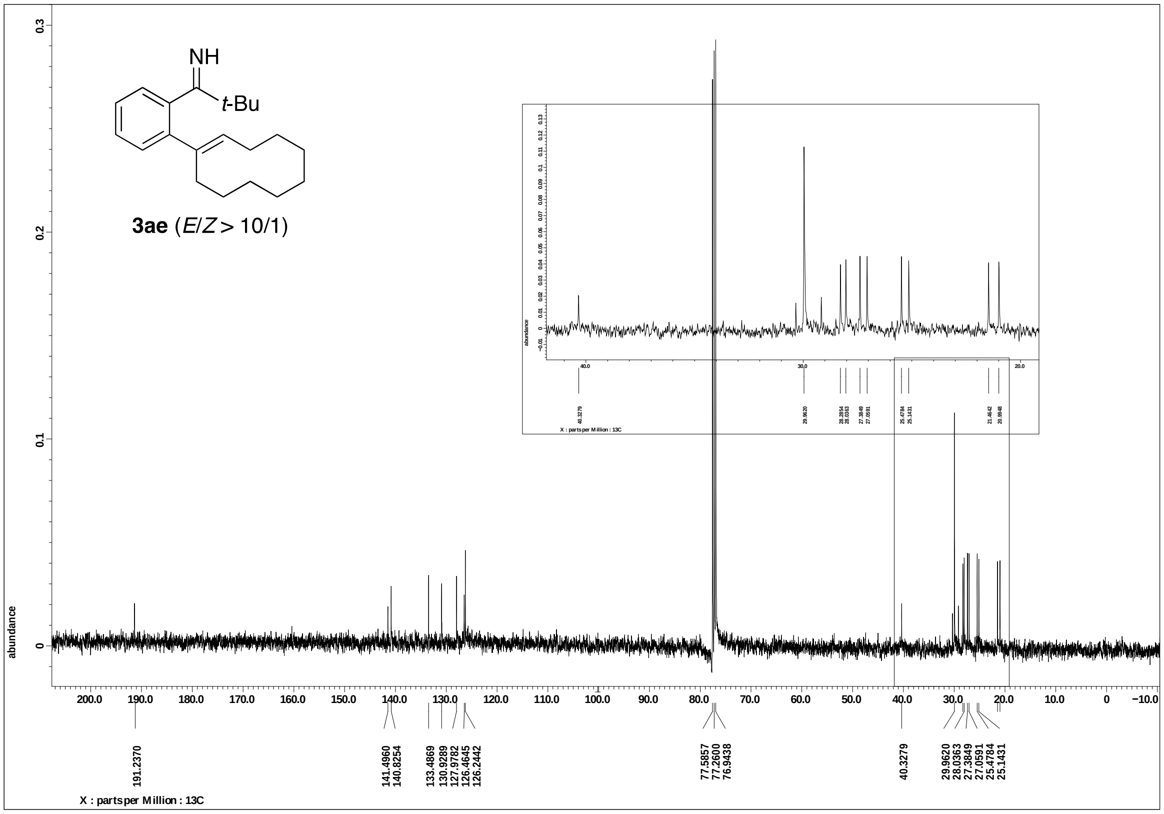

NH
t-Bu
3ae (E/Z > 10/1)
abundance
X : partsper Million : 13C
191.2370
141.4960
140.8254
133.4869
130.9289
127.9782
126.4645
126.2442
77.5857
77.2600
76.9438
40.3279
29.9620
28.0363
27.3849
27.0591
25.4784
25.1431

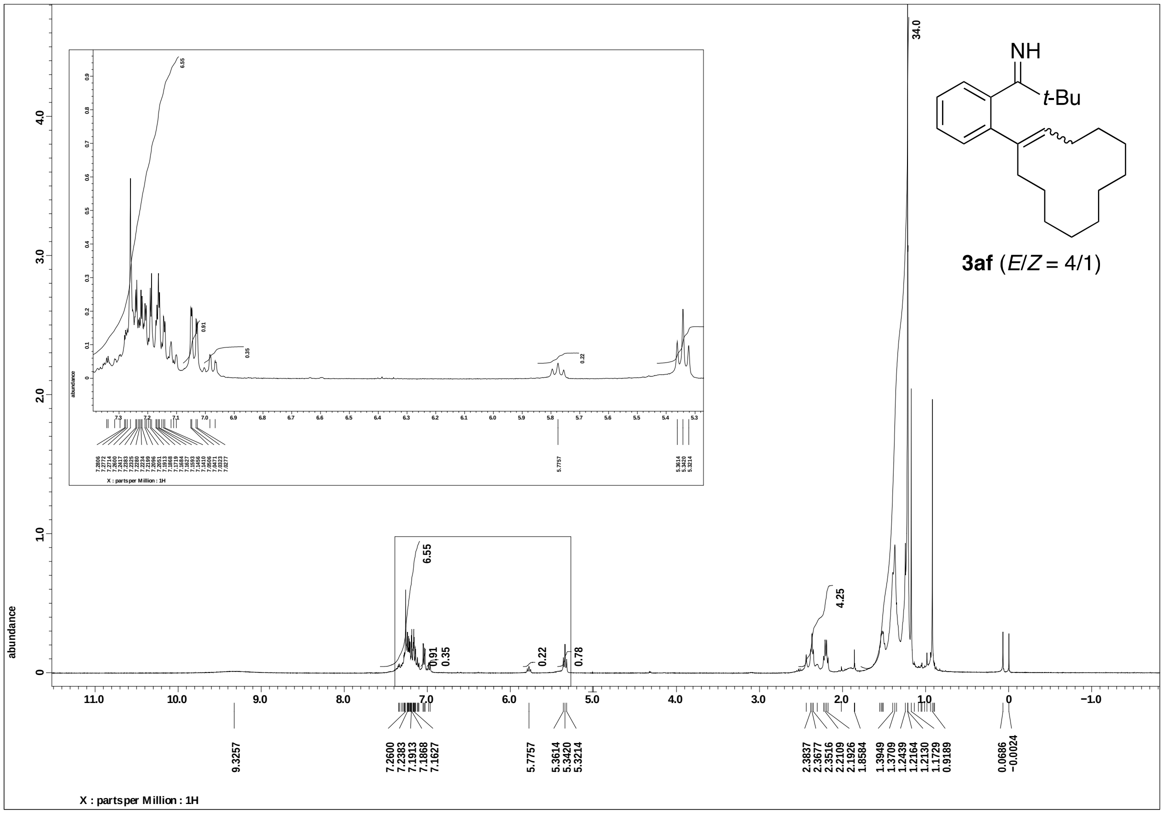

NH
t-Bu
3af (E/Z = 4/1)
abundance
X : partsper Million : 1H
34.0
6.55
0.91
0.35
0.22
0.78
4.25
9.3257
7.2600
7.2383
7.1913
7.1868
7.1627
5.7757
5.3614
5.3420
5.3214
2.3837
2.3677
2.3516
2.2109
2.1926
1.8584
1.3949
1.3709
1.2439
1.2164
1.2130
1.1729
0.9189
0.0686
−0.0024

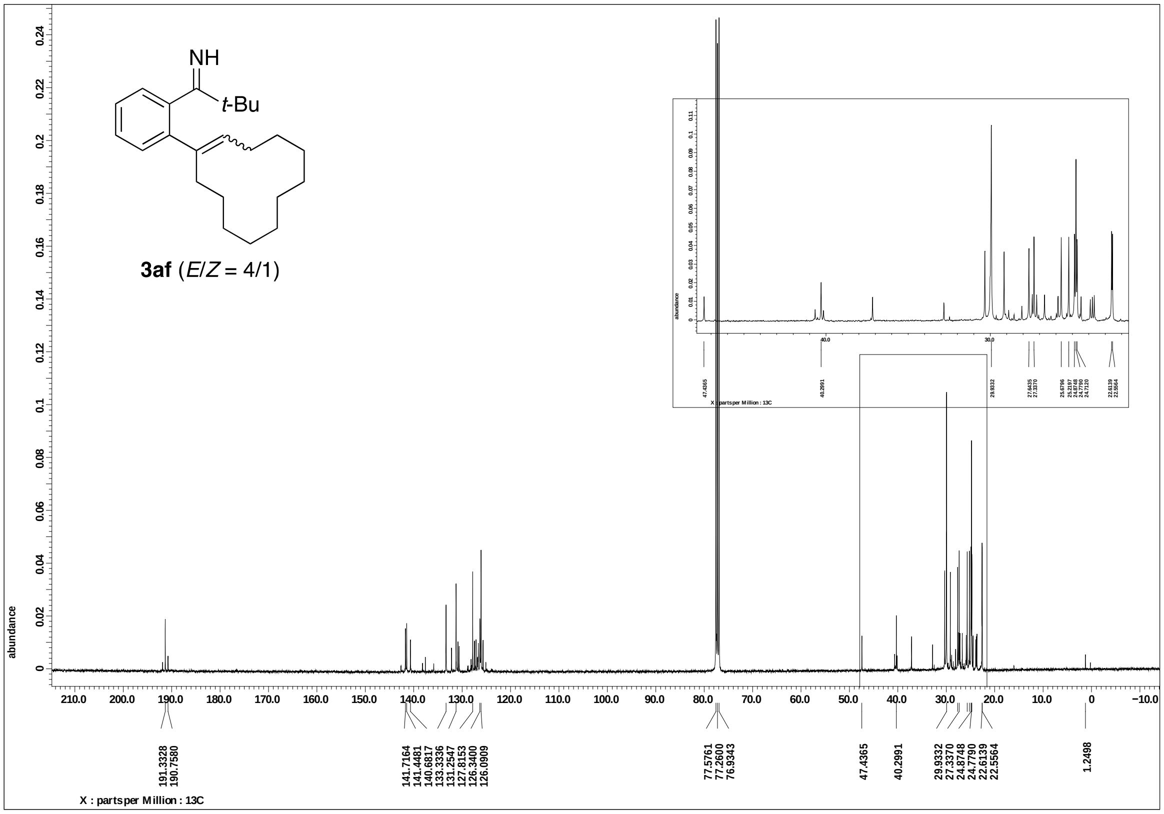

NH
t-Bu
3af (E/Z = 4/1)
abundance
X : partsper Million : 13C
191.3328
190.7580
141.7164
141.4481
140.6817
133.3336
131.2547
127.8153
126.3400
126.0909
77.5761
77.2600
76.9343
47.4365
40.2991
29.9332
27.3370
24.8748
24.7790
22.6139
22.5564
1.2498

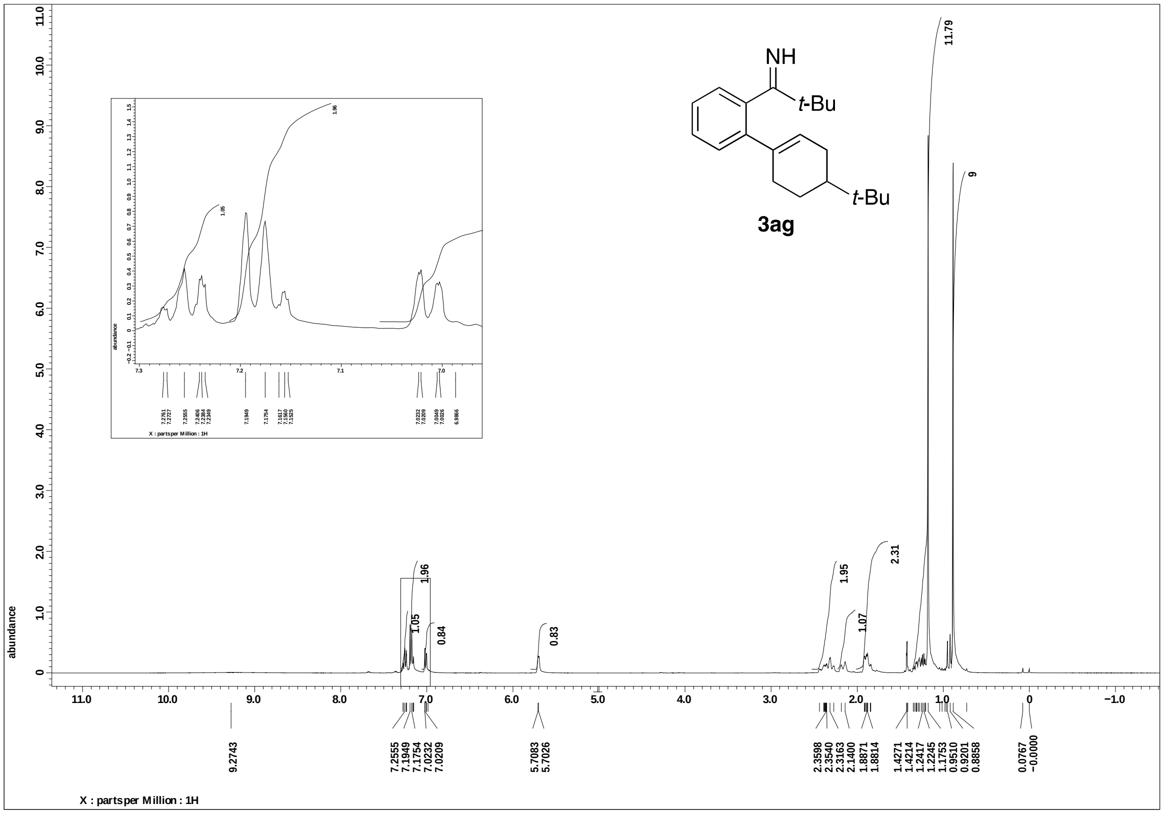
NH
t-Bu
t-Bu
3ag
abundance
X : partsper Million : 1H
9.2743
7.2555
7.1949
7.1754
7.0232
7.0209
5.7083
5.7026
2.3598
2.3540
2.3163
2.1400
1.8871
1.8814
1.4271
1.4214
1.2417
1.2245
1.1753
0.9510
0.9201
0.8858
0.0767
−0.0000
1.05
1.96
0.84
0.83
1.95
1.07
2.31
11.79
9

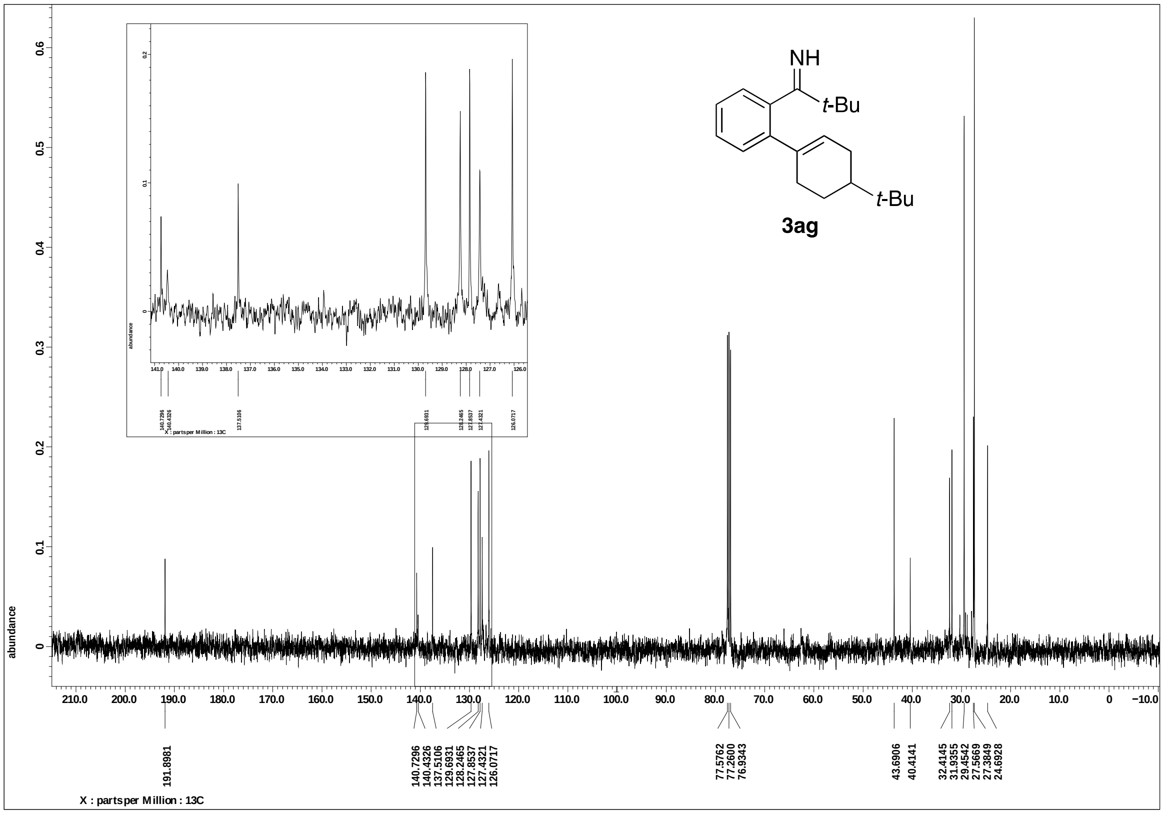
NH
t-Bu
t-Bu
3ag
abundance
X : partsper Million : 13C
191.8981
140.7296
140.4326
137.5106
129.6931
128.2465
127.8537
127.4321
126.0717
77.5762
77.2600
76.9343
43.6906
40.4141
32.4145
31.9355
29.4542
27.5669
27.3849
24.6928

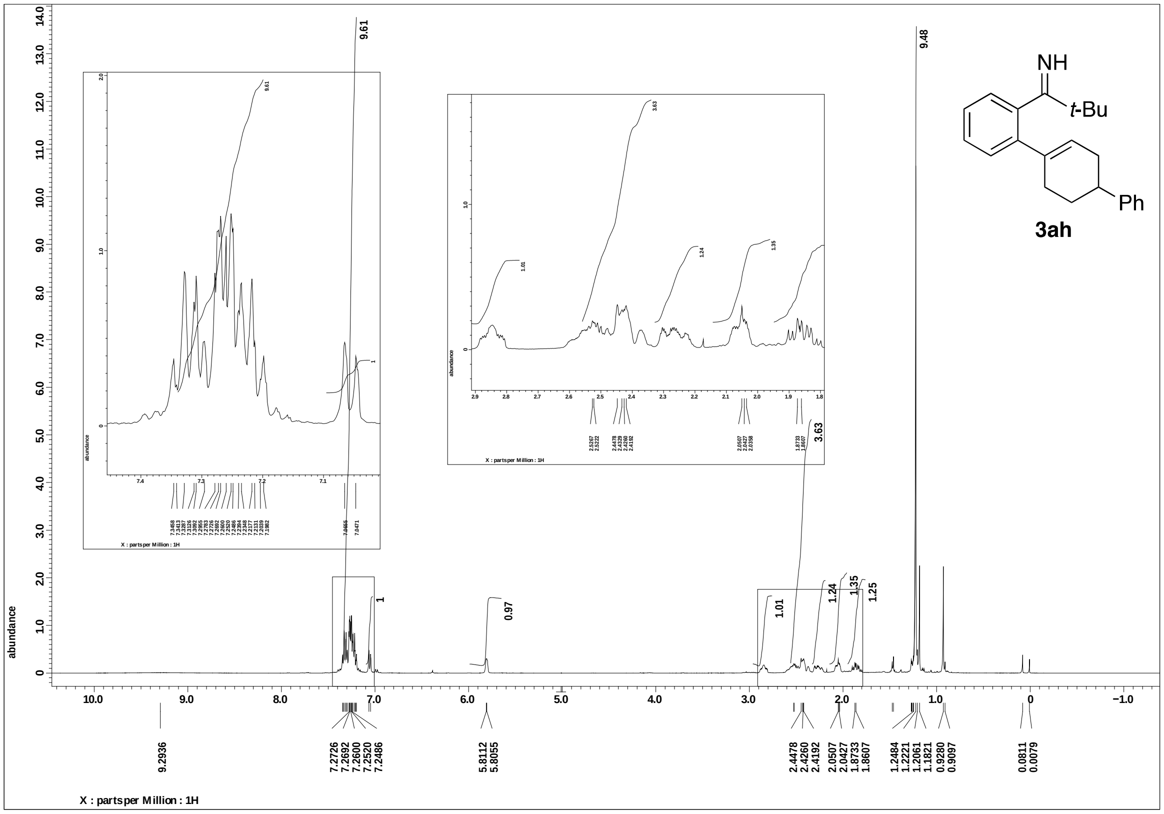
NH
t-Bu
Ph
3ah
abundance
X : partsper Million : 1H
9.61
9.48
1.01
1.24
1.35
1.25
3.63
0.97
9.2936
7.2726
7.2692
7.2600
7.2520
7.2486
5.8112
5.8055
2.4478
2.4260
2.4192
2.0507
2.0427
1.8733
1.8607
1.2484
1.2221
1.2061
1.1821
0.9280
0.9097
0.0811
0.0079

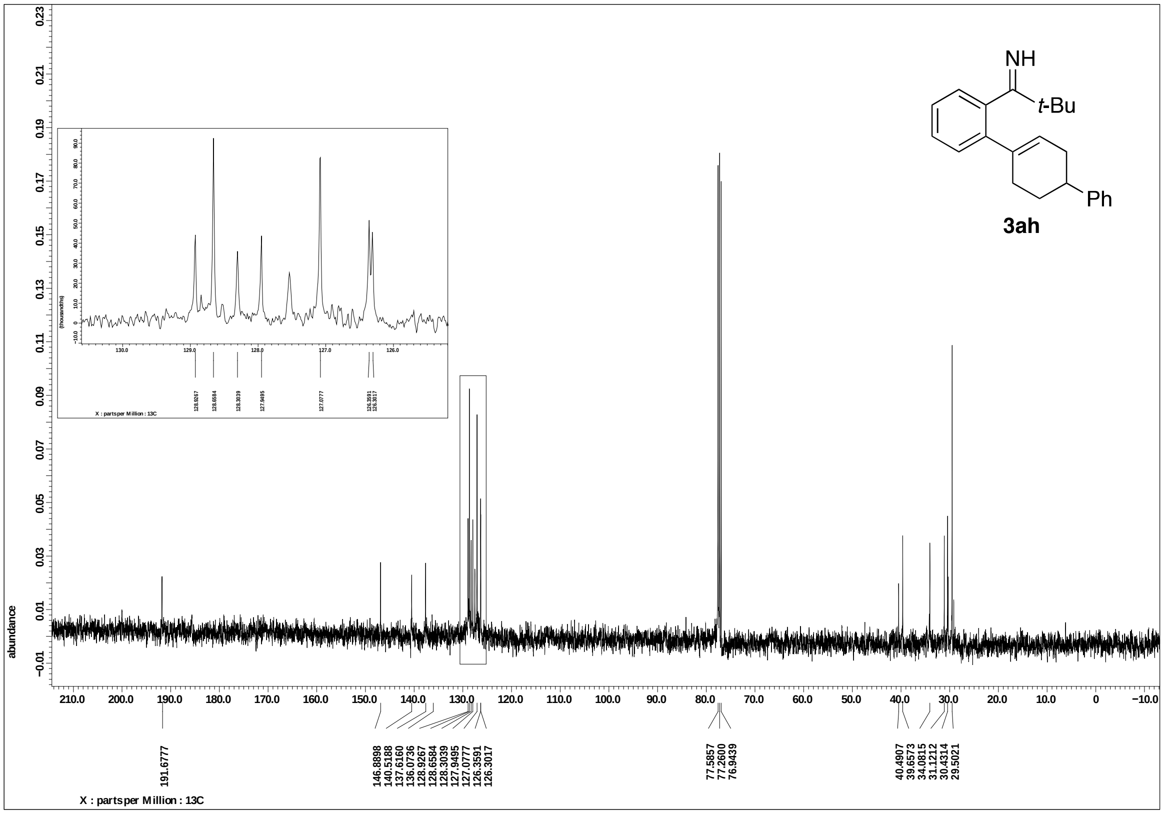
NH
t-Bu
Ph
3ah
abundance
X : partsper Million : 13C
191.6777
146.8898
140.5188
137.6160
136.0736
128.9267
128.6584
128.3039
127.9495
127.0777
126.3591
126.3017
77.5857
77.2600
76.9439
40.4907
39.6573
34.0815
31.1212
30.4314
29.5021

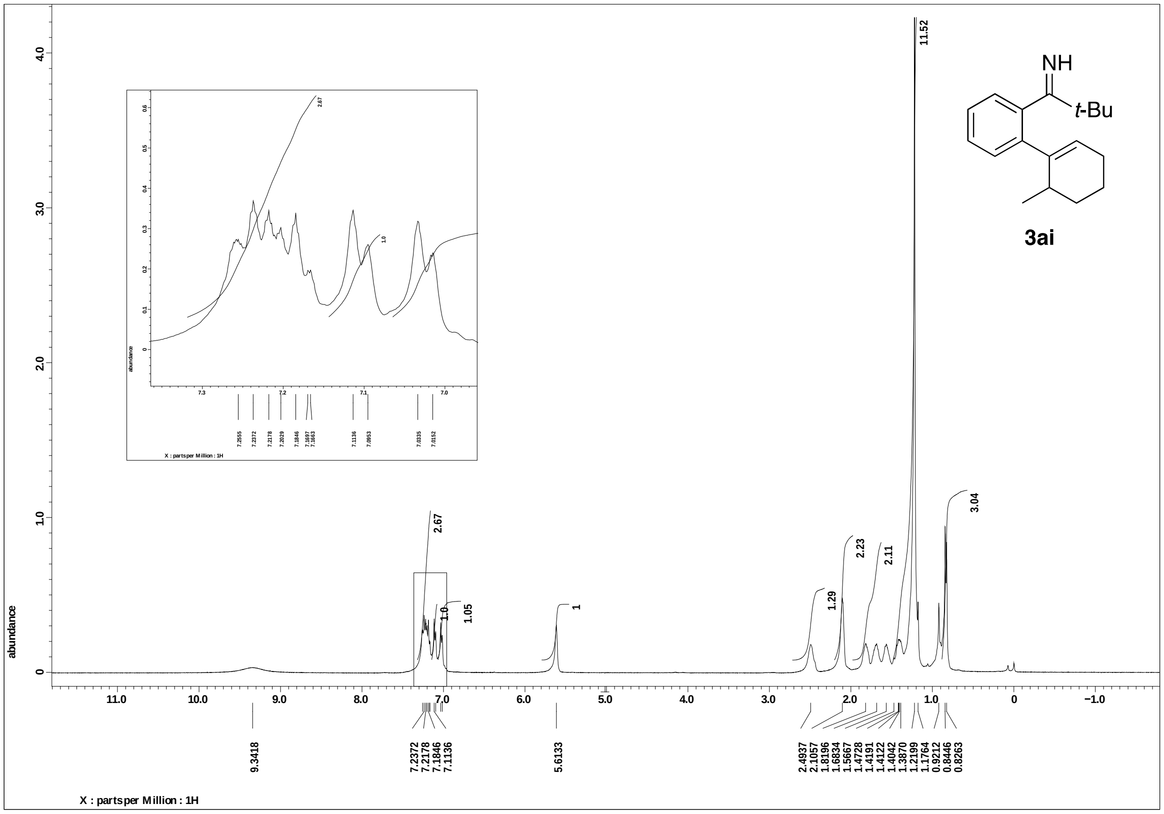
3ai
abundance
X : parts per Million : 1H
9.3418
7.2372
7.2178
7.1846
7.1136
5.6133
2.4937
2.1057
1.8196
1.6834
1.5667
1.4728
1.4191
1.4122
1.4042
1.3870
1.2199
1.1764
0.9212
0.8446
0.8263
2.67
1.05
1
1.29
2.23
2.11
11.52
3.04

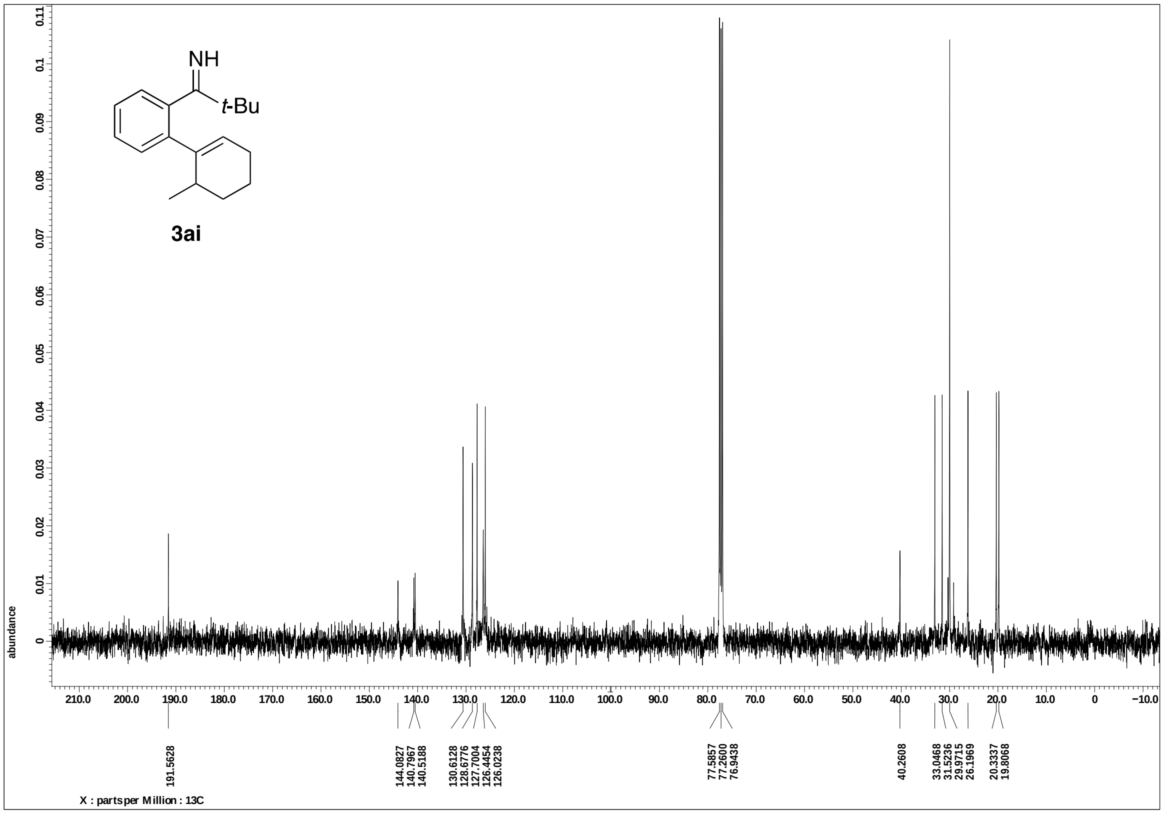
3ai
abundance
X : parts per Million : 13C
191.5628
144.0827
140.7967
140.5188
130.6128
128.6776
127.7004
126.4454
126.0238
77.5857
77.2600
76.9438
40.2608
33.0468
31.5236
29.9715
26.1969
20.3337
19.8068

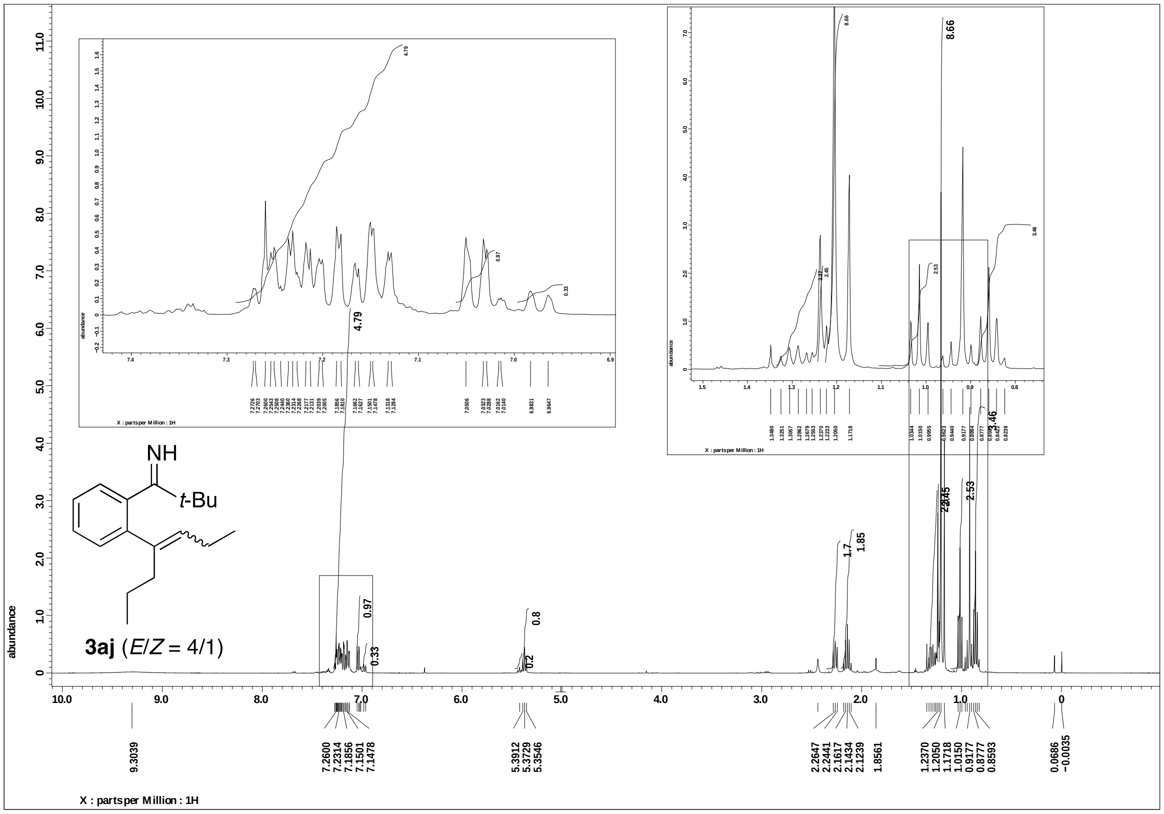
NH
t-Bu
3aj (E/Z = 4/1)
4.79
0.97
0.33
0.8
0.2
1.7
1.85
2.45
2.53
8.66
3.46
9.3039
7.2600
7.2314
7.1856
7.1501
7.1478
5.3912
5.3729
5.3546
2.2647
2.2441
2.1617
2.1434
2.1239
1.8561
1.2370
1.2050
1.1718
1.0150
0.9177
0.8777
0.8593
0.0686
−0.0035
abundance
X : parts per Million : 1H

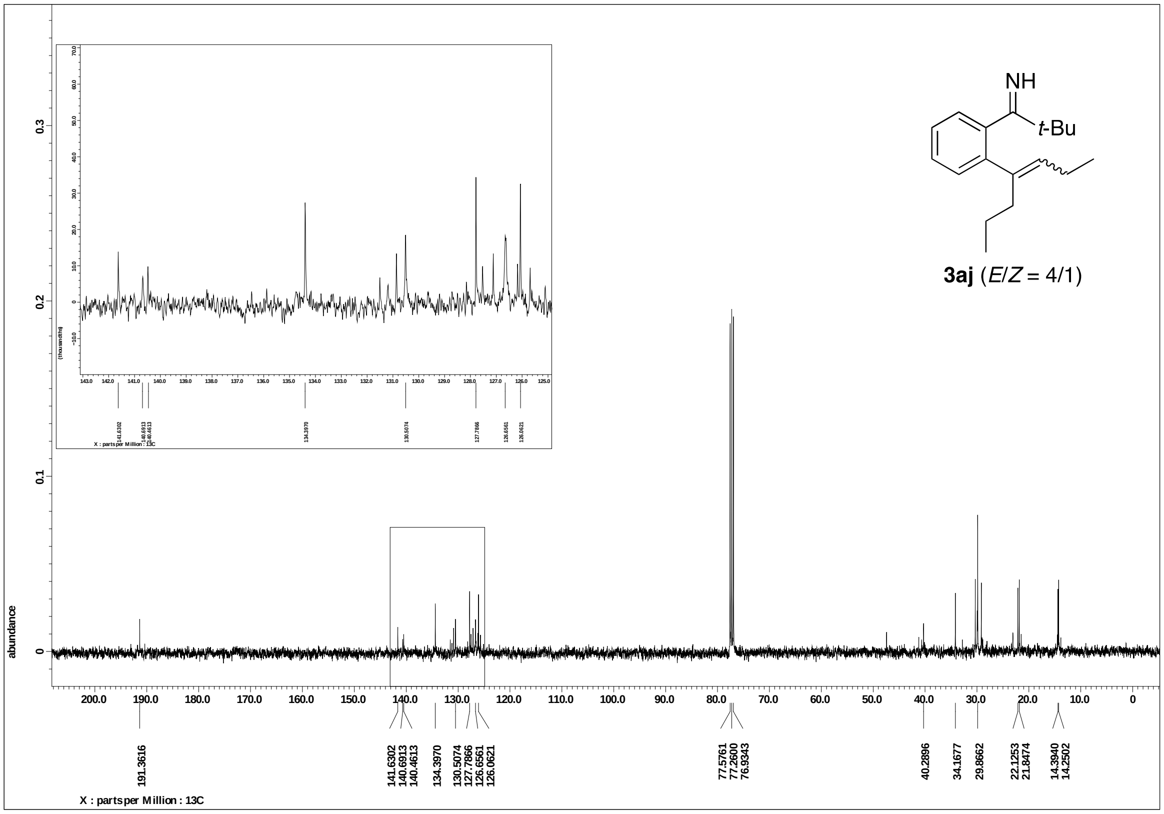
NH
t-Bu
3aj (E/Z = 4/1)
191.3616
141.6302
140.6913
140.4613
134.3970
130.5074
127.7866
126.6561
126.0621
77.5761
77.2600
76.9343
40.2896
34.1677
29.8662
22.1253
21.8474
14.3940
14.2502
abundance
X : parts per Million : 13C

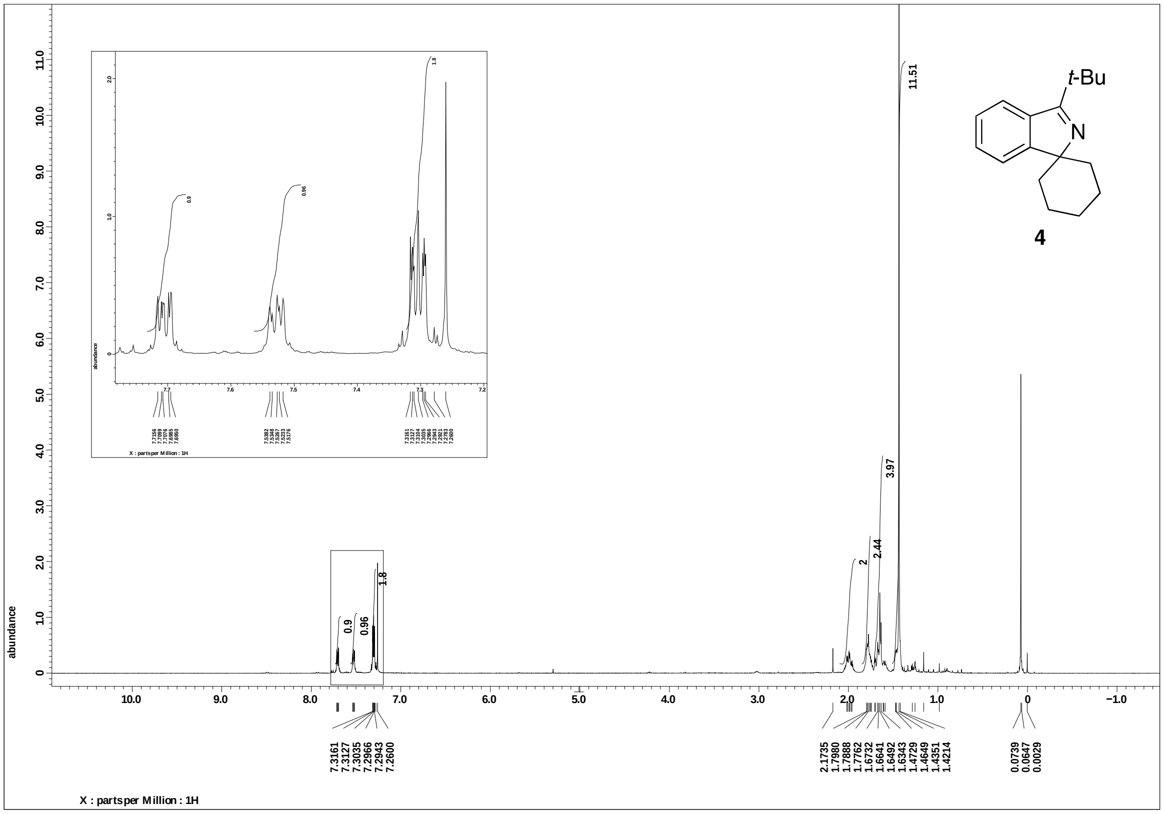
t-Bu
N
4
abundance
X : partsper Million : 1H
11.51
3.97
2.44
2
1.8
0.96
0.9
7.3161
7.3127
7.3035
7.2966
7.2943
7.2600
2.1735
1.7980
1.7888
1.7762
1.6732
1.6641
1.6492
1.6343
1.4729
1.4649
1.4351
1.4214
0.0739
0.0647
0.0029

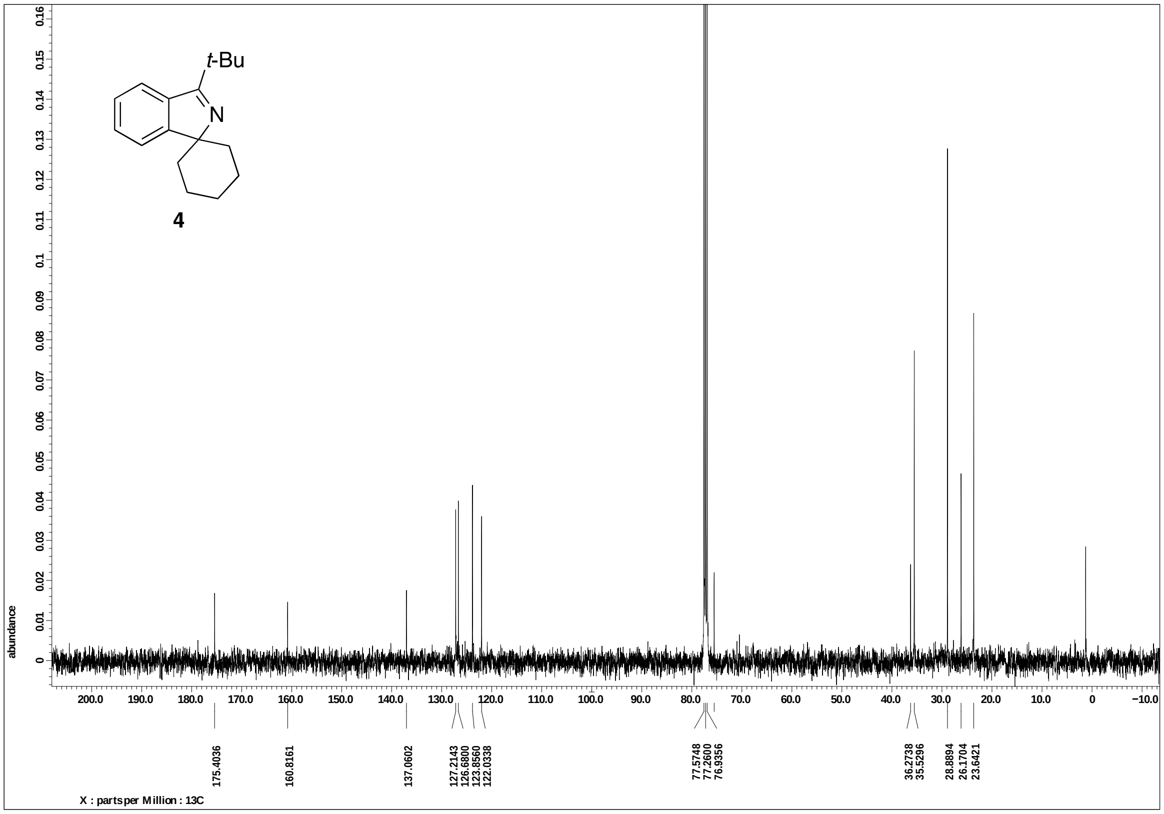
t-Bu
N
4
abundance
X : partsper Million : 13C
175.4036
160.8161
137.0602
127.2143
126.6800
123.8560
122.0338
77.5748
77.2600
76.9356
36.2738
35.5296
28.8894
26.1704
23.6421
